# High Resolution Genome Wide Binding Event Finding and Motif Discovery Reveals Transcription Factor Spatial Binding Constraints

**DOI:** 10.1371/journal.pcbi.1002638

**Published:** 2012-08-09

**Authors:** Yuchun Guo, Shaun Mahony, David K. Gifford

**Affiliations:** 1 Computational and Systems Biology Program, Massachusetts Institute of Technology, Cambridge, Massachusetts, United States of America; 2 Computer Science and Artificial Intelligence Laboratory, Massachusetts Institute of Technology, Cambridge, Massachusetts, United States of America; University of Leuven, Belgium

## Abstract

An essential component of genome function is the syntax of genomic regulatory elements that determine how diverse transcription factors interact to orchestrate a program of regulatory control. A precise characterization of *in vivo* spacing constraints between key transcription factors would reveal key aspects of this genomic regulatory language. To discover novel transcription factor spatial binding constraints *in vivo*, we developed a new integrative computational method, genome wide event finding and motif discovery (GEM). GEM resolves ChIP data into explanatory motifs and binding events at high spatial resolution by linking binding event discovery and motif discovery with positional priors in the context of a generative probabilistic model of ChIP data and genome sequence. GEM analysis of 63 transcription factors in 214 ENCODE human ChIP-Seq experiments recovers more known factor motifs than other contemporary methods, and discovers six new motifs for factors with unknown binding specificity. GEM's adaptive learning of binding-event read distributions allows it to further improve upon previous methods for processing ChIP-Seq and ChIP-exo data to yield unsurpassed spatial resolution and discovery of closely spaced binding events of the same factor. In a systematic analysis of *in vivo* sequence-specific transcription factor binding using GEM, we have found hundreds of spatial binding constraints between factors. GEM found 37 examples of factor binding constraints in mouse ES cells, including strong distance-specific constraints between Klf4 and other key regulatory factors. In human ENCODE data, GEM found 390 examples of spatially constrained pair-wise binding, including such novel pairs as c-Fos:c-Jun/USF1, CTCF/Egr1, and HNF4A/FOXA1. The discovery of new factor-factor spatial constraints in ChIP data is significant because it proposes testable models for regulatory factor interactions that will help elucidate genome function and the implementation of combinatorial control.

## Author Summary

The letters in our genome spell words and phrases that control when each gene is activated. To understand how these words and phrases function in health and disease, we have developed a new computational method to determine what word positions in our genomic text are used by each genome regulatory protein, and how these active words are spaced relative to one another. Our method achieves exceptional spatial accuracy by integrating experimental data with the text of our genome to find the precise words that are regulated by each protein factor. Using this analysis we have discovered novel word spacings in the experimental data that suggest novel genome grammatical control constructs.

## Introduction

Genomic sequences facilitate both cooperative and competitive regulatory factor-factor interactions that implement cellular transcriptional regulatory logic. The functional syntax of DNA motifs in regulatory elements is thus an essential component of cellular regulatory control. Appropriately spaced motifs can facilitate cooperative homo-dimeric or hetero-dimeric factor binding, while overlapping motifs can implement competitive binding by steric hindrance. Cooperative and competitive binding are an integral part of complex cellular regulatory logic functions [1], [2]. The binding of regulatory proteins to the genome cannot at present be predicted from primary DNA sequence alone as chromatin structure, co-factors, and other mechanisms make the prediction of *in vivo* binding from sequence empirically unreliable [3]. Thus it is not possible to use primary DNA sequence to determine the aspects of genome syntax that are employed *in vivo*.

To discover novel pair-wise factor spatial binding constraints *in vivo*, we have developed a new method called GEM that simultaneously resolves the location of protein-DNA interactions and discovers explanatory DNA sequence motifs with an integrated model of ChIP-Seq or ChIP-exo reads and proximal DNA sequences. We define a binding event location as the single base position at the center of a protein-DNA interaction. GEM reciprocally improves motif detection using binding event locations, and binding event predictions using discovered motifs. In doing so, GEM offers a more principled approach than simply snapping binding event predictions to the closest instance of the motif, and indeed, GEM does not require that all binding events are associated with strong motifs. GEM offers both improved spatial accuracy of binding event predictions and improved motif discovery in ChIP-Seq and ChIP-exo datasets.

GEM's unbiased computational approach has enabled us to discover novel binding constraints between transcription factors from sequenced ChIP experiments. These spatial constraints directly suggest biological regulatory mechanisms that will be useful in future studies. Other methods to resolve binding events in sequenced ChIP data identify statistically enriched regions of ChIP-Seq read density and the peak points of enrichment within those regions [4]–[9], and binding calls can be offset from the bound site by dozens of bases [10]. Recent studies have integrated peak detection and motif discovery by including motif occurrences to score the significance of predicted binding events [11], [12], or by using ChIP-Seq read coverage as a positional prior to improve motif discovery [13], [14]. However, no study has yet used the motif position information to reciprocally improve the spatial accuracy of binding event prediction. SpaMo studied the motif spacing using ChIP-Seq events to infer transcription factor complexes but the predicted motif spacing does not necessarily indicate *in vivo* binding in the specific cellular conditions [15].

Here we review our GEM derived results, discuss these results in the context of current data production projects, and detail our methods.

## Results

### GEM improves the spatial resolution of binding event prediction

We compared GEM's spatial resolution to six well known ChIP-Seq analysis methods, including GPS [8], SISSRs [6], MACS [4], cisGenome [7], QuEST [5] and PeakRanger [9]. We used a human Growth Associated Binding Protein (GABP) ChIP-Seq dataset for our evaluation because GABP ChIP-Seq data were previously reported to contain homotypic events where the reads generated by multiple closely spaced binding events overlap [5]. Thus the GABP dataset offers the opportunity to test if integrating motif information and binding event prediction improves our ability to deconvolve closely spaced binding events with greater accuracy. We also evaluated the methods using ChIP-Seq data from the insulator binding factor CTCF (CCCTC-binding factor) [16], as it binds to a stronger motif than GABP. These two factors are representative of relatively easy (CTCF) and difficult (GABP) cases for ChIP-Seq data analysis. They are also used by other studies as benchmarks allowing for the direct evaluation of our results. GEM performance on other factors may vary.

**Figure 1:**
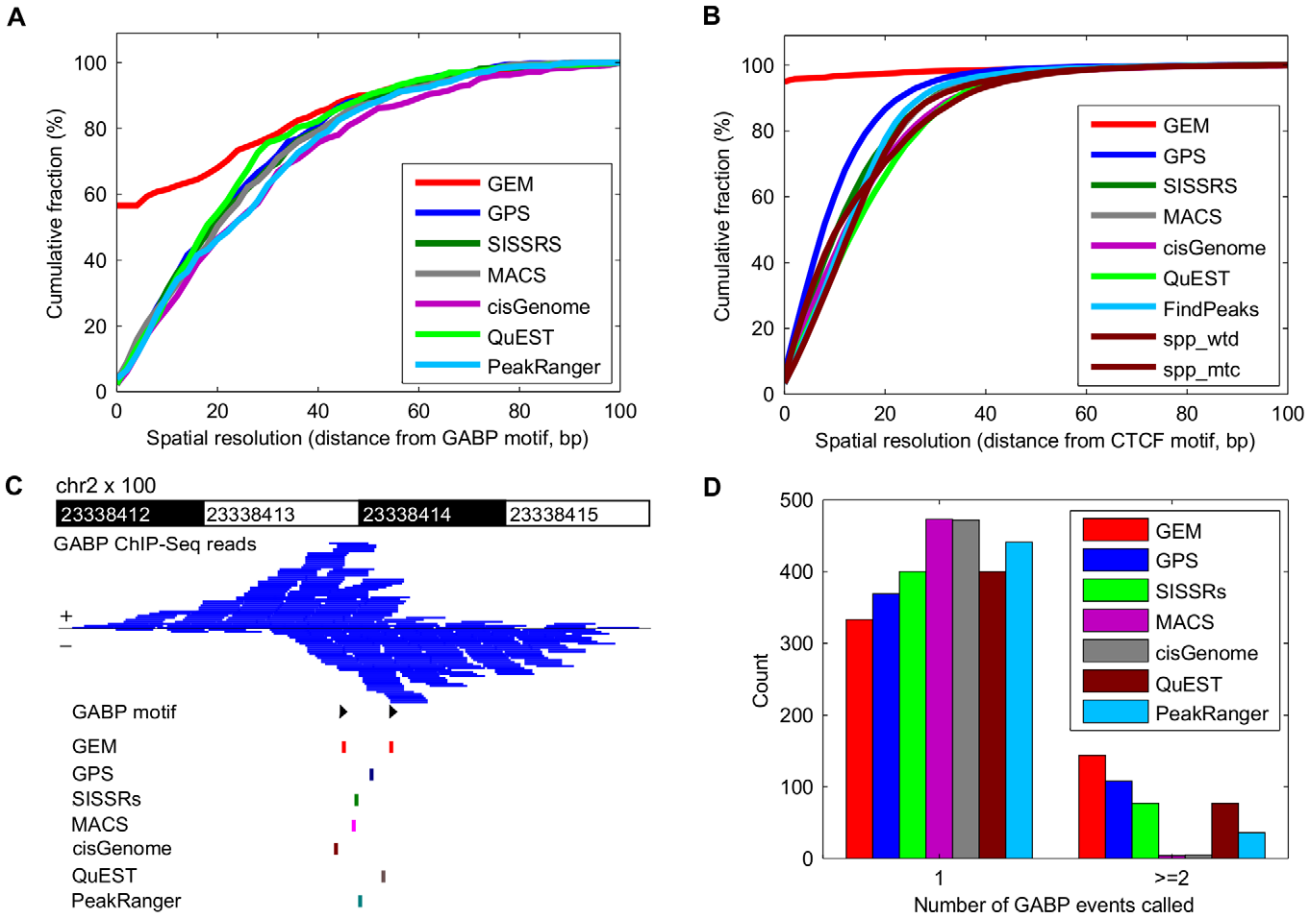
GEM improves spatial accuracy in binding event prediction and the resolution of proximal binding events. **A**) Fraction of predicted GABP binding events with a motif within the given distance following event discovery by GEM, GPS, SISSRs, MACS, cisGenome, QuEST and PeakRanger. Events shown were predicted by all seven methods and had a GABP motif within 100 bp. **B**) Fraction of predicted CTCF binding events with a motif within the given distance following event discovery by GEM, GPS, SISSRs, MACS, cisGenome, QuEST, FindPeaks, spp-wtd and spp-mtc. Events shown were predicted by all nine methods and had a CTCF motif within 100 bp. **C**) Example of a predicted binary GABP event that contains coordinately located GABP motifs. **D**) Numbers of GABP binding events discovered by GEM, GPS, SISSRs, MACS, cisGenome, QuEST and PeakRanger in 477 regions that contain clustered GABP motifs within 500 bp.

We found that GEM has the best spatial resolution among tested methods. Spatial resolution is the average absolute value difference between the computationally predicted locations of binding events and the nearest match to a proximal consensus motif. From all observations, spatial resolution is corrected for a fixed offset by subtracting the mean difference before averaging the absolute value differences. To ensure a fair comparison, we used 428 shared GABP binding sites that are predicted by all seven tested methods and which contain an instance of the GABP motif within 100 bp. GEM exactly locates the events at the motif position in 56.5% of these events (Figure 1A). For a dataset with a stronger consensus motif, ChIP-Seq data from CTCF, GEM exactly locates the events at the motif position in more than 90% of the shared events, significantly improving the spatial accuracy of predicted binding events over other methods (Figure 1B). Alternative evaluations with all the binding sites that have a motif at a distance less than 100 bp are also performed for both GABP and CTCF data, and the results (Figure S1) are similar to those above. Thus, GEM's joint model of ChIP-Seq read coverage and sequence is able to more accurately predict the location of binding sites than other approaches, which do not use motif information in their binding event predictions.

GEM is also better at resolving closely spaced binding events [17] in the GABP data than the other methods we tested. For example, GEM uniquely detects two GABP events over proximal GABP motifs that are 32 bp apart on chromosome 2 (Figure 1C). To evaluate binding deconvolution on a genome-wide scale, we identified 477 candidate clusters of closely spaced binding events. Each candidate cluster was detected as bound by all seven tested methods and contained two or more proximal GABP motifs separated by less than 500 bp. GEM identified two or more closely spaced events in 144 of the candidate clusters, significantly more than GPS(108), SISSRs(77), QuEST(77), PeakRanger(36), MACS(4)and cisGenome(5) (Figure 1D).

### GEM accurately discovers DNA-binding motifs in ENCODE ChIP-Seq data

We tested GEM's ability to discover biologically relevant DNA-binding motifs in data from the ENCODE project [18]. We chose this large collection of experiments because we expected they would be representative of the typical range of ChIP-Seq data noise and sequencing depth. Noise can be caused by low antibody affinity and deviations from ideal experimental procedure. We used a set of 214 ChIP-Seq experiments and associated controls comprising 63 distinct transcription factors that were profiled in one or more cell lines by the ENCODE project and for which validated DNA-binding motifs exist in public databases (Dataset S1). GEM analyzed these ChIP-Seq data, and the most significant GEM-discovered motifs from each analysis (Table S1 and Dataset S2) were compared to corresponding known binding preferences of the same transcription factors using STAMP [19]. A motif alignment with E-value less than 1e-5 was considered a match. For comparison, we also used four popular traditional motif discovery tools covering a range of computational techniques, including MEME [20], Weeder [21], MDScan [22], and AlignACE [23], and three ChIP-Seq oriented tools, POSMO [24], HMS [13] and ChIPMunk [14] on the same data. A set of 100 bp sequences extracted from the 500 most highly ChIP-enriched GPS peaks calls are examined by the motif-finders MEME, Weeder, MDScan, AlignACE, or POSMO. For HMS and ChIPMunk, a set of 100 bp sequences and corresponding read coverage profiles are extracted from the 500 most highly ChIP-enriched GPS peaks calls.

**Figure 2:**
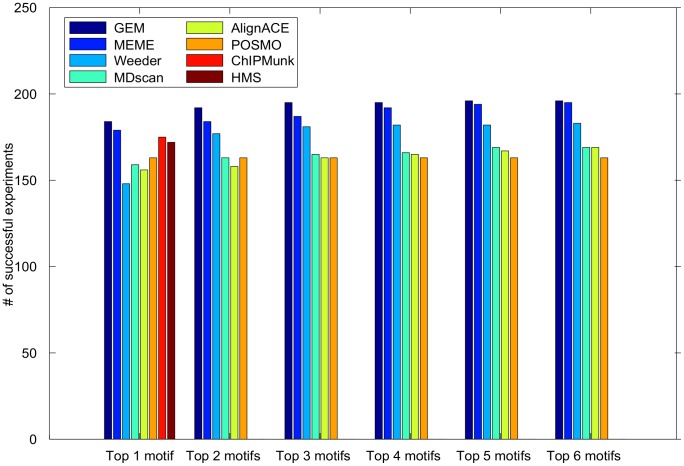
GEM motif discovery outperforms other methods when detecting motifs in ChIP-Seq data. The motif detection performance of GEM is compared to the motif detection performance of various motif-finders on 214 ENCODE ChIP-Seq experiments.

We found GEM outperforms all of the compared motif discovery approaches, even when allowing each method to make multiple motif predictions (Figure 2, Table S2, S3). Therefore, the GEM approach to integrating ChIP-Seq event detection with motif analysis not only improves the spatial resolution of binding events, but also more accurately finds the expected binding motifs present at those events. We note that GEM sometimes failed to find the known motif in datasets where one of the other algorithms succeeds. The complete evaluation is in Table S2, S3.

We then tested GEM on ENCODE ChIP-Seq experiments for 9 distinct transcription factors with no publically described DNA binding motif. For 6 of these transcription factors, GEM discovers novel motifs that are consistent with expected binding sequences based on a small number of binding sites characterized in the literature, or similarity to the known binding preferences of related proteins (Table S4). For example, GEM confirms that BATF has a similar binding preference to other members of the AP1 family of transcription factors. The similar TGAC/G binding preference has previously been supported by EMSA assays on regions upstream potential BATF regulated genes [25].

### GEM improves the spatial resolution of ChIP-exo binding event prediction

**Figure 3:**
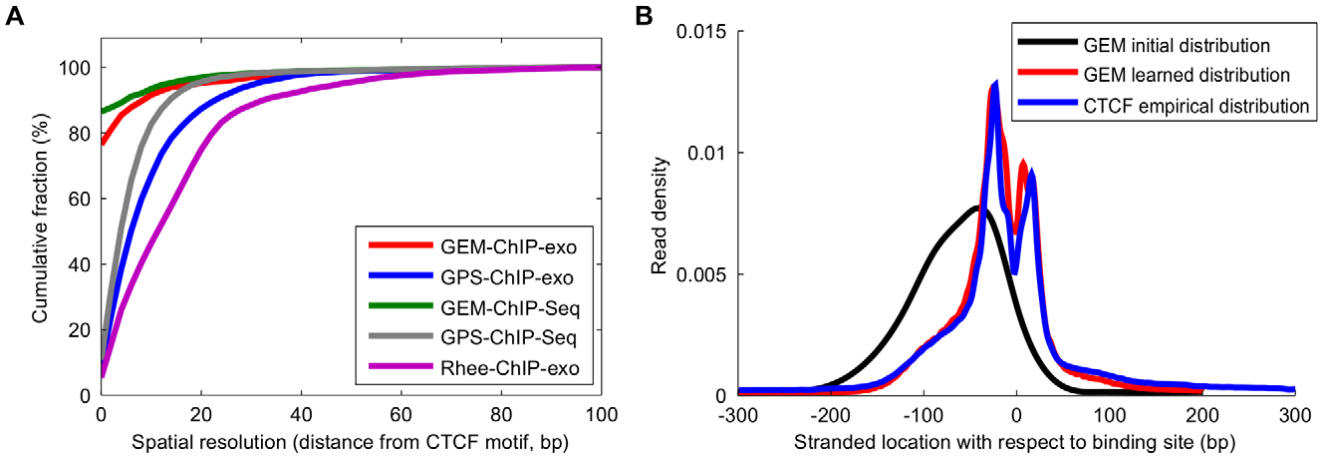
GEM improves the spatial resolution of ChIP-exo data event prediction. **A**) Fraction of predicted CTCF binding events with a motif within the given distance following event discovery by GEM, GPS, and the peak-pair midpoint method of Rhee, et al. **B**) GEM automatically adapts to the ChIP-exo read spatial distribution.

ChIP-exo aims to improve transcription factor binding spatial resolution by extensively digesting ChIP fragments down to the DNA that is protected by the bound protein complex [26]. While ChIP-exo experiments provide high-resolution binding information, typical peak-finding methodologies may fail to achieve single-base resolution binding event predictions if they do not account for the properties of the ChIP-exo experiment. An example is provided by the published CTCF ChIP-exo experiment [26], where ChIP-exo reads are bimodally distributed around binding sites on both strands because CTCF is cross-linked at two distinct sites of DNA. The published event predictions did not account for this characteristic distribution, and are thus often offset from CTCF binding motif instances. Since GPS and GEM automatically learn a model of sequence reads around binding events, GPS and GEM may be directly applied to ChIP-exo data without modification. We first verified that GEM's model of binding events is able to automatically adapt to the read distribution produced by the ChIP-exo protocol. We compared GEM's final computed read distribution to the expected empirical distribution of ChIP-exo and found that they were consistent (Figure 3B and Figure S2).

GEM improves upon the spatial resolution of binding event detection over other methods for ChIP-exo data (Figure 3A). To investigate the performance of GEM on ChIP-exo data, we compared the binding event predictions of GEM and GPS on ChIP-exo CTCF binding and the “middle of peak-pair” method from the original ChIP-exo study [26]. To ensure a fair comparison, we used 5074 shared binding sites that are predicted by all tested methods and that contain a strong CTCF motif match within 100 bp of the binding positions. The original ChIP-exo study [26] had 5.4% of the binding event calls centered on the motif match position, 40.3% of the calls within 10 bp, and an average spatial resolution of 15.85±15.29 bp. Applying GPS to the ChIP-exo data improved the spatial resolution, with 8.8% calls at 0 bp positions, 59.7% of calls within 10 bp, and average spatial resolution of 10.38±11.26 bp. Applying GEM to the ChIP-exo data located 76.5% calls exactly at the motif match positions, 89.7% of calls within 10 bp, and an average spatial resolution of 3.35±9.71 bp. These results demonstrate that GEM can significantly improve the spatial accuracy of ChIP-exo binding event predictions.

### GEM reveals known Sox2-Oct4 distance-constrained transcription factor binding distances

We examined if GEM could detect pairs of transcription factors that bind to the genome with characteristic pair-wise spacing, beginning with the well-known hetero-dimeric pair Sox2-Oct4 [27]. In general, distance-constrained transcription factor binding cannot be predicted based solely on sequence motifs as motif presence does not guarantee binding. Such spatial binding constraints may be caused by combinatorial binding, alternative binding, binding that is orchestrated by multimeric protein complexes, or the spread of constrained enhancer syntax.

**Figure 4:**
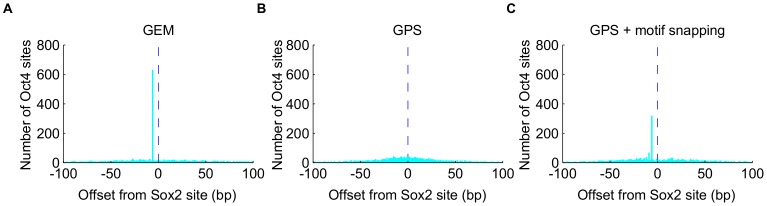
GEM reveals transcription factor spatial binding constraints. **A**), **B**), and **C**) Genome wide spatial distribution of Oct4 binding sites in a 201 bp window around Sox2 binding sites, obtained by using GEM binding calls, GPS binding calls, or GPS binding calls snapping to the nearest motifs within 50 bp, respectively. Dashed lines represent the Sox2 binding sites at position 0.

We were able to discover Sox2-Oct4 transcription factor spatial binding constraints by combining GEM binding calls from Sox2 and Oct4 ChIP-Seq data. We applied GEM independently to mouse ES cell Sox2 and Oct4 ChIP-Seq data [15] to call the respective binding sites, and then computed the distance between Oct4 sites from Sox2 sites within a 201 bp window. The sequence strand of the GEM binding predictions is oriented using the Sox2 motif when a match to the motif is present. As expected, GEM predicted Oct4 binding sites are predominantly (630 sites out of 2525 in the 201 bp window) located at −6 bp position relative to GEM predicted Sox2 sites (Figure 4A and Figure S3). However, this spacing cannot be observed from the binding calls of GPS or other event discovery methods alone because of their more limited spatial accuracy (Figure 4B). An alternative approach is to snap binding calls to the nearest instance of the transcription factor's binding motif. We tested this approach using GPS binding calls as the starting points and found that the alternate approach captures fewer (277 sites out of 2753) instances of Oct4-Sox2 spatial binding constraints (Figure 4C), presumably because some of the bound motifs do not pass the motif scoring threshold or because some unbound motif instances are located closer to the binding calls than the true motif instances.

### Enhancer grammar elements deduced from transcription factor binding sites predicted by GEM

We next studied pair-wise binding relationships between 14 sequence-specific transcription factors (Oct4, Sox2, Nanog, Klf4, STAT3, Smad1, Zfx, c-Myc, n-Myc, Esrrb, Nr5a2, Tcfcp2l1, E2f1 and CTCF) and two transcriptional regulators (p300 and Suz12) in mouse ES cells by applying GEM to a large compendium of ChIP-Seq binding data [16], [28]. Binding prediction by GEM enables the detection of 37 pairs of statistically significant spatial binding constraints, involving Oct4, Sox2, Nanog, Klf4, Esrrb, Nr5a2, Tcfcp2I1, E2f1, c-Myc, n-Myc and Zfx (Figure S4, the full list of TF pairs are in Table S6, S7, motifs are in Table S5 and Dataset S3). Interestingly, we found that Klf4, one of the ES cell reprogramming factors, exhibits strong distance-specific binding with many other factors, including Nanog, Sox2, Zfx, c-Myc, n-Myc, E2f1, Esrrb, Nr5a2 and Tcfcp2l1 (Figure S5).

**Figure 5:**
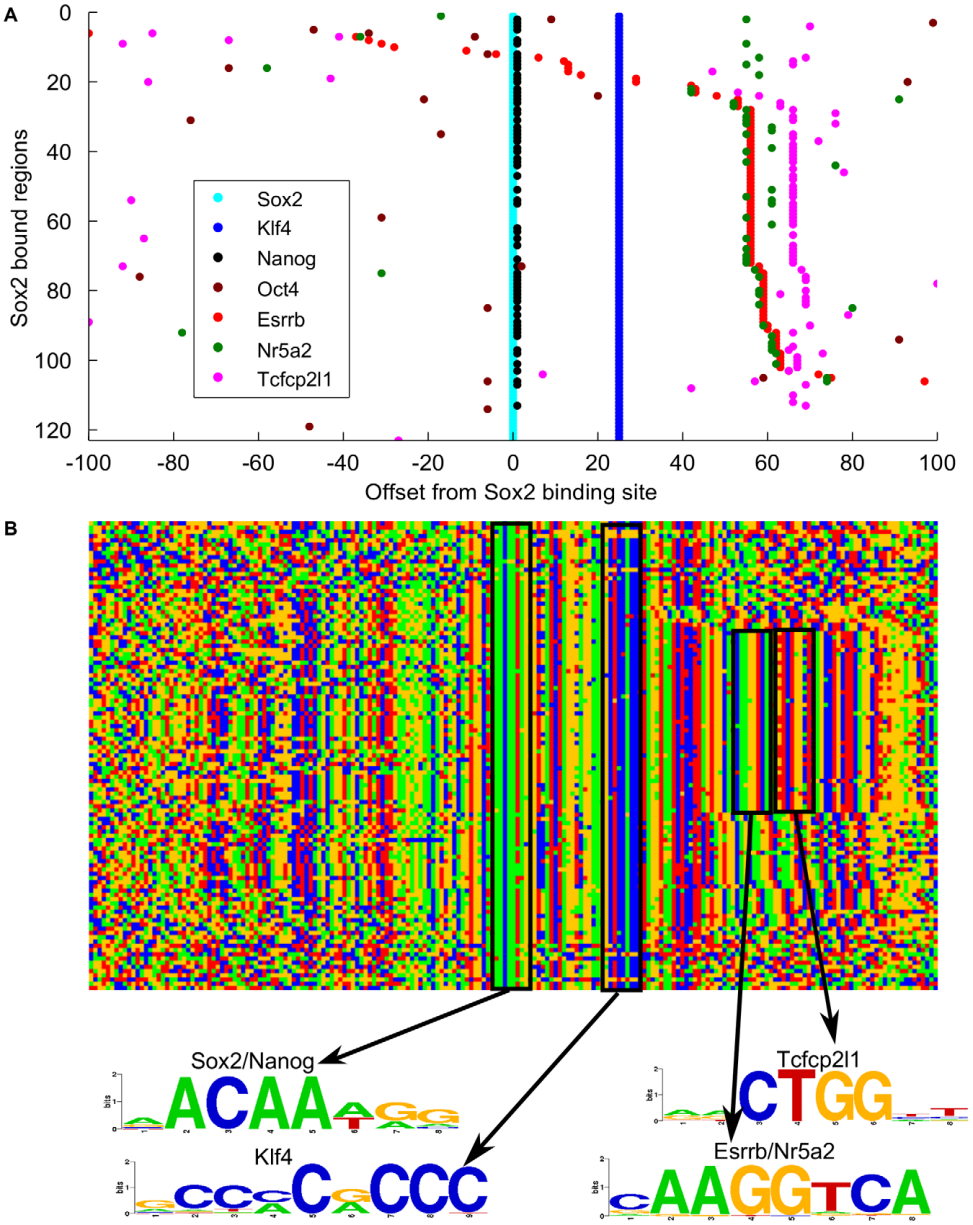
Enhancer grammar elements deduced from mouse ES cell transcription factor binding sites predicted by GEM. **A**) The binding site distribution of Sox2, Klf4, Nanog, Oct4, Esrrb, Nr5a2 and Tcfcp21l in 123 regions that exhibit Sox2-Klf4 spatial binding constraints. The Sox2 sites are aligned at the 0 bp positions, and Klf4 sites are at the 25 bp positions. The rows are ordered by Esrrb offset positions. **B**) Color chart representation of 201 bp sequences in the same regions as in **A**. Each row represents a 201 bp bound sequence. Green, blue, yellow and red indicate A, C, G and T. The motif logos are generated by STAMP [19] from the motifs discovered using all the binding sites in the respective datasets.

The discovered pair-wise spatial binding constraints reveal complex relationships among the factors. For example, Klf4 exhibits constrained binding with Sox2 but much less significantly with Oct4 (Figure S5). However, we did observe strong distance-specific binding between Oct4-Sox2 (Figure 4A). This raises the question of whether the detected Klf4-Sox2 and Oct4-Sox2 spatial binding constraints are on the same genomic regions. We therefore studied all Sox2 bound regions that are co-bound with Klf4. Out of a total of 5609 Sox2 bound regions with a Sox2 motif instance that can be oriented, 123 regions are co-bound by Klf4 at position +25 bp (Figure 5A). However, only four regions show co-binding of Klf4 at position +25 bp and Oct4 at position −6 bp. More surprisingly, the distance-constrained Sox2/Klf4 regions are co-bound by 6 ES cell factors within a 70 bp window, including Sox2 (at 0 bp), Nanog (at 1 bp), Klf4 (at 25 bp), Esrrb (at 56, 59 bp), Nr5a2 (at 55, 58, 61 bp) and Tcfcp2I1 (at 66, 69 bp). Inspecting the underlying sequences of these regions, we found that the binding motifs of these factors are embedded at the positions consistent with the binding positions (Figure 5B). In addition to the consistent spatial arrangement of motifs, these sequences (spanning from −70 bp to 100 bp) exhibit a high degree of similarity. A subset of the sequences is shifted 3 bases by some insertion/deletions, consistent with the 3 bp shift of some of the factor binding positions. Comparing with p300 and H3K27ac ChIP-Seq datasets [29], we found that almost all (119 out of 123) of these regions are bound by p300, a histone acetyltransferase and transcriptional coactivator that predicts tissue-specific enhancers [30]; the majority of these regions are also marked by H3K27ac, a histone modification associated with active enhancers [29], suggesting that they may be active enhancer regions (Figure S6). These results demonstrated that GEM analysis enables detection of coordinated binding of multiple factors that are driven at least partly by the underlying sequences.

Of the 123 regions where Sox2, Klf4, and other sites display constrained spacing, 109 (89%) are annotated instances of the RLTR9 ERVK family of long terminal repeat elements. It is interesting to note that while Bourque, et al. found an association between Oct4/Sox2 co-binding sites and other members of the ERVK repeat class [31], we find a set of repetitive elements that encode the binding of Sox2 and other factors without Oct4 in ES cells. Kunarso, et al. suggested that transposable elements have rewired the core regulatory network of ES cells [32]. Our analysis found that the repetitive sequences constrain the *in vivo* binding of a number of key transcription factors in ES cells.

### Spatially constrained human factor binding in ENCODE data

We computed statistically significant pair-wise spatially constrained binding events between 46 transcription factors characterized in 184 ENCODE ChIP-Seq data sets in five different cell lines. Each transcription factor ChIP was processed independently by GEM so that we could assess any differences in observed binding between cell lines and biological replicates.

**Figure 6:**
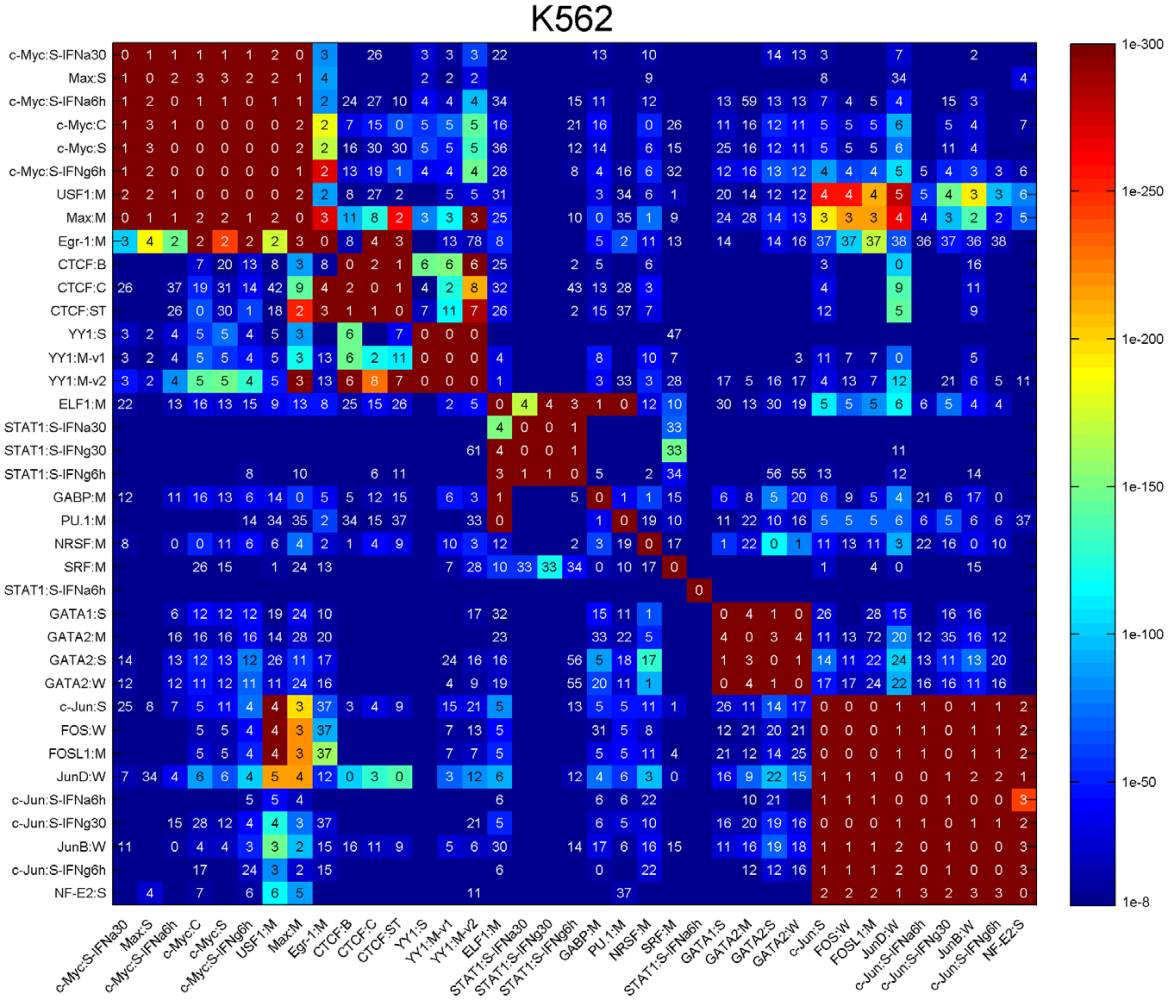
Spatial binding constraints detected from ENCODE ChIP-Seq datasets. Matrix representation of pairwise spatial binding constraints between factor B (column) and factor A (row) detected from 37 ChIP-Seq dataset in human K562 cells. The colors represent the significance levels (corrected p-value) of the strongest spacings. The numbers represent the distances between the factors in the strongest spacings.

**Figure 7:**
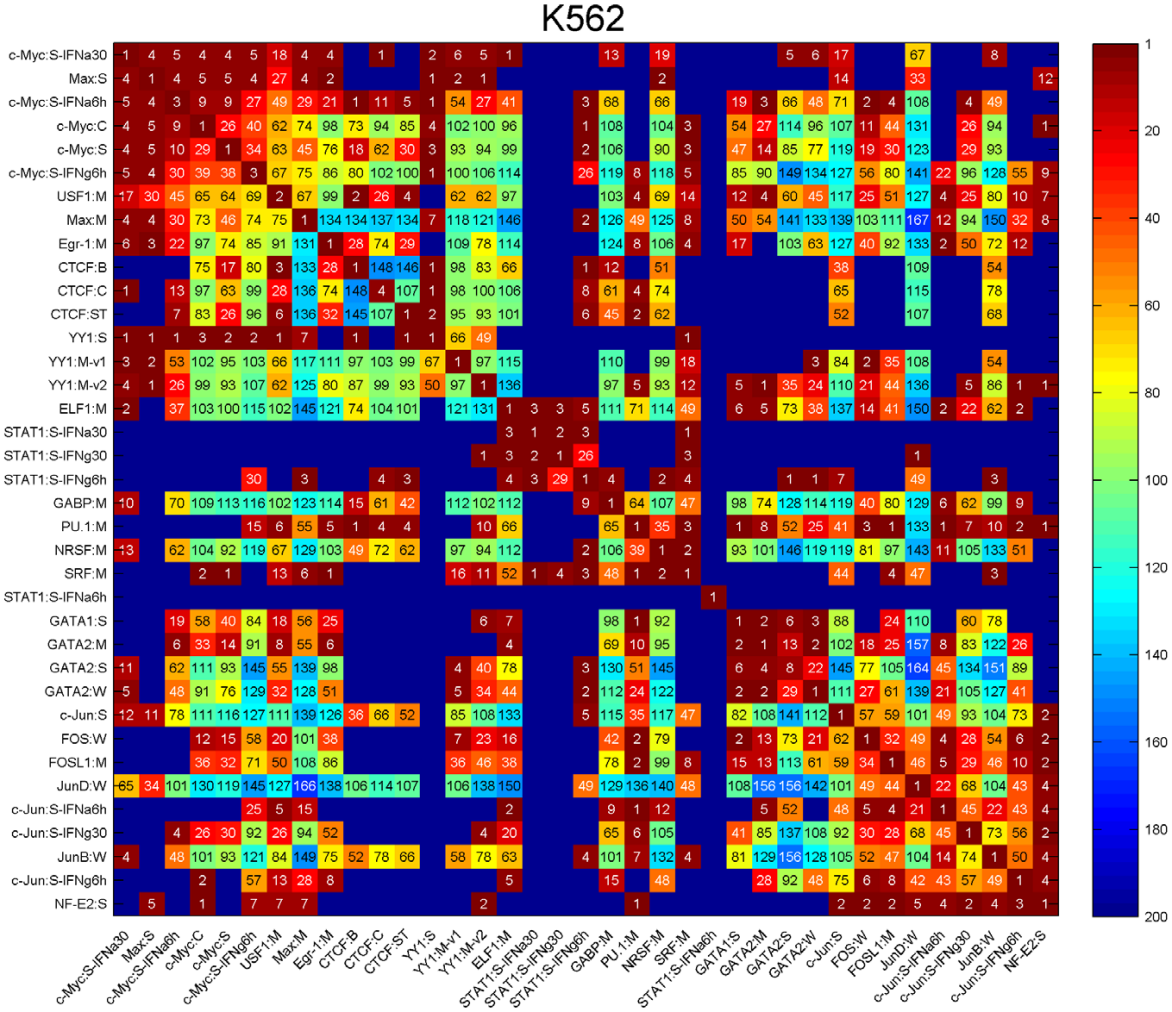
Spatial binding constraints detected from ENCODE ChIP-Seq datasets. Matrix representation of pairwise spatial binding constraints between factor B (column) and factor A (row) detected from 37 ChIP-Seq dataset in human K562 cells. The colors and numbers represent the number of positions exhibiting significant spatial binding constraints within the 201 bp window around the binding sites of factor B (column).

We found that 390 pairs of transcription factors have significant binding distance constraints within 100 bp of each other (Figure 6–7, Figure S7, S8, S9, S10, the full list of TF pairs are in Table S8, S9). The number of pairs found in each cell line differed as did the number of transcription factors assayed: K562 (152 pairs/37 TFs), GM12878 (148 pairs/29 TFs), HepG2 (107 pairs/29 TFs), HeLa-S3 (48 pairs/15 TFs), and H1 (23 pairs/11 TFs). Certain factor-pairs exhibited a highly significant single binding spacing offset within 100 bp, such as the 4 bp distance between Egr1 and CTCF in K562 cells (Figure 6). Other factor pairs exhibited a large number of significant offsets, such as the 167 significant spacings between JunD and Max with the most significant being at 4 bp (Figure 6–7). Our analysis confirmed known interaction pairs MYC-MAX [33], the FOS-JUN heterodimer [34], and CTCF-YY1 [35] (Table S8, S9).

**Figure 8:**
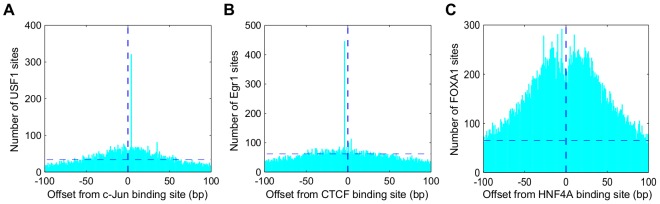
Examples of transcription factor spatial binding constraints detected from GEM analysis. **A**) Genome wide spatial distribution of USF1 binding sites in a 201 bp window around c-Jun binding sites. **B**) Egr1 binding sites around CTCF binding sites. **C**) FOXA1 binding sites around HNF4α binding sites. Vertical dashed lines represent the centered factor binding sites at position 0; horizontal dashed lines represent the number of occurrences at a position corresponding to corrected p-value of 1e−8.

Observed novel genome wide spatial binding constraints include c-Fos:c-Jun/USF1, CTCF/Egr1, HNF4α/FOXA1. We find that USF1 often binds 4 bp from c-Fos:c-Jun (Figure 8A and Figure S11). This binding is consistent with Fra1's facilitation of a complex between USF1 and c-Fos:c-Jun [36]. We find a significant number of cases where CTCF co-binds 4 bp from Egr1 (Figure 8B and Figure S12). Egr1 promotes terminal myeloid differentiation in the presence of deregulated c-Myc expression, and Egr1 has been implicated in down regulating c-Myc in conjunction with CTCF [37]. In addition, the co-binding of CTCF and Egr1 at the EPO regulatory region has been suggested [38]. FOXA1 binds at a large number of significant positions close to HNF4α (total 4215 regions with a spacing within 30 bp, Figure 8C and Figure S13), and there are also significant binding constraints between HNF4α and HNF4γ and FOXA1, FOXA2 in HepG2 cells (Table S8, S9). While co-binding of HNF4α/FOXA2 has been reported [39], co-binding of HNF4α/FOXA1, HNF4γ/FOXA1 and HNF4γ/FOXA2 are not known. We note that HNF4α and any one of FOXA1, FOXA2, or FOXA3 is sufficient to reprogram cells towards a hepatocytic fate [40].

## Discussion

Collectively, our results demonstrate that it is now possible to reveal aspects of functional genome syntax by surveying *in vivo* binding relationships between transcription factors at high spatial resolution. Our analysis has been made possible by sequenced ChIP data and a new computational method, GEM, which provides exceptional spatial resolution.

GEM makes binding predictions and observes spatial constraints by discovering significant events utilizing both motifs and observed read coverage information. Prior work has documented specific genomic regions extensively targeted by multiple transcription factors (TFs) [16]. However, we have shown that the functional syntax of DNA motifs in regulatory elements cannot be fully elaborated with the imprecise ChIP-Seq event calls provided by previous methods. Motif analysis approaches such as SpaMo discover enriched motif spacing by scanning a list of known motifs in sequences anchored by ChIP-Seq data of a single factor [15]. Since the existence of motif instances does not guarantee condition specific *in vivo* binding, SpaMo cannot confidently determine the spacing between binding events and the factors involved, especially for motifs that are shared by a family of TFs. Furthermore, SpaMo excludes repetitive sequences [15]. In contrast, GEM predicts binding based on uniquely-mapped reads and is able to detect spatial binding constraints in transposable elements. Such elements have been implicated in rewiring the core regulatory network of human and mouse ES cells [32].

We expect that the genome grammatical rules that are suggested here will be examined in further studies to elucidate mechanisms of transcriptional control, and potential protein-protein interactions that have regulatory consequences. Exploration of other genome grammatical constructs can be accomplished with the use of further ChIP experiments and GEM.

## Methods

The GEM algorithm consists of six phases:

1. Predict protein-DNA binding event locations with a sparse prior
2. Discover the set of enriched k-mers at binding event locations
3. Cluster the set of enriched k-mers into k-mer equivalence classes
4. Generate a positional prior for event discovery with the most enriched k-mer equivalence class
5. Predict improved protein-DNA binding event locations with a k-mer based positional prior
6. Repeat motif discovery (Steps 2–3) from the Phase 5 improved event locations.

### Predicting protein DNA-binding events with a sparse prior

Initial protein-DNA binding event locations are predicted by GPS [8], which employs a negative Dirichlet sparse prior.

### Discovery of the set of enriched k-mers at binding event locations

GEM discovers a set of enriched k-mers by comparing k-mer frequencies between positive sequences and negative control sequences. The positive set consists of 61 bp sequences centered on the predicted binding locations from Phase 1, and a negative set consists of 61 bp sequences that are 300 bp away from binding locations and that don't overlap positive sequences. We count the number of positive and negative sequences that contain instances of each possible k-mer (hit count), treating each k-mer and its reverse complement as the same sequence. A k-mer is considered enriched if the hypergeometric p-value [41] of its enrichment is less than 0.001 and it has at least 3-fold enrichment in terms of positive/negative hit count. In this study, values of k from 5 to 13 are used on each dataset, and the final k value is chosen as the one that gives the most significantly enriched primary PWM as described below. Each k-mer carries with it its expected offset from a binding event as averaged over the positive set.

### Clustering the enriched k-mers into k-mer equivalence classes

GEM next clusters the enriched k-mers into equivalence classes that describe similar DNA binding preferences (Figure S14). Each equivalence class is a collection of k-mers. A genomic sequence is said to match a k-mer equivalence class if the genomic sequence contains any of its component k-mers. GEM clusters enriched k-mers into k-mer equivalence classes by (Figure S14):

1. A k-mer class is initialized with the most enriched k-mer and any other enriched k-mers that differ by a single base from the most enriched k-mer.
2. Positive set sequences that match the k-mer class are selected, and any enriched k-mer that appears in a 2k+1 bp window around a class match are tested for addition to the class. An enriched k-mer must have the same alignment offset to window sequences in at least one third of its occurrences to be added to the class.
3. A Position Weight Matrix (PWM) is constructed from positive set sequences that match the class. A PWM is constructed with weighted matched positive set sequences centered on the class match and a zero order Markov model learned from negative set sequences. For PWM construction a positive set sequence is weighted by its binding event read count and the distance in bases between the sequence's class match and the estimated binding event position. The distance weighting function we use was fit to characterized ChIP-Seq data, and is the logistic distribution with mean 0 and variance 13. PWMs are trimmed to find the PWM with the most significant hypergeometric p-value between the positive and negative sequences. PWM matching is defined as at least 60% of the maximum PWM score [42].
4. Positive set sequences that match the resulting PWM are extracted and aligned by the PWM instances and any enriched k-mer that appears in a 2k+1 bp window around a PWM match are tested for addition to the class. An enriched k-mer must have the same alignment offset to window sequences in at least 1/3 of its occurrences to be added to the class.
5. Step 3 and 4 are iterated until the PWM hypergeometric p-value between the positive and negative sequences no longer improves.

After finding the primary k-mer equivalence class, GEM searches for other classes. To accomplish this, the previous seed k-mer is removed from the enriched k-mer pool and PWM motif occurrences are masked in the sequences. The process of building new k-mer equivalence classes is repeated until no more significantly enriched PWMs can be constructed. Rarely, a secondary motif PWM can become more significantly enriched than the primary motif. If this happens, the motif finding process is restarted using the seed k-mer of this secondary motif.

### Positional prior generation

Phase 4 of GEM uses the primary k-mer equivalence class to compute a Dirichlet prior that will be used for binding event discovery in Phase 5. The genome is segmented into independent separable regions (typically a few kb long) by dividing at read gaps that are larger than 500 bp and further excluding regions that contain fewer than 6 reads [8]. At each evaluated genome region, we simultaneously search the occurrences of all the k-mers of the primary k-mer equivalence class using the Aho-Corasick algorithm [43], and matches are marked at the expected binding event location for every matching k-mer. The position-specific prior for a sequence base is defined as the number of positive set sequences that contain one of the enriched k-mers whose binding offsets match that base. The concept of using informative positional priors for motif discovery has been explored previously [44], [45].

### Binding event prediction with a positional prior

GEM employs a generative mixture model that describes the likelihood of a set of ChIP-Seq reads being generated from a set of protein-DNA interaction events originating at specific DNA sequences. The model generates protein-DNA interaction events that are biased to occur at explanatory DNA sequences by a k-mer based positional prior. Each event then independently generates reads following an empirical read spatial distribution that describes the probability of reads given the distance from the event [8] (see Figure 3B for an example).

Formally, in an evaluated region of length *M*, we consider *N* ChIP-Seq reads that have been mapped to genome locations ***R*** = {*r*_1_, …, *r_N_*} and *M* all possible protein-DNA interaction events at single base locations ***B*** = {*b*_1_, …, *b_M_*}. We represent the latent assignments of reads to events that caused them as ***Z*** = {*z*_1_, …, *z_N_*}, where indicator function **1**(*z_n_* = *m*) = 1 when read *n* is caused by the event *m*.

The probability of a read *n* is based on a mixture of possible binding events:


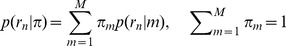


where *M* is the number of possible events; ***π*** denotes the parameter vector of mixing probabilities, and *π_m_* is the probability of event *m*; *p*(*r_n_* | *m*) is the probability of read *n* being generated from event *m* and can be determined from the empirical spatial distribution of reads given the event [8].

The overall likelihood of the observed set of reads is:


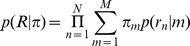


We make two prior assumptions about the binding events: 1) binding events prefer to occur at the sequence specific DNA motif positions; 2) binding events are relatively sparse throughout the genome. To incorporate these assumptions, we place a negative Dirichlet prior [8], [46] *p*(***π***) on binding event probabilities ***π***:


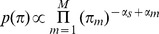


where *α_s_* is the uniform sparse prior parameter governing the degree of sparseness, *α_s_*>0; *α_m_* denotes the binding event specific prior parameter and its value is proportional to *C_m_*, the positional prior count underlying event *m* (as defined in Phase 4):


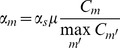


where *μ* is a parameter to tune the effect of motif based prior, 0≤*μ*<1. In this study, we choose *μ* = 0.8.

The rationale is that if the k-mers mapped to position *m* have more occurrences at binding events genome wide, it is more likely to cause a binding event at that genome position. The parameter *α_m_* is scaled such that all the values of possible *α_m_* will be less than *α_s_*. Therefore the k-mer based prior will not force the model to predict a binding event at a motif position when the observed reads do not provide sufficient evidence of a protein-DNA interaction event.

Since the k-mers underlying the possible binding event positions and their counts are known, the value of term −*α_s_*+*α_m_* remains constant when we estimate the parameters in the mixture model. Therefore, we can solve the mixture model using Expectation-Maximization (EM) algorithm [47].

The complete-data log penalized likelihood is:


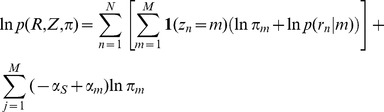


where **1**(*z_n_* = *m*) is the indicator function.

In the E Step we have:


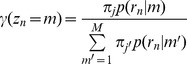


where **γ**(*z_n_* = *m*) can be interpreted as the fraction of read *n* that is assigned to event *m*.

In the M step, on iteration *i* we find parameter 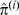 to maximize the expected complete-data log penalized likelihood:


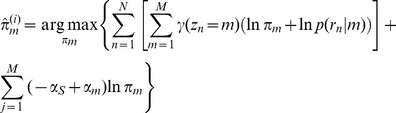


under the constraint 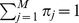. By simplifying we find the close-form solution of the maximization as:


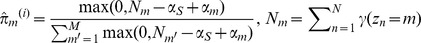


where *N_m_* is the effective number of reads assigned to event *m*, or the binding strength of event *m*. Intuitively, the effective read count of an event is decreased by a pseudo-count *α_s_* for the sparseness penalty, and is increased by a pseudo-count *α_m_* for the k-mer motif at position *m*. If for event *m*, the value of *π_m_* becomes zero, the model is restructured to eliminate it [46].

The EM algorithm is deemed to have converged when the change in likelihood falls below a small value, for example 1e−5.

Since the value of term −*α_s_*+*α_m_* is negative, a binding event supported by enriched k-mers may still be eliminated if it is not sufficiently supported by read data. In addition, a binding event not supported by enriched k-mers may still survive if it is sufficiently supported by the read data.

The predicted binding events are tested for significance as described previously [8]. Briefly, if a control dataset is available, we compare the number of reads in the ChIP event to the number of reads in the corresponding region in the control sample using a Binomial test. If control data is not available, we apply a statistical test that uses a dynamic Poisson distribution to account for local biases. To correct for multiple hypothesis testing, a Benjamini-Hochberg correction [48] is applied. It is worth mentioning that we only use read counts of events to test for significance.

The read spatial distribution of binding events is updated after each round of binding event prediction.

### Motif discovery using improved event locations

Phase 6 repeats Phase 2 and 3 motif discovery using the binding events predicted from Phase 5. As described in the results section (Figure 1), the spatial accuracy of binding events discovered from Phase 5 (GEM) is significantly improved from Phase 1 (GPS). Thus, these events will be more accurately centered on motifs and the performance of motif discovery is correspondingly improved.

### GEM software

GEM is a stand-alone Java software that takes alignment files of ChIP-Seq reads and a genome sequence as input and reports a list of predicted binding events and the explanatory binding motifs. It can be downloaded from our web site (http://cgs.csail.mit.edu/gem). For analysis with mammalian genomes, GEM requires about 5–15 G memory.

### Datasets

214 ENCODE ChIP-Seq datasets that have an embargo date before Oct 28, 2011 and have known motifs in public databases were downloaded from the ENCODE project website [18]. 16 mouse ES cell factor ChIP-Seq datasets published in references [16] and [28] were downloaded from GEO. ChIP-exo data were provided by Ho Sung Rhee and B. Franklin Pugh. FastQ files of the ChIP-Seq/ChIP-exo data were then aligned with genome (human hg19, mouse mm9) using Bowtie [49] version 0.12.7 with options “-q --best --strata -m 1 -p 4 --chunkmbs 1024”. The GABP ChIP-Seq data was downloaded from QuEST website (http://mendel.stanford.edu/SidowLab/downloads/quest/) and was pre-aligned to hg18 genome.

### Motif-finding performance metrics

GEM was applied to 214 ENCODE ChIP-Seq data. The motif PWMs output by GEM were collected. An alternate pipeline used the GPS peak-finder [8] to call binding events and used 7 different motif finding methods (AlignACE v4.0 [23], MDscan v2004 [22], MEME v4.7.0 [20], Weeder v1.4.2 [21], POSMO v2 [24], HMS v0.1 [13] and ChIPMunk v3 [14]) to discover motifs independently. For AlignACE, MDscan, MEME and Weeder, 100 bp sequences were extracted from the top 500 peaks from each dataset, as suggested by the MEME Suite's documentation based on the typical resolution of ChIP-Seq peaks. For POSMO, we extracted a set of 100 bp sequences from the top 500 GPS peaks. This set of sequences provided superior results when compared with sequences taken from the top 5000 1000 bp sequences (as suggested by the author of POSMO). For ChIP-Seq oriented methods, HMS and ChIPMunk, a set of 100 bp sequences and corresponding read coverage profiles were extracted from the top 500 GPS peaks. We found these conditions provided superior results than using sequences taken from the top 5000 200 bp sequences (as suggested by the authors of these methods). MEME was run with “-nmotifs 6” and Weeder was run with option “large”. POSMO was run with options “5000 11111111 sequence_file 1.6 2.5 20 200”. ChIPMunk was run with options “6 15 yes 1.0 p:read_coverage_profile 100 10 1 4 random 0.41”. HMS was run with options “-w motif_width -dna 4 -iteration 100 -chain 50 -seqprop 0.1 -strand 2 -base read_coverage_profile -dep 2”; motif_width was determined by width of motif discovered by MEME for the same data. All other parameters were the defaults specified by the authors.

We collected known binding preference motifs from the TRANSFAC [50], JASPAR [51], and Uniprobe [52] databases. We only include motifs of the factors of interest or motifs for the TF family but not motifs of factors in the same family because factors in the same family may have very different binding motifs. The list of database matrices is provided in Dataset S1. Discovered motifs were compared to known motifs using STAMP [19]. A motif with E-value less than 1e-5 was considered a match. For each program, we counted the number of datasets that had a motif matching at least one known motif of that transcription factor. In some cases, the correct motifs are not matched by the first motif that a method outputs, but by the second or later motifs. Therefore we compare the motif-finding performance using the top 1, top 2… or top 6 motifs. Little improvement is observed after the 6^th^ motifs.

### Evaluating spatial resolution of ChIP-Seq event calls

The genome-wide performance of spatial resolution in ChIP-Seq event calls is evaluated as following. We define effective spatial resolution as the average absolute value of the distance between genome coordinates of predicted binding events and the middle of the corresponding high-scoring binding motif hit. Because the center of the motif hit may not represent the true center of a binding event, the offsets to the motif were centered by subtracting the mean offsets. We compare spatial resolution on the “matched” set of predictions that are called by all the methods and correspond to the same high-scoring binding motif. Only those events within 100 bp of a motif match are included in the calculation. An alternative evaluation with all the events that have a motif at a distance less than 100 bp is also performed.

### Evaluating performance in deconvolving proximal binding events using GABP ChIP-Seq data

The genome-wide performance of proximal event discovery in ChIP-Seq data is evaluated as follows. For GABP dataset, we compared GEM against other 6 methods (GPS, SISSRs, MACS, cisGenome, Quest and PeakRanger) genome wide. We define a set of candidate sites that all have at least one event detected by all seven methods, and that contain two or more GABP motifs separated by less than 500 bp. We discovered 477 such sites. For each of these sites, we count the number of events discovered by different methods. GABP motif was retrieved from TRANSFAC database (M00341) [50]. A motif score threshold of 9.9, which is 60% of maximum PWM score, is used in this analysis.

### Analysis of ChIP-exo data

In this study, to test GEM's ability to automatically adapt to ChIP-exo data, we initialized GEM with a ChIP-Seq empirical read distribution, and ran GEM with one extra run (phase 5 and 6) so that GEM could use more accurately positioned events to refine the read distribution and use it for final prediction. In practice, the user can directly initialize GEM with a ChIP-exo empirical read distribution (provided with GEM software) and apply GEM the same way as analyzing ChIP-Seq data.

### Computing the pair wise transcription factor spatial relationships from binding calls

To study the *in vivo* binding spatial relationship between a pair of transcription factors A and B in the certain cell type and condition, we apply GEM independently to ChIP-Seq data from A and B to predict the respective binding sites. To compute the distribution of spacing between A relative to B, we compute the offsets of A binding sites from B binding sites within a 201 bp window. The sequence strand of the binding predictions is oriented using the B motif when a match to the motif is present, and B is placed in the middle of the window. The occurrences of A at each offset position are summed over all the B sites to produce the empirical spatial distribution. In this study, we evaluate three different methods to call binding sites: GEM binding calls, GPS binding calls, and GPS binding calls that are snapped to a motif within 50 bp if one is present. Another motif distance for snapping binding calls, 100 bp, was also tested and the result was very similar to the 50 bp distance.

To determine if a specific spacing is significant, we compute the p-value of the number of occurrences of factor A at that offset position using a Poisson test. The parameter of Poisson distribution is set as the mean number of occurrences across all the positions in the [−400 bp −200 bp] and [200 bp 400 bp] windows, assuming there are no significant spatial binding constraints in these windows. The p-value is corrected for multiple hypotheses testing using Bonferroni correction by multiplying the p-value by the number of positions in the window and the total number of pair wise tests across all cell types. The significance threshold for corrected p-value is 1e−8. Because the strand orientation of bound sequences cannot be oriented consistently when comparing multiple factor pairs, we report the absolute distance between the most significant interacting factor pairs in Figure 6.

## Supporting Information

Dataset S1 Public database motif matrices used in this study.
(TXT)

Dataset S2 GEM discovered primary and secondary motif matrices from ENCODE data.
(TXT)

Dataset S3 GEM discovered primary and secondary motif matrices from mouse ES cell data.
(TXT)

Figure S1 Spatial accuracy evaluation using all the binding events.
(PDF)

Figure S2 GEM improves the spatial resolution of Reb1 ChIP-exo data event prediction.
(PDF)

Figure S3 Color chart representation of 100 bp sequences in the regions with 6 bp Sox2/Oct4 binding constraint.
(PDF)

Figure S4 Spatial binding constraints detected from mouse ES cells.
(PDF)

Figure S5 Spatial relationship between Klf4 and other 15 factors in mouse ES cells.
(PDF)

Figure S6 Sox2/Klf4/Esrrb/Nr5a2/Tcfcp2l1 bound regions are bound by p300 and marked by H3K27ac.
(PDF)

Figure S7 Spatial binding constraints detected from ENCODE GM12878 cells.
(PDF)

Figure S8 Spatial binding constraints detected from ENCODE HepG2 cells.
(PDF)

Figure S9 Spatial binding constraints detected from ENCODE HeLa-S3 cells.
(PDF)

Figure S10 Spatial binding constraints detected from ENCODE H1 cells.
(PDF)

Figure S11 Color chart representation of 100 bp sequences in 259 regions with 4 bp c-Fos:c-Jun/USF1 binding constraint.
(PDF)

Figure S12 Color chart representation of 100 bp sequences in 315 regions with 4 bp CTCF/Egr1 binding constraint.
(PDF)

Figure S13 Color chart representation of 100 bp sequences in 4215 regions with a wide range of HNF4A/FOXA1 binding constraints.
(PDF)

Figure S14 K-mer class motif clustering.
(PDF)

Table S1 Known motifs recovered by GEM in ENCODE data.
(PDF)

Table S2 Performance of motif discovery methods by individual ENCODE ChIP-Seq experiments.
(PDF)

Table S3 Overall performance of motif discovery methods for ENCODE data.
(PDF)

Table S4 Novel motifs discovered by GEM in ENCODE data.
(PDF)

Table S5 Motifs of mouse ES cell factors discovered by GEM.
(PDF)

Table S6 All significant pairwise spatial binding constraints detected from mouse ES cell ChIP-Seq data.
(PDF)

Table S7 Non-redundant significant pairwise spatial binding constraints detected from mouse ES cell ChIP-Seq data, consolidated to non-redundant factor pairs.
(PDF)

Table S8 All significant pairwise spatial binding constraints detected from ENCODE (5 cell types) ChIP-Seq data.
(TXT)

Table S9 Non-redundant significant pairwise spatial binding constraints detected from ENCODE (5 cell types) ChIP-Seq data.
(TXT)
