# Supplementary material for: High Resolution Genome Wide Binding Event Finding and Motif Discovery Reveals Transcription Factor Spatial Binding Constraints
Source: PLoS Comput Biol. 2012 Aug 9;8(8):e1002638. doi: 10.1371/journal.pcbi.1002638 (PMC3415389; doi:10.1371/journal.pcbi.1002638)

Figure S1 Spatial accuracy evaluation using all the binding events

A) Fraction of predicted GABP binding events with a motif within the given distance with event discovery by GEM, GPS, SISSRs, MACS, cisGenome, QuEST and PeakRanger. Events shown were all the events predicted by each of the seven methods and had a GABP motif within 100bp.

B) Fraction of predicted CTCF binding events with a motif within the given distance with event discovery by GEM, GPS, SISSRs, MACS, cisGenome, QuEST, FindPeaks, spp-wtd and spp-mtc. Events shown were all the events predicted by each of the nine methods and had a CTCF motif within 100bp.

A

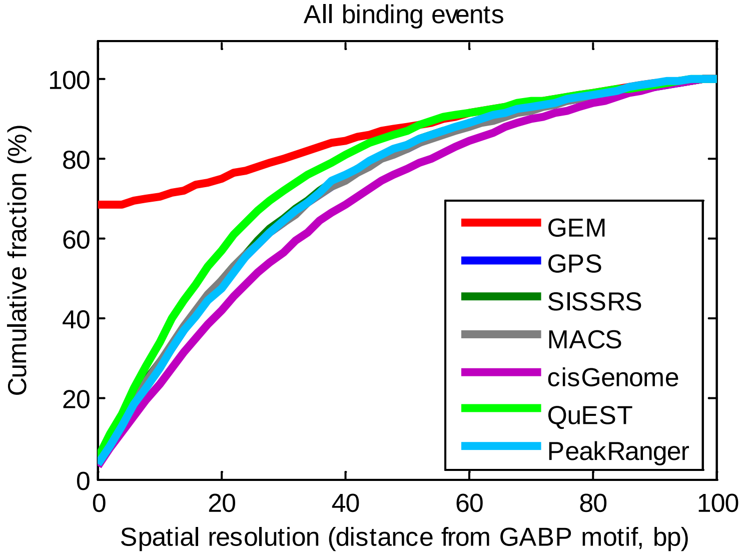

B

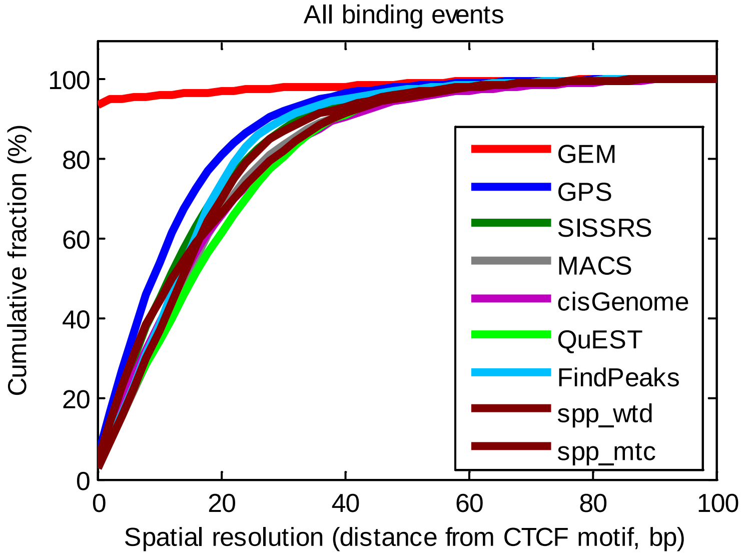

Supplement: Figure S1 — Spatial accuracy evaluation using all the binding events. (PDF) [file pcbi.1002638.s004.pdf]
