# Supplementary material for: High Resolution Genome Wide Binding Event Finding and Motif Discovery Reveals Transcription Factor Spatial Binding Constraints
Source: PLoS Comput Biol. 2012 Aug 9;8(8):e1002638. doi: 10.1371/journal.pcbi.1002638 (PMC3415389; doi:10.1371/journal.pcbi.1002638)

Figure S2 GEM improves the spatial resolution of Reb1 ChIP-exo data event prediction.
A) Fraction of predicted Reb1 binding events with a motif within the given distance with event discovery by GEM, GPS, and the peak-pair midpoint method of Rhee, et al. GEM and GPS are initialized with a ChIP-Seq read distribution. **B**) GEM automatically adapts to the Reb1 ChIP-exo read spatial distribution.

A

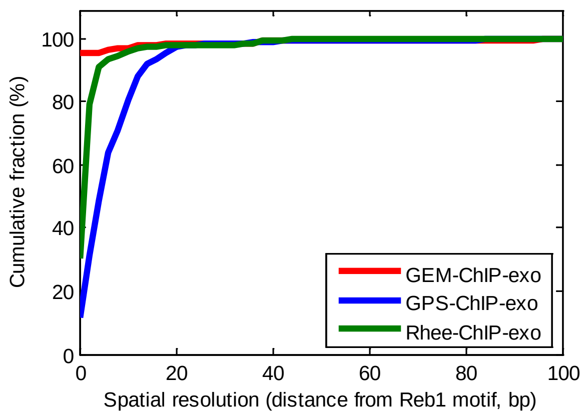

B

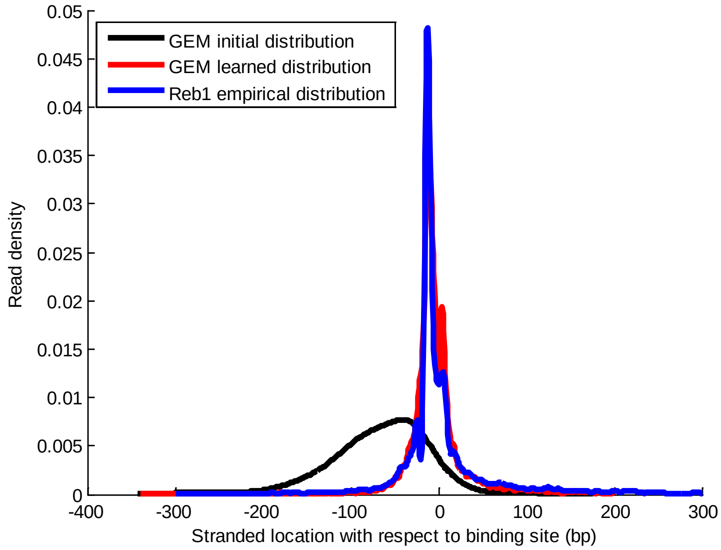

Supplement: Figure S2 — GEM improves the spatial resolution of Reb1 ChIP-exo data event prediction. (PDF) [file pcbi.1002638.s005.pdf]
