# Supplementary material for: High Resolution Genome Wide Binding Event Finding and Motif Discovery Reveals Transcription Factor Spatial Binding Constraints
Source: PLoS Comput Biol. 2012 Aug 9;8(8):e1002638. doi: 10.1371/journal.pcbi.1002638 (PMC3415389; doi:10.1371/journal.pcbi.1002638)

Figure S3 Color chart representation of 100bp sequences in the regions with 6bp Sox2/Oct4 binding constraint

Each row represents a 100bp bound sequence. Green, blue, yellow and red indicate A, C, G and T. The motif logos are generated by STAMP [19] from the motifs discovered using all the binding sites in the respective datasets.

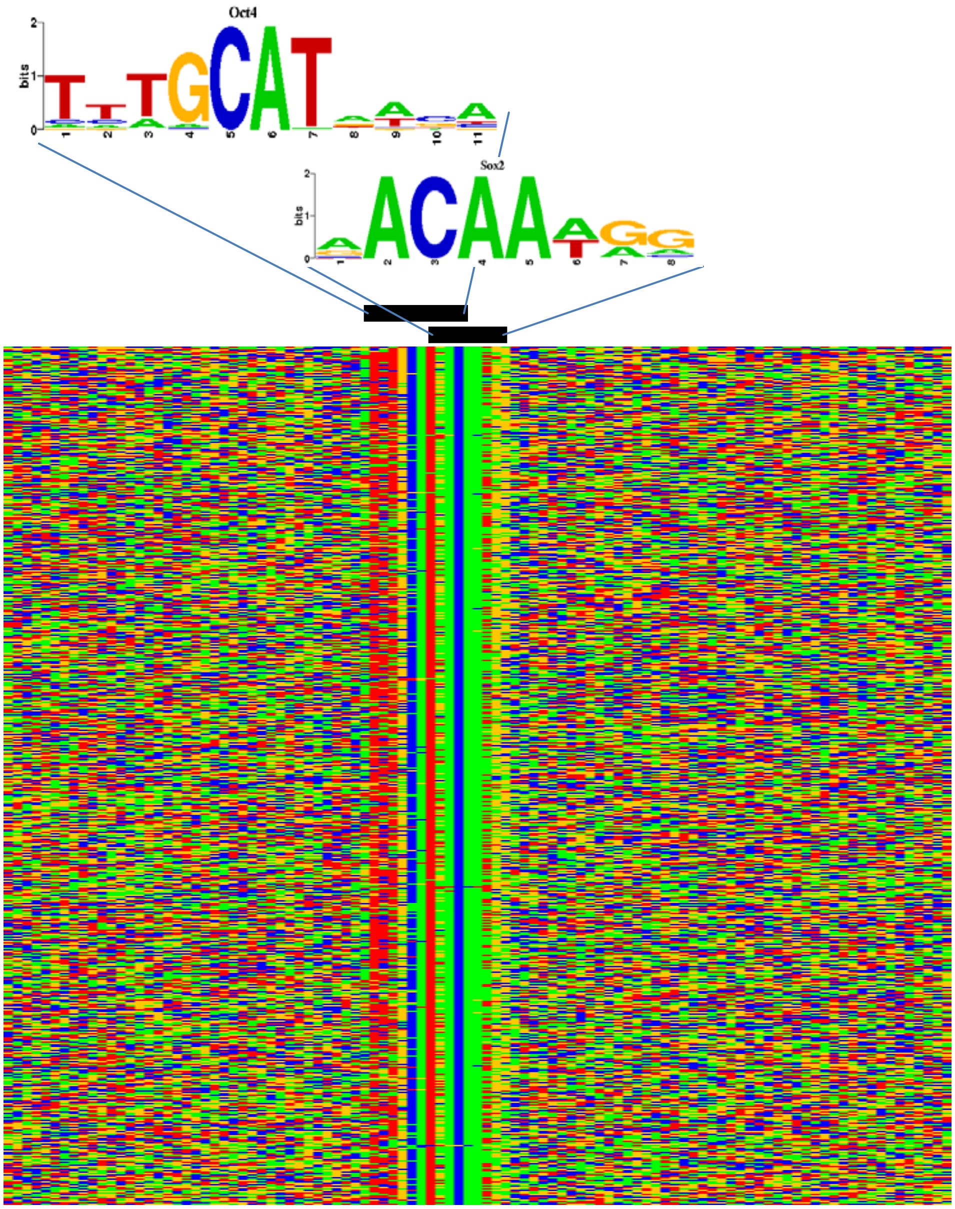

Supplement: Figure S3 — Color chart representation of 100 bp sequences in the regions with 6 bp Sox2/Oct4 binding constraint. (PDF) [file pcbi.1002638.s006.pdf]
