# Supplementary material for: High Resolution Genome Wide Binding Event Finding and Motif Discovery Reveals Transcription Factor Spatial Binding Constraints
Source: PLoS Comput Biol. 2012 Aug 9;8(8):e1002638. doi: 10.1371/journal.pcbi.1002638 (PMC3415389; doi:10.1371/journal.pcbi.1002638)
Supplement: Figure S4 — Spatial binding constraints detected from mouse ES cells. (PDF) [file pcbi.1002638.s007.pdf]

Figure S4 Spatial binding constraints detected from mouse ES cells.
A) Matrix representation of pairwise spatial binding constraints between factor B (column) and factor A (row) detected from 16 ChIP-Seq dataset in mouse ES cells. The colors represent the significance levels (corrected p-value) of the strongest spacings. The numbers represent the distances between the factors in the strongest spacing. **B**) The colors and numbers represent the number of positions exhibiting significant spatial binding constraints within the 201bp window around the binding sites of factor B (column).

A

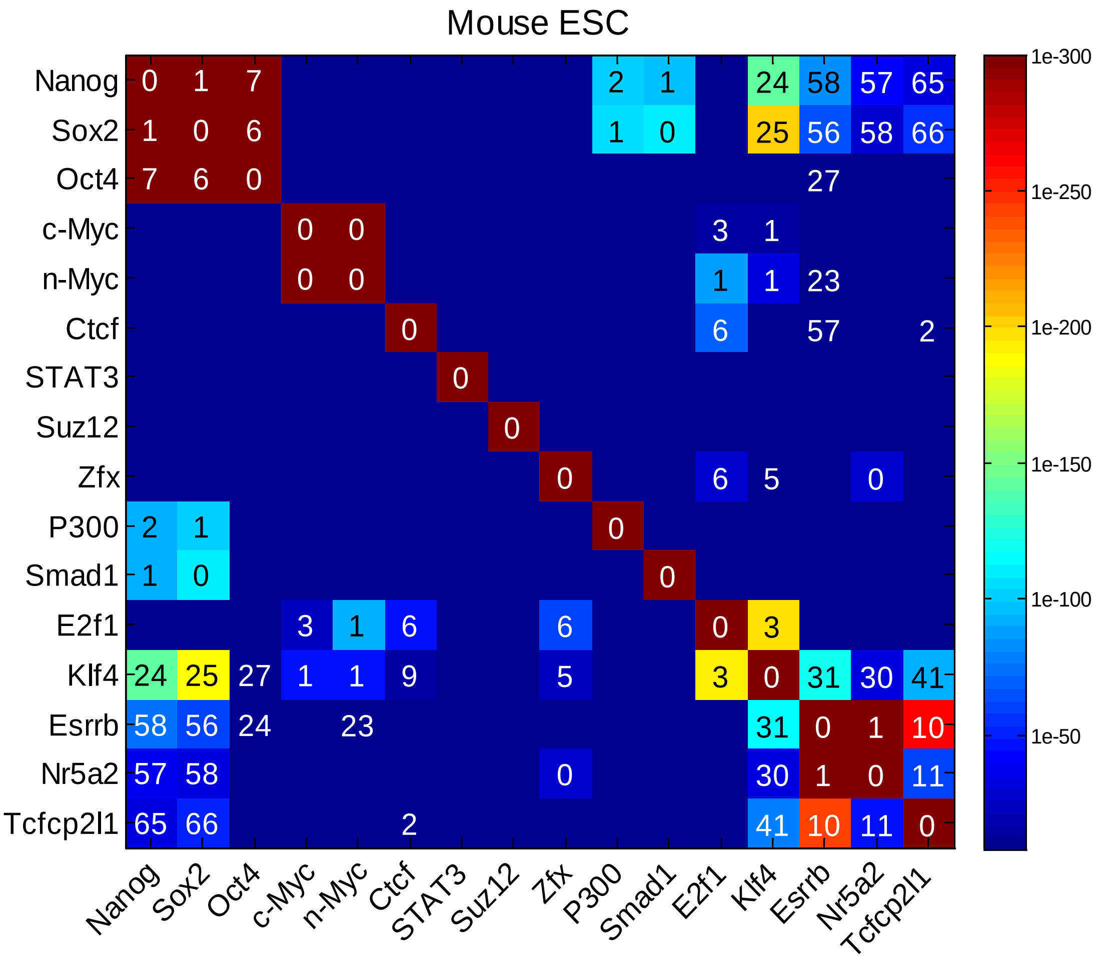

B

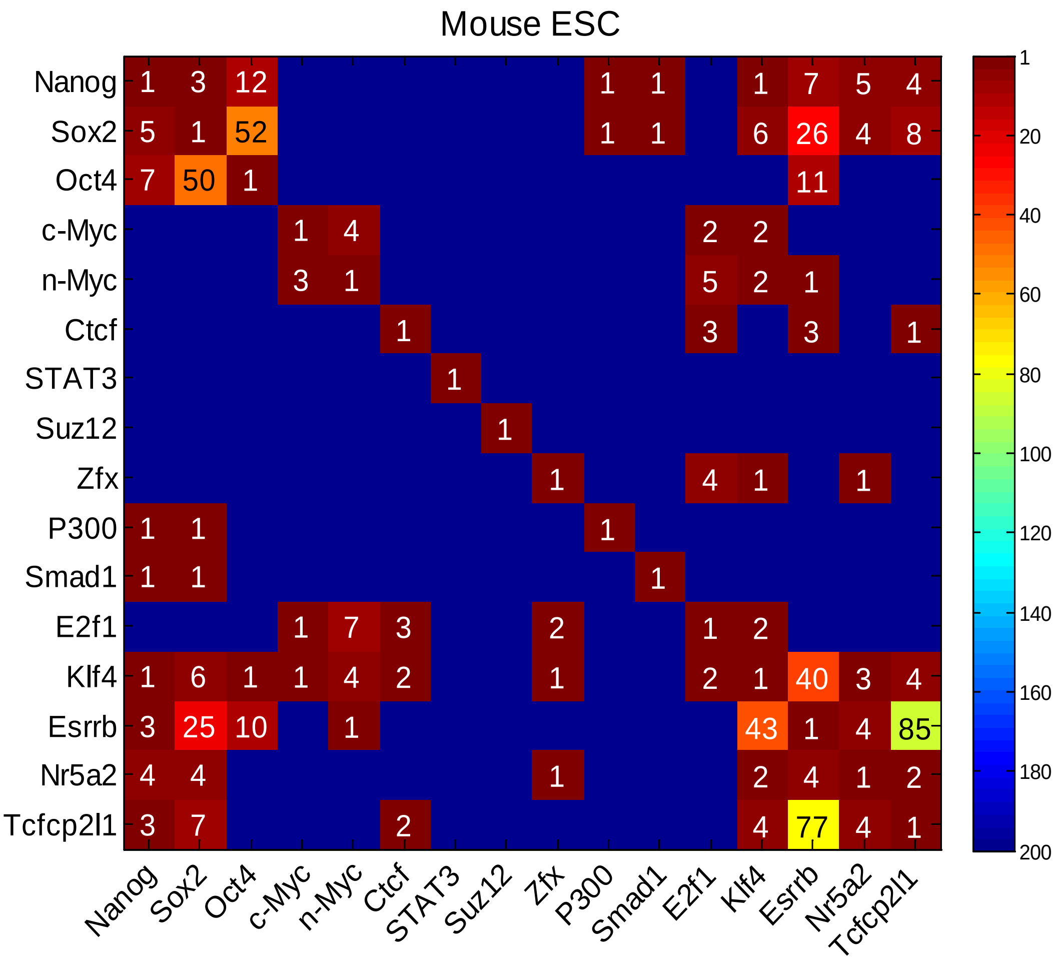
Mouse ESC
Nanog
Sox2
Oct4
c-Myc
n-Myc
Ctcf
STAT3
Suz12
Zfx
P300
Smad1
E2f1
Klf4
Esrrb
Nr5a2
Tcfcp2l1
1
20
40
60
80
100
120
140
160
180
200
