# Supplementary material for: High Resolution Genome Wide Binding Event Finding and Motif Discovery Reveals Transcription Factor Spatial Binding Constraints
Source: PLoS Comput Biol. 2012 Aug 9;8(8):e1002638. doi: 10.1371/journal.pcbi.1002638 (PMC3415389; doi:10.1371/journal.pcbi.1002638)

Figure S5 Spatial relationship between Klf4 and other 15 factors in mouse ES cells

Spatial distribution of 16 mouse ES cell factor binding sites in a 201bp window around Klf4 binding sites. Vertical dash-dot lines represent the Klf4 binding sites at position 0; horizontal dashed lines represent the number of occurrences at a position corresponding to corrected p-value of 1e-8.

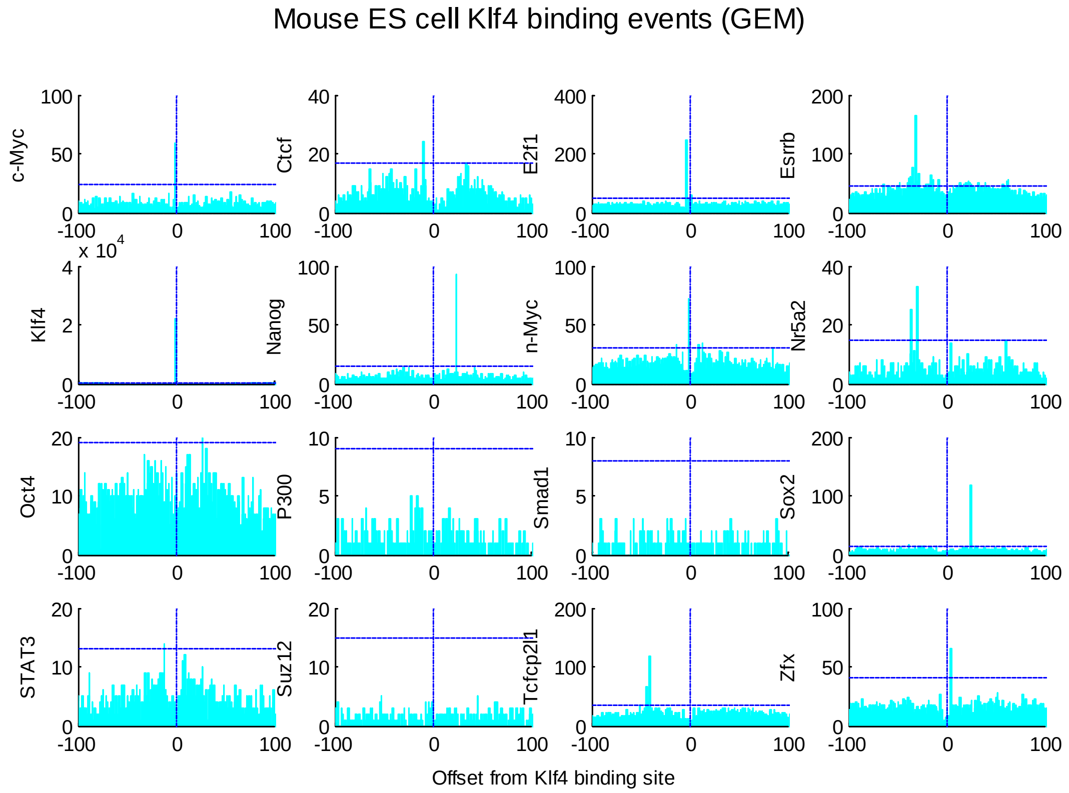

Supplement: Figure S5 — Spatial relationship between Klf4 and other 15 factors in mouse ES cells. (PDF) [file pcbi.1002638.s008.pdf]
