# Supplementary material for: High Resolution Genome Wide Binding Event Finding and Motif Discovery Reveals Transcription Factor Spatial Binding Constraints
Source: PLoS Comput Biol. 2012 Aug 9;8(8):e1002638. doi: 10.1371/journal.pcbi.1002638 (PMC3415389; doi:10.1371/journal.pcbi.1002638)

Figure S6 Sox2/Klf4/Esrrb/Nr5a2/Tcfcp2l1 bound regions are bound by p300 and marked by H3K27ac

Read profiles and Heatmaps of 123 Sox2/Klf4/Esrrb/Nr5a2/Tcfcp2l1 bound regions show p300 read enrichment and K3K27ac mark read enrichment. Top: read profile, bottom: Heatmap of read coverage. The regions are 2kbp over the Sox2 binding sites. The regions are in same order as in Figure 5. Color shading corresponds to the ChIP-Seq read count in the region.

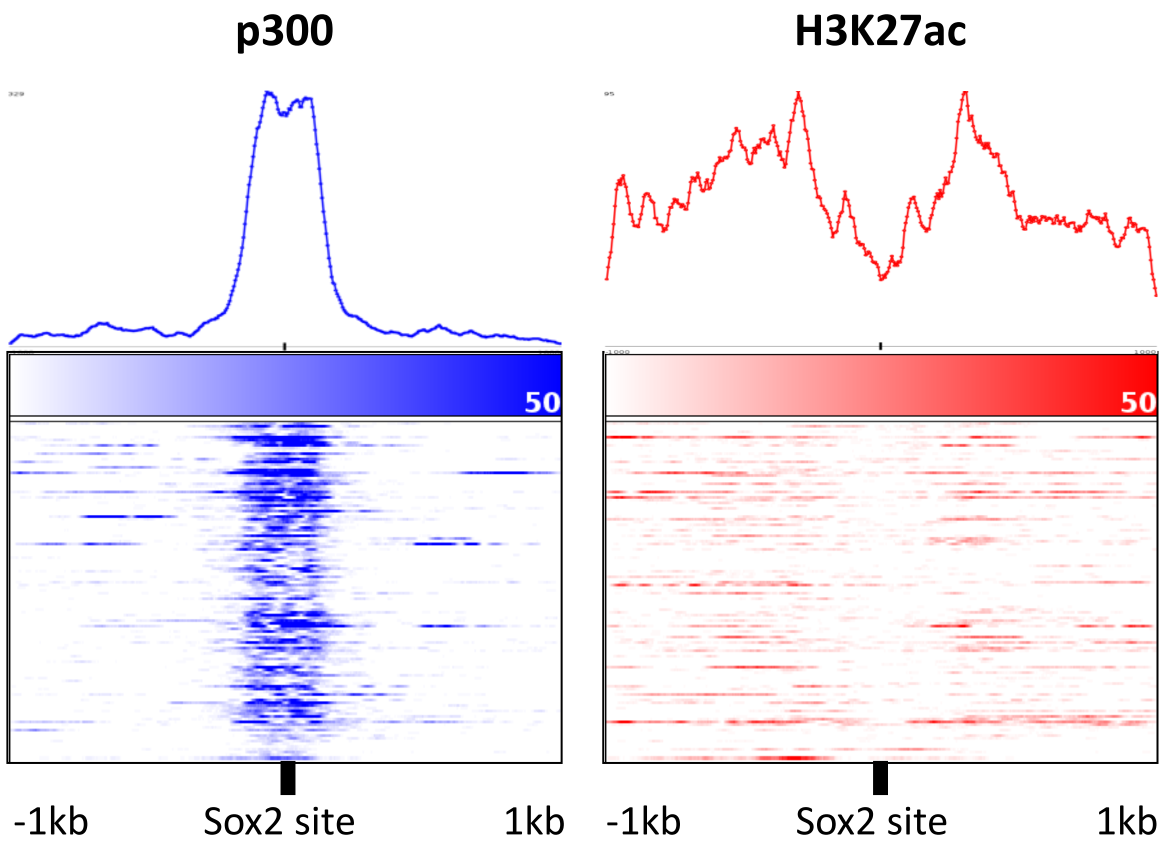

Supplement: Figure S6 — Sox2/Klf4/Esrrb/Nr5a2/Tcfcp2l1 bound regions are bound by p300 and marked by H3K27ac. (PDF) [file pcbi.1002638.s009.pdf]
