# Supplementary material for: High Resolution Genome Wide Binding Event Finding and Motif Discovery Reveals Transcription Factor Spatial Binding Constraints
Source: PLoS Comput Biol. 2012 Aug 9;8(8):e1002638. doi: 10.1371/journal.pcbi.1002638 (PMC3415389; doi:10.1371/journal.pcbi.1002638)
Supplement: Figure S7 — Spatial binding constraints detected from ENCODE GM12878 cells. (PDF) [file pcbi.1002638.s010.pdf]

Figure S7 Spatial binding constraints detected from ENCODE GM12878 cells.
A) Matrix representation of pairwise spatial binding constraints between factor B (column) and factor A (row) detected from 29 ChIP-Seq dataset in human GM12878 cells. The colors represent the significance levels (corrected p-value) of the strongest spacings. The numbers represent the distances between the factors in the strongest spacings. **B**) The colors and numbers represent the number of positions exhibiting significant spatial binding constraints within the 201bp window around the binding sites of factor B (column).

A

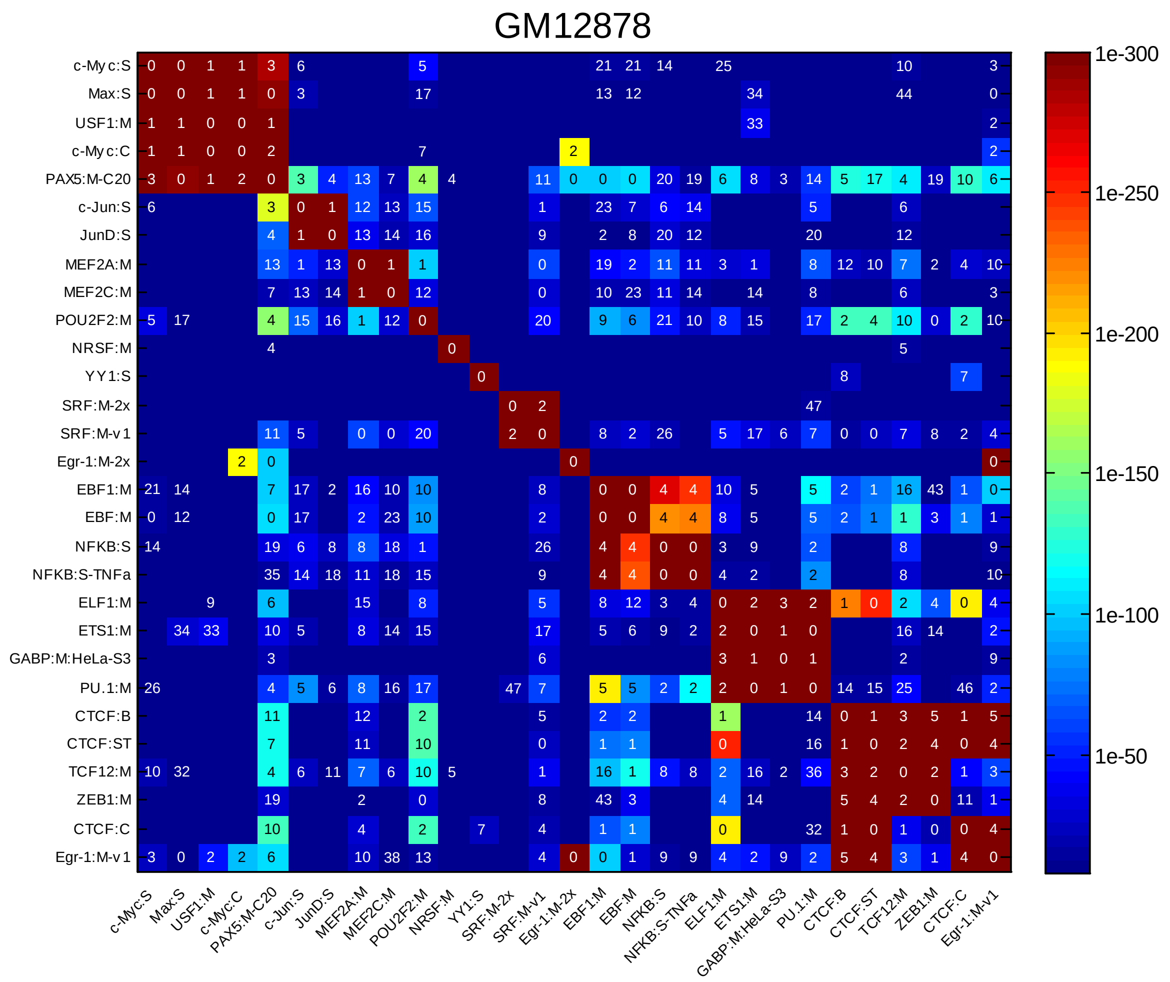

B

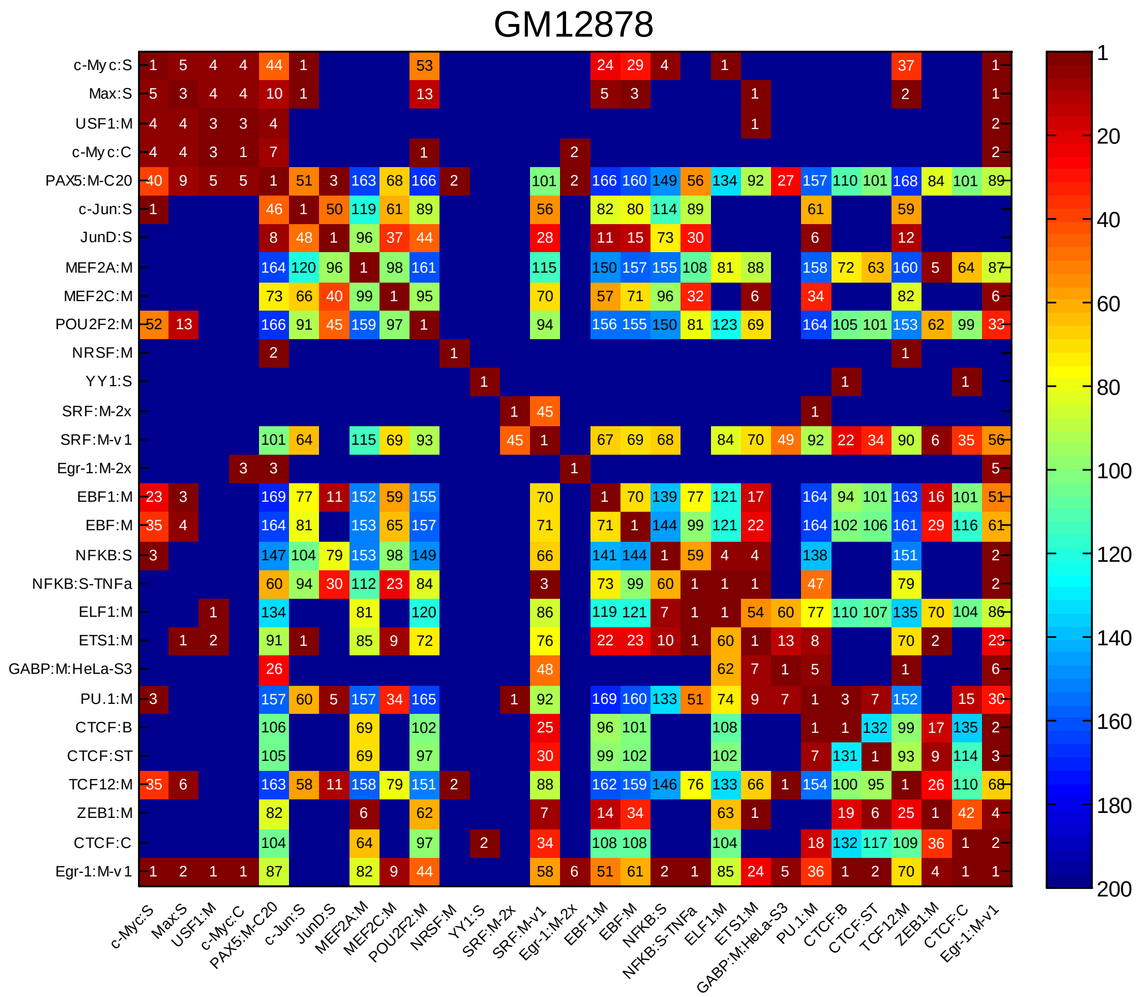
GM12878
c-Myc:S
Max:S
USF1:M
c-Myc:C
PAX5:M-C20
c-Jun:S
JunD:S
MEF2A:M
MEF2C:M
POU2F2:M
NRSF:M
YY1:S
SRF:M-2x
SRF:M-v1
Egr-1:M-2x
EBF1:M
EBF:M
NFKB:S
NFKB:S-TNFa
ELF1:M
ETS1:M
GABP:M:HeLa-S3
PU.1:M
CTCF:B
CTCF:ST
TCF12:M
ZEB1:M
CTCF:C
Egr-1:M-v1
1
20
40
60
80
100
120
140
160
180
200
