# Supplementary material for: High Resolution Genome Wide Binding Event Finding and Motif Discovery Reveals Transcription Factor Spatial Binding Constraints
Source: PLoS Comput Biol. 2012 Aug 9;8(8):e1002638. doi: 10.1371/journal.pcbi.1002638 (PMC3415389; doi:10.1371/journal.pcbi.1002638)
Supplement: Figure S8 — Spatial binding constraints detected from ENCODE HepG2 cells. (PDF) [file pcbi.1002638.s011.pdf]

Figure S8 Spatial binding constraints detected from ENCODE HepG2 cells.
A) Matrix representation of pairwise spatial binding constraints between factor B (column) and factor A (row) detected from 29 ChIP-Seq dataset in human HepG2 cells. The colors represent the significance levels (corrected p-value) of the strongest spacings. The numbers represent the distances between the factors in the strongest spacings. **B**) The colors and numbers represent the number of positions exhibiting significant spatial binding constraints within the 201bp window around the binding sites of factor B (column).

A

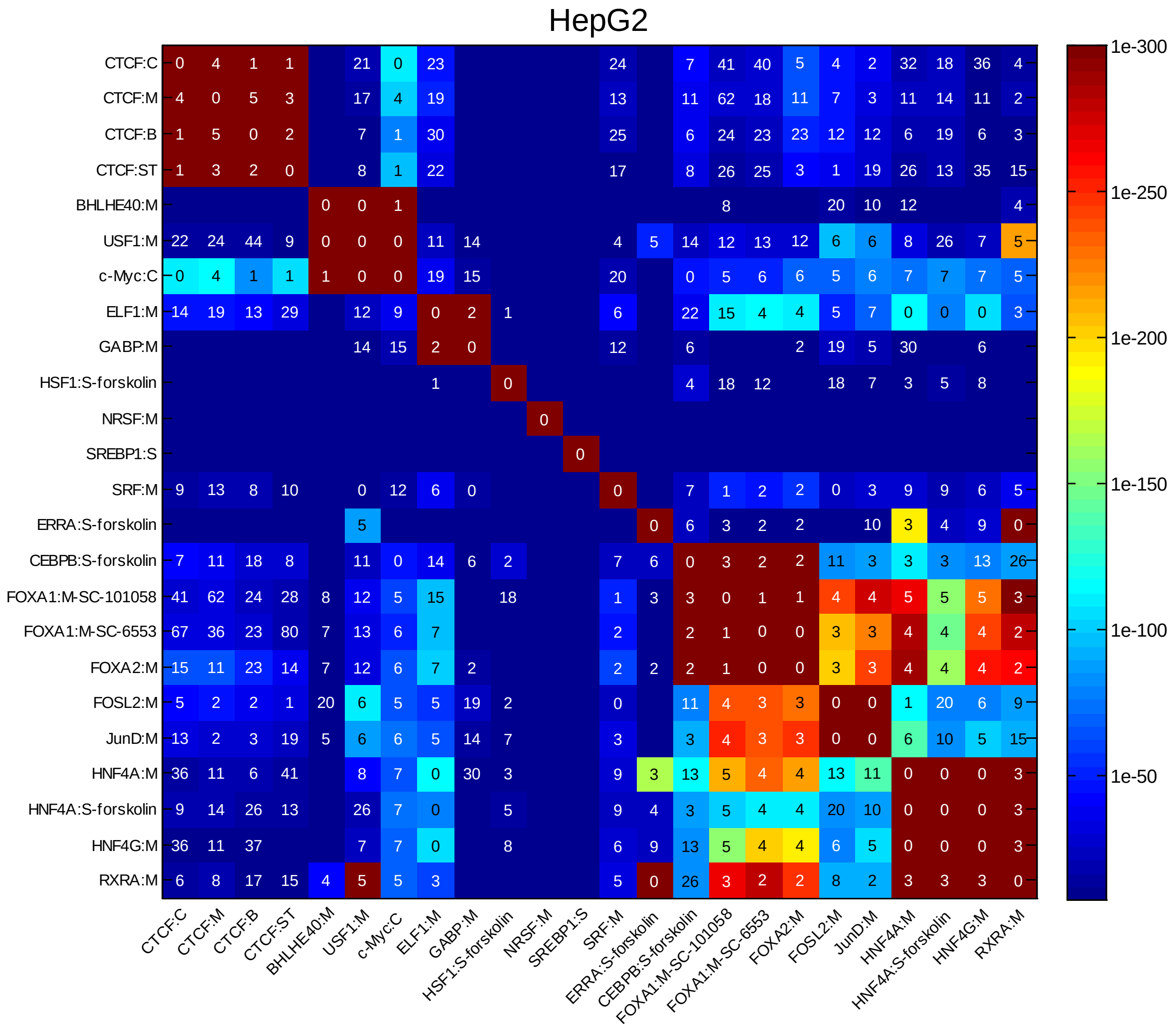

B

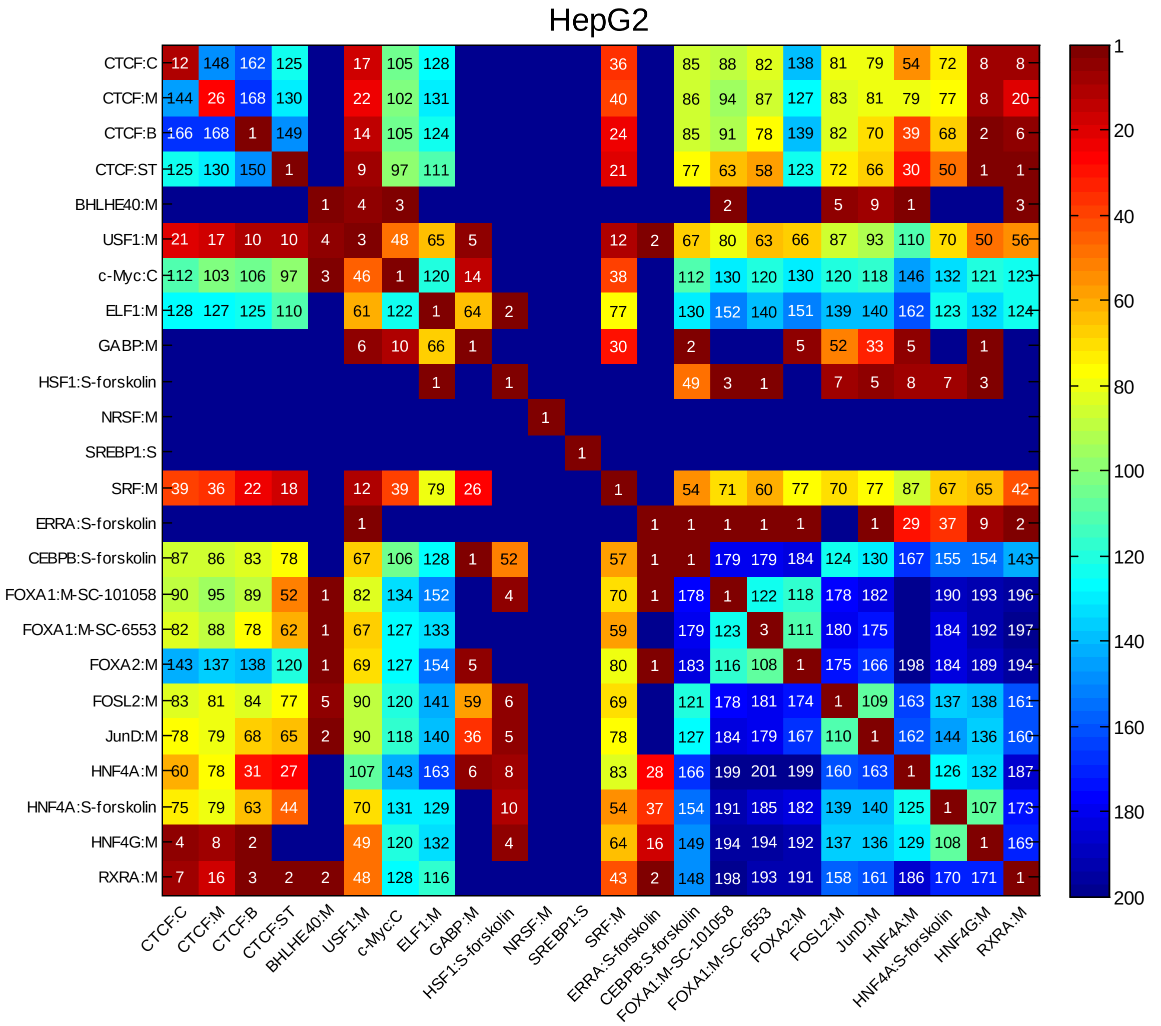
HepG2
CTCF:C 12 148 162 125 17 105 128 36 85 88 82 138 81 79 54 72 8 8
CTCF:M 144 26 168 130 22 102 131 40 86 94 87 127 83 81 79 77 8 20
CTCF:B 166 168 1 149 14 105 124 24 85 91 78 139 82 70 39 68 2 6
CTCF:ST 125 130 150 1 9 97 111 21 77 63 58 123 72 66 30 50 1 1
BHLHE40:M 1 4 3 2 5 9 1 3
USF1:M 21 17 10 10 4 3 48 65 5 12 2 67 80 63 66 87 93 110 70 50 56
c-Myc:C 112 103 106 97 3 46 1 120 14 38 112 130 120 130 120 118 146 132 121 123
ELF1:M 128 127 125 110 61 122 1 64 2 77 130 152 140 151 139 140 162 123 132 124
GABP:M 6 10 66 1 30 2 5 52 33 5 1
HSF1:S-forskolin 1 1 49 3 1 7 5 8 7 3
NRSF:M 1
SREBP1:S 1
SRF:M 39 36 22 18 12 39 79 26 1 54 71 60 77 70 77 87 67 65 42
ERRA:S-forskolin 1 1 1 1 1 1 1 29 37 9 2
CEBPB:S-forskolin 87 86 83 78 67 106 128 1 52 57 1 1 179 179 184 124 130 167 155 154 143
FOXA1:M-SC-101058 90 95 89 52 1 82 134 152 4 70 1 178 1 122 118 178 182 190 193 196
FOXA1:M-SC-6553 82 88 78 62 1 67 127 133 59 179 123 3 111 180 175 184 192 197
FOXA2:M 143 137 138 120 1 69 127 154 5 80 1 183 116 108 1 175 166 198 184 189 194
FOSL2:M 83 81 84 77 5 90 120 141 59 6 69 121 178 181 174 1 109 163 137 138 161
JunD:M 78 79 68 65 2 90 118 140 36 5 78 127 184 179 167 110 1 162 144 136 160
HNF4A:M 60 78 31 27 107 143 163 6 8 83 28 166 199 201 199 160 163 1 126 132 187
HNF4A:S-forskolin 75 79 63 44 70 131 129 10 54 37 154 191 185 182 139 140 125 1 107 173
HNF4G:M 4 8 2 49 120 132 4 64 16 149 194 194 192 137 136 129 108 1 169
RXRA:M 7 16 3 2 2 48 128 116 43 2 148 198 193 191 158 161 186 170 171 1
CTCF:C CTCF:M CTCF:B CTCF:ST BHLHE40:M USF1:M c-Myc:C ELF1:M GABP:M HSF1:S-forskolin NRSF:M SREBP1:S SRF:M ERRA:S-forskolin CEBPB:S-forskolin FOXA1:M-SC-101058 FOXA1:M-SC-6553 FOXA2:M FOSL2:M JunD:M HNF4A:M HNF4A:S-forskolin HNF4G:M RXRA:M
1
20
40
60
80
100
120
140
160
180
200
