# Supplementary material for: High Resolution Genome Wide Binding Event Finding and Motif Discovery Reveals Transcription Factor Spatial Binding Constraints
Source: PLoS Comput Biol. 2012 Aug 9;8(8):e1002638. doi: 10.1371/journal.pcbi.1002638 (PMC3415389; doi:10.1371/journal.pcbi.1002638)
Supplement: Figure S9 — Spatial binding constraints detected from ENCODE HeLa-S3 cells. (PDF) [file pcbi.1002638.s012.pdf]

Figure S9 Spatial binding constraints detected from ENCODE HeLa-S3 cells.
A) Matrix representation of pairwise spatial binding constraints between factor B (column) and factor A (row) detected from 15 ChIP-Seq dataset in human HeLa-S3 cells. The colors represent the significance levels (corrected p-value) of the strongest spacings. The numbers represent the distances between the factors in the strongest spacings. **B**) The colors and numbers represent the number of positions exhibiting significant spatial binding constraints within the 201bp window around the binding sites of factor B (column).

A

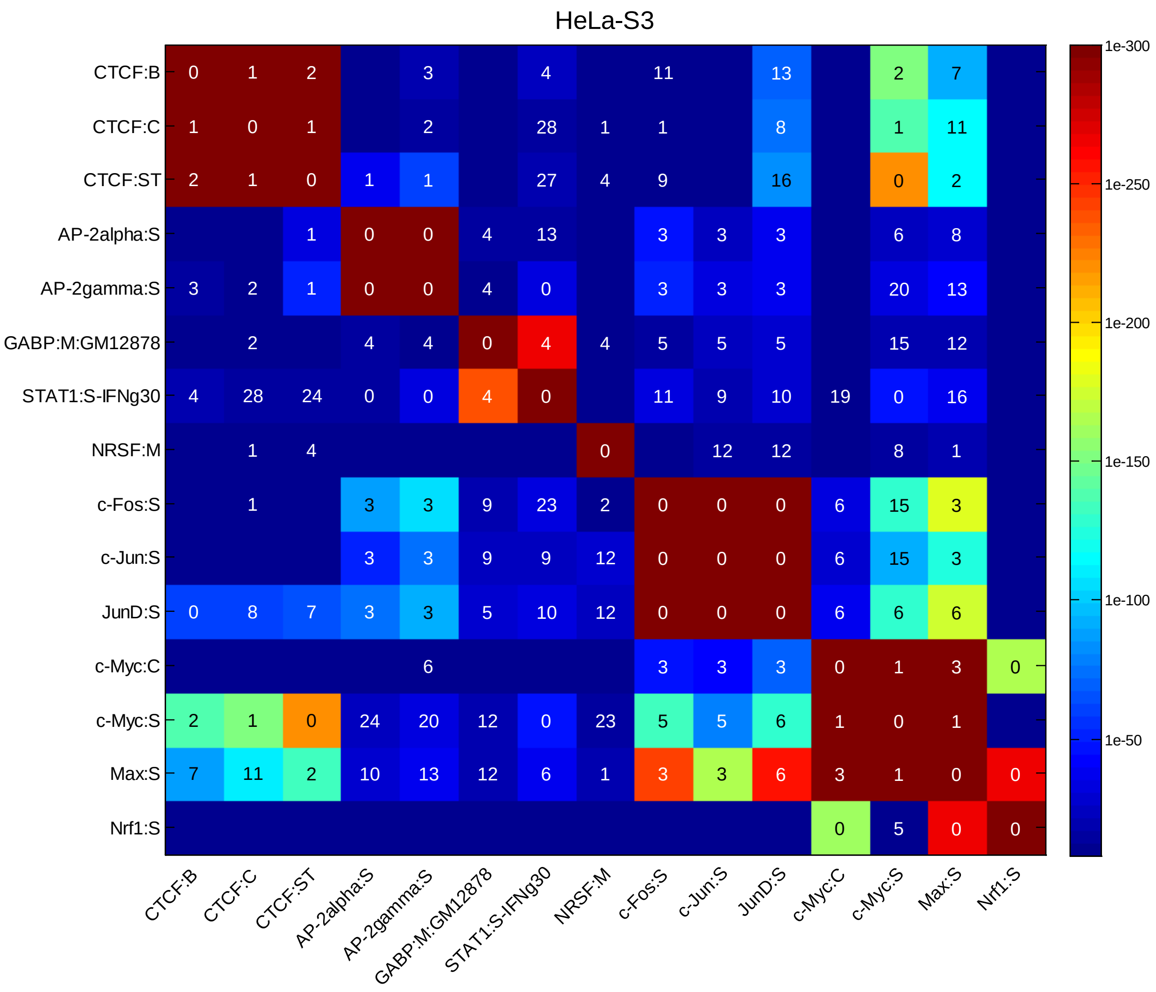

B

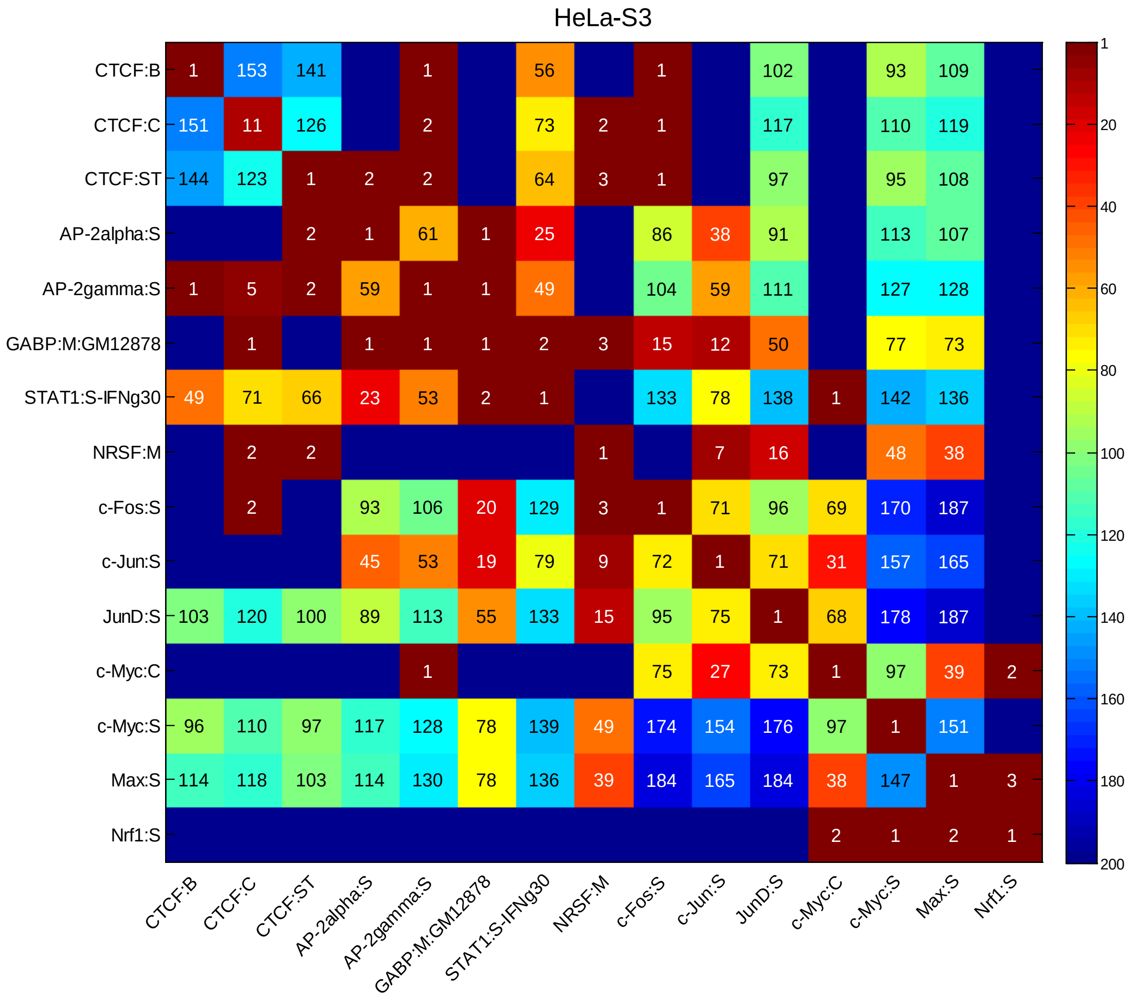
HeLa-S3
CTCF:B
CTCF:C
CTCF:ST
AP-2alpha:S
AP-2gamma:S
GABP:M:GM12878
STAT1:S-IFNg30
NRSF:M
c-Fos:S
c-Jun:S
JunD:S
c-Myc:C
c-Myc:S
Max:S
Nrf1:S
1
20
40
60
80
100
120
140
160
180
200
