# Supplementary material for: High Resolution Genome Wide Binding Event Finding and Motif Discovery Reveals Transcription Factor Spatial Binding Constraints
Source: PLoS Comput Biol. 2012 Aug 9;8(8):e1002638. doi: 10.1371/journal.pcbi.1002638 (PMC3415389; doi:10.1371/journal.pcbi.1002638)
Supplement: Figure S10 — Spatial binding constraints detected from ENCODE H1 cells. (PDF) [file pcbi.1002638.s013.pdf]

Figure S10 Spatial binding constraints detected from ENCODE H1 cells.
A) Matrix representation of pairwise spatial binding constraints between factor B (column) and factor A (row) detected from 11 ChIP-Seq dataset in human H1 cells. The colors represent the significance levels (corrected p-value) of the strongest spacings. The numbers represent the distances between the factors in the strongest spacings. **B**) The colors and numbers represent the number of positions exhibiting significant spatial binding constraints within the 201bp window around the binding sites of factor B (column).

A

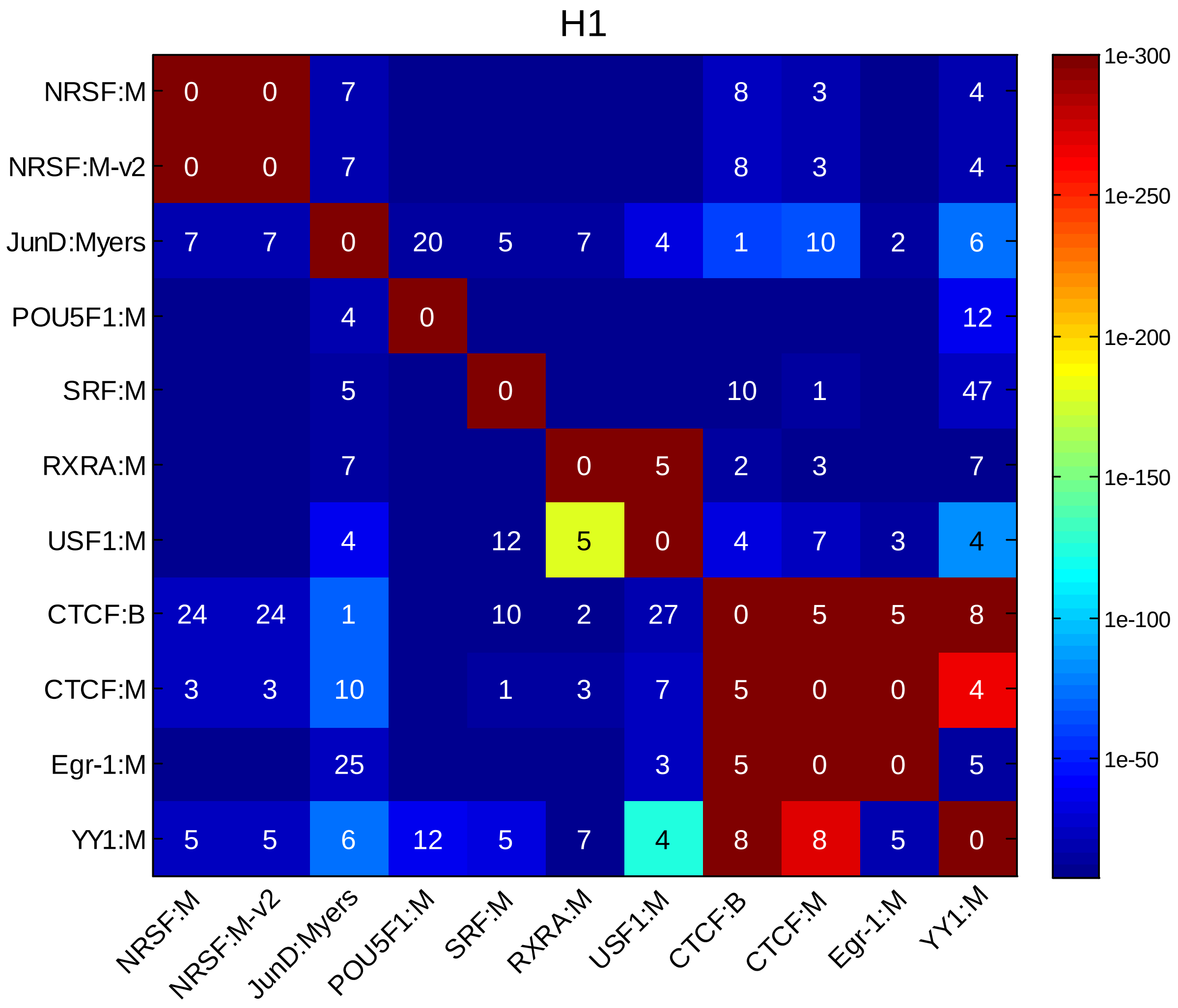

B

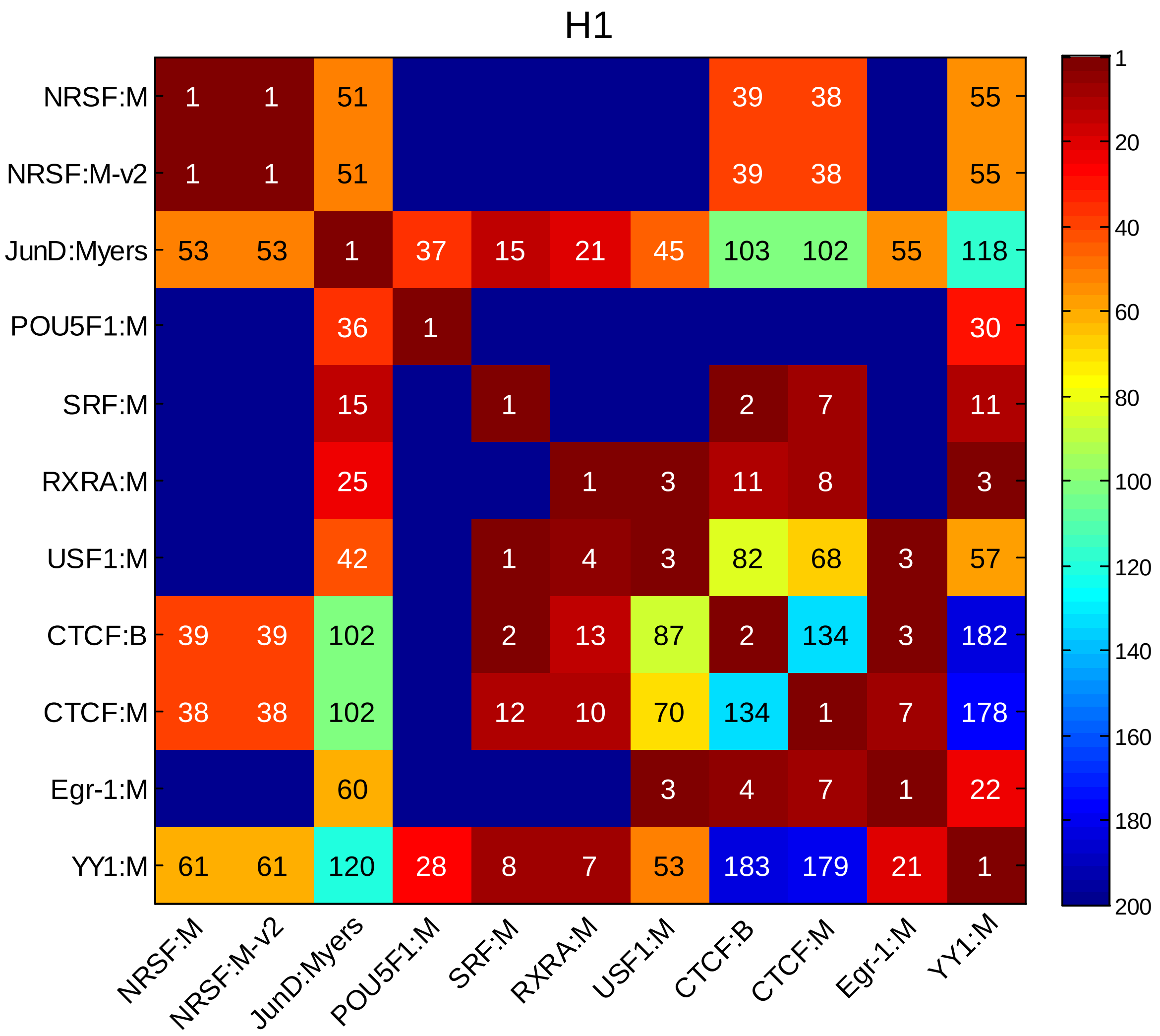
H1
NRSF:M
NRSF:M-v2
JunD:Myers
POU5F1:M
SRF:M
RXRA:M
USF1:M
CTCF:B
CTCF:M
Egr-1:M
YY1:M
1 1 51 39 38 55
1 1 51 39 38 55
53 53 1 37 15 21 45 103 102 55 118
36 1 30
15 1 2 7 11
25 1 3 11 8 3
42 1 4 3 82 68 3 57
39 39 102 2 13 87 2 134 3 182
38 38 102 12 10 70 134 1 7 178
60 3 4 7 1 22
61 61 120 28 8 7 53 183 179 21 1
1
20
40
60
80
100
120
140
160
180
200
