# Supplementary material for: High Resolution Genome Wide Binding Event Finding and Motif Discovery Reveals Transcription Factor Spatial Binding Constraints
Source: PLoS Comput Biol. 2012 Aug 9;8(8):e1002638. doi: 10.1371/journal.pcbi.1002638 (PMC3415389; doi:10.1371/journal.pcbi.1002638)

Figure S13 Color chart representation of 100bp sequences in 4215 regions with a wide range of HNF4A/FOXA1 binding constraints

Each row represents a 100bp bound sequence. Green, blue, yellow and red indicate A, C, G and T. The motif logos are generated by STAMP [19] from the motifs discovered using all the binding sites in the respective datasets.

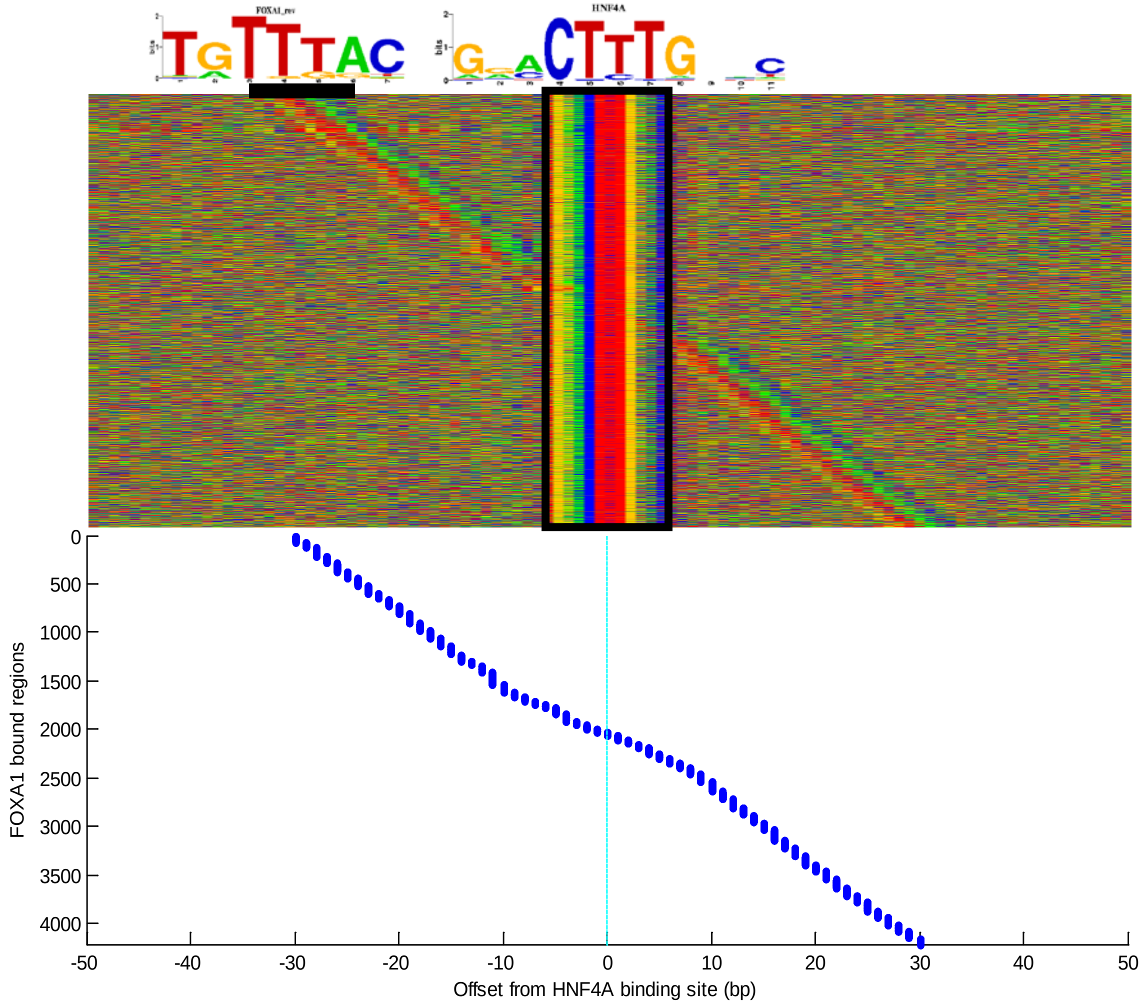

Supplement: Figure S13 — Color chart representation of 100 bp sequences in 4215 regions with a wide range of HNF4A/FOXA1 binding constraints. (PDF) [file pcbi.1002638.s016.pdf]
