# Supplementary material for: High Resolution Genome Wide Binding Event Finding and Motif Discovery Reveals Transcription Factor Spatial Binding Constraints
Source: PLoS Comput Biol. 2012 Aug 9;8(8):e1002638. doi: 10.1371/journal.pcbi.1002638 (PMC3415389; doi:10.1371/journal.pcbi.1002638)

Figure S14 K-mer class motif clustering

A) Examples of top ranking Oct4 k-mers. **B**) Schematic of k-mer equivalence class clustering. The numbers label the steps as described in the Method section.

A

K-mer	Offset	Pos Hit	Neg Hit
-----ATGCAAAT	-3	739	30
----TATGCAAA	-4	628	33
------TGCAAATG	-2	460	22
-----ATGCTAAT	-3	382	12
---TTATGCAA	-5	358	13
-----ATGCATAT	-3	320	21
------TGCAAATT	-2	222	18
... ...	...	...	...

B

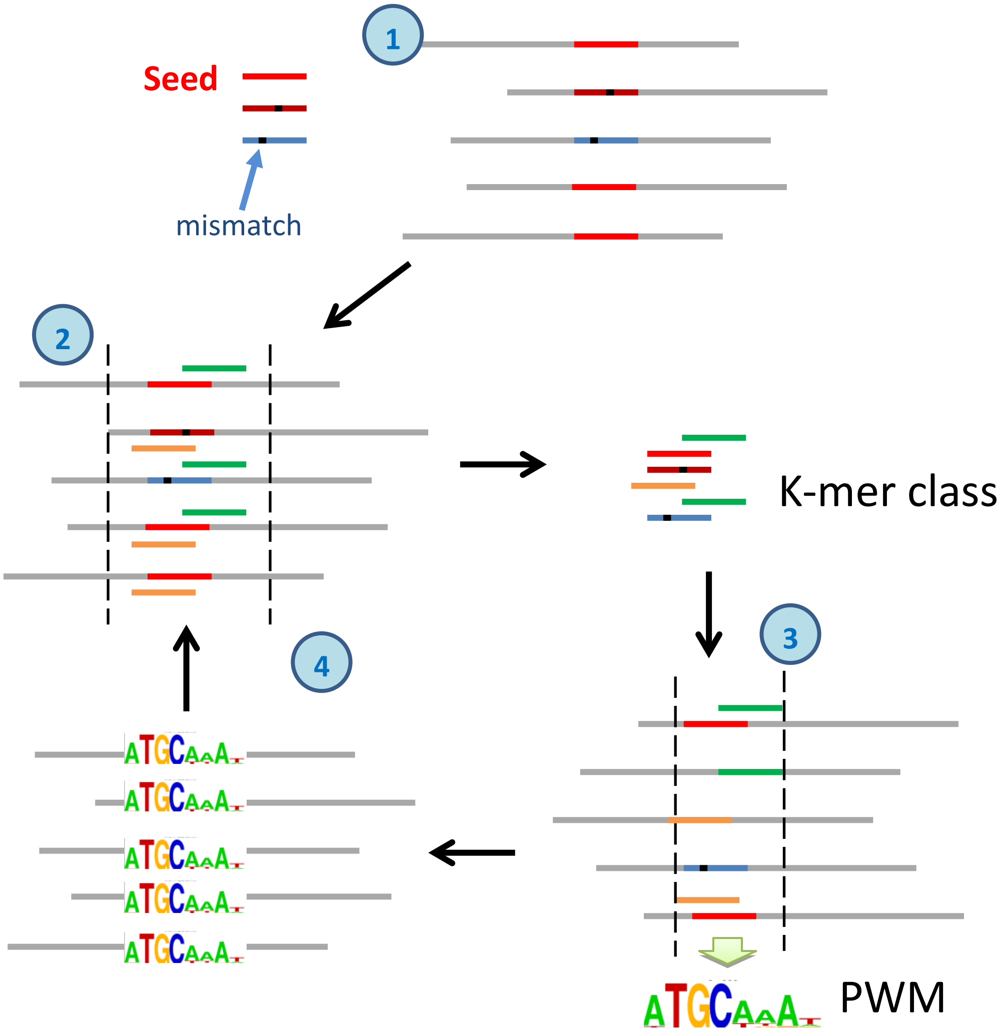

Supplement: Figure S14 — K-mer class motif clustering. (PDF) [file pcbi.1002638.s017.pdf]
