# Supplementary material for: High Resolution Genome Wide Binding Event Finding and Motif Discovery Reveals Transcription Factor Spatial Binding Constraints
Source: PLoS Comput Biol. 2012 Aug 9;8(8):e1002638. doi: 10.1371/journal.pcbi.1002638 (PMC3415389; doi:10.1371/journal.pcbi.1002638)
Supplement: Table S1 — Known motifs recovered by GEM in ENCODE data. (PDF) [file pcbi.1002638.s018.pdf]

Table S1 Known motifs recovered by GEM in ENCODE data
The 184 GEM discovered primary motifs that match the public database PWM. The PFM of these primary and secondary motifs are in Dataset S2.

AP-2alpha_Snyder_HeLa-S3	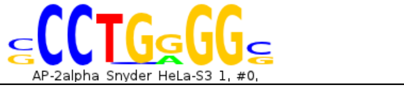	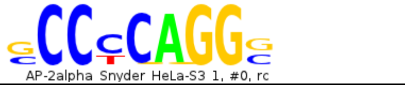
AP-2gamma_Snyder_HeLa-S3	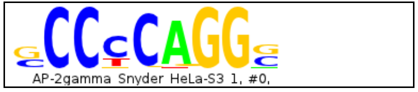	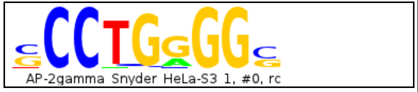
BHLHE40-v041610.1_Myers_HepG2	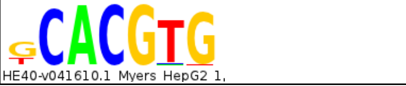	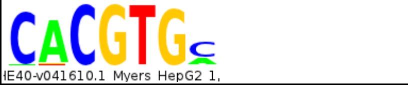
CEBPB_Snyder_HepG2-forskolin	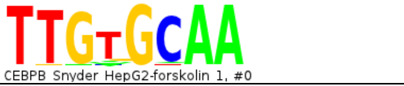	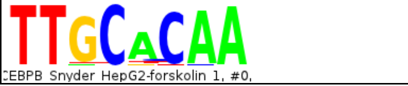
c-Fos_Snyder_HeLa-S3	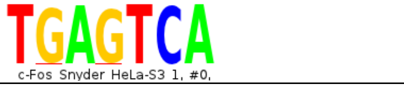	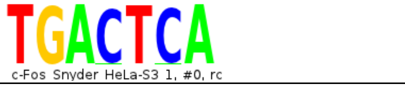
c-Jun_Snyder_GM12878	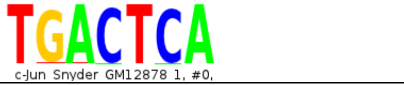	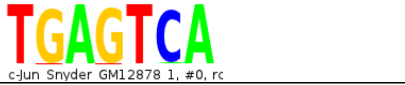
c-Jun_Snyder_HeLa-S3	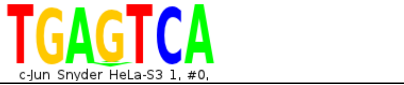	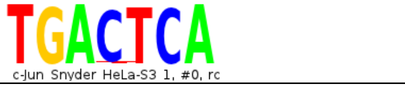
c-Jun_Snyder_HUVEC	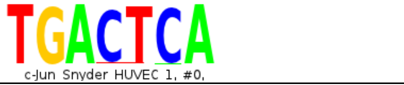	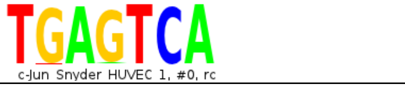
c-Jun_Snyder_K562	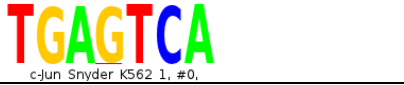	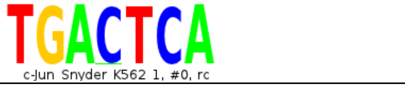
c-Jun_Snyder_K562-IFNa6h	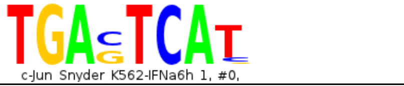	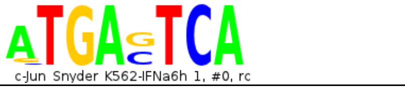
c-Jun_Snyder_K562-IFNg30	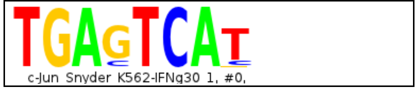	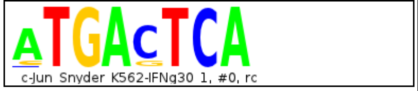
c-Jun_Snyder_K562-IFNg6h	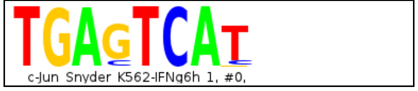	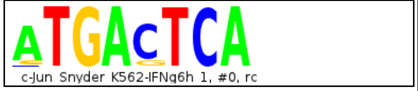
c-Myc_Crawford_GM12878	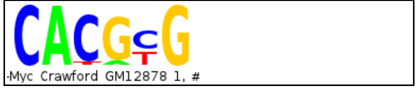	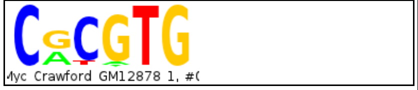
c-Myc_Crawford_HeLa-S3	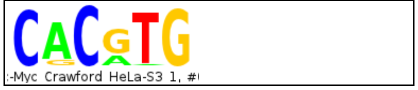	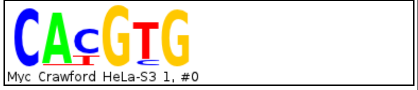
c-Myc_Crawford_HepG2	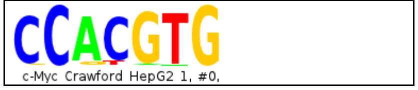	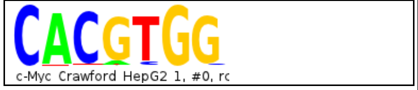
c-Myc_Crawford_HUVEC	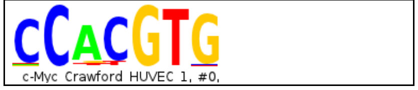	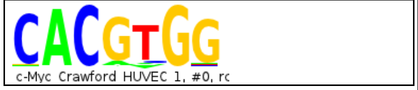
c-Myc_Crawford_K562	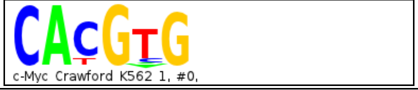	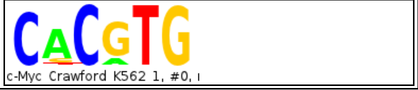

c-Myc_Crawford_MCF-7	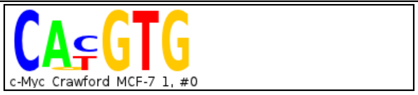	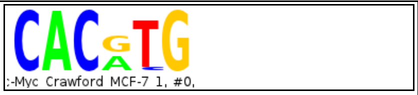
c-Myc_Crawford_MCF-7-estrogen	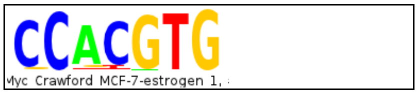	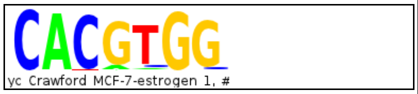
c-Myc_Crawford_MCF-7-vehicle	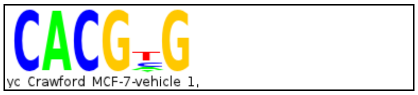	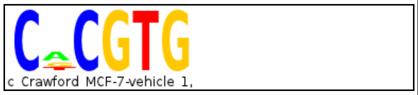
c-Myc_Snyder_GM12878	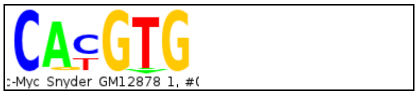	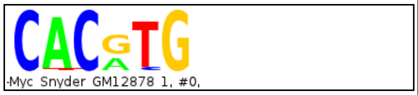
c-Myc_Snyder_HeLa-S3	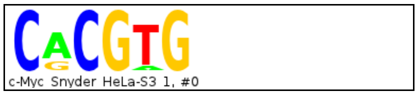	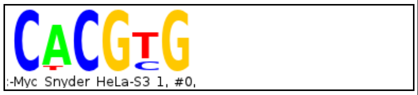
c-Myc_Snyder_K562	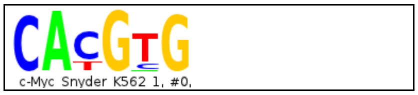	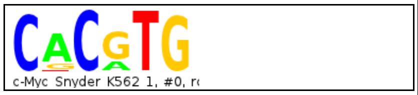
c-Myc_Snyder_K562-IFNa30	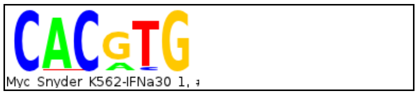	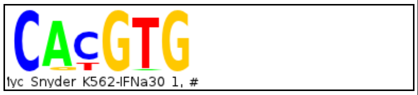
c-Myc_Snyder_K562-IFNa6h	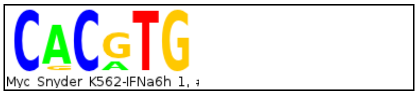	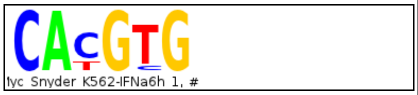
c-Myc_Snyder_K562-IFNg6h	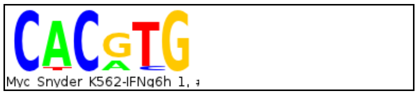	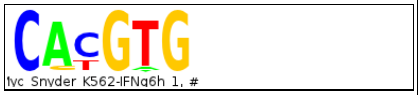
CTCF_Bernstein_GM12878	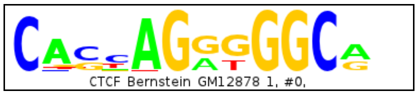	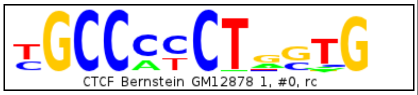
CTCF_Bernstein_H1-hESC	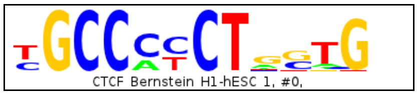	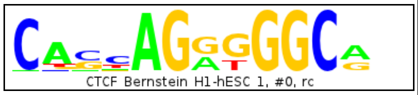
CTCF_Bernstein_HeLa-S3	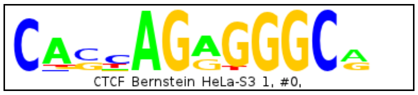	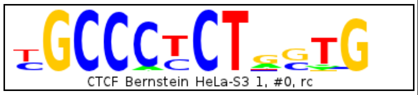
CTCF_Bernstein_HepG2	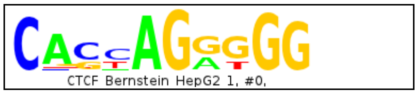	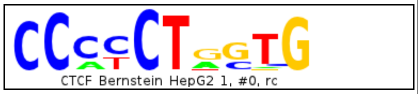
CTCF_Bernstein_HMEC	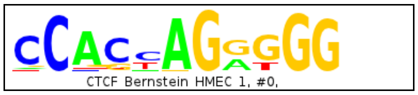	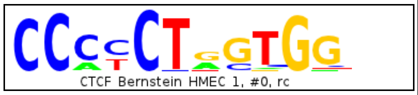
CTCF_Bernstein_HSMM	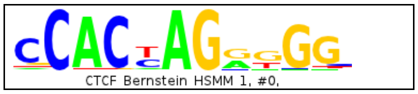	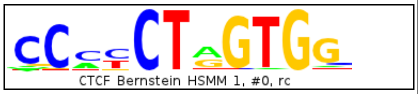
CTCF_Bernstein_HUVEC	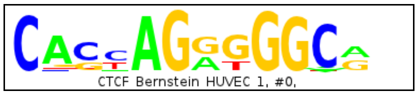	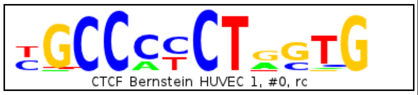
CTCF_Bernstein_K562	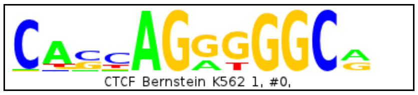	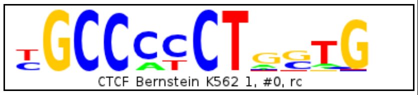
CTCF_Bernstein_NHEK	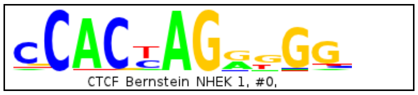	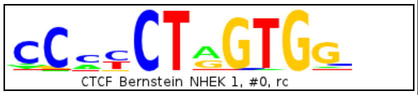
CTCF_Bernstein_NHLF	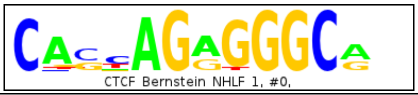	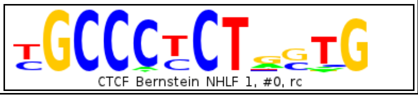

CTCF_Crawford_Gliobla	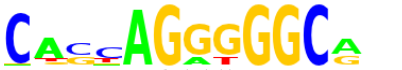 CTCF Crawford Gliobla 1, #0,	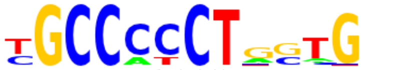 CTCF Crawford Gliobla 1, #0, rc
CTCF_Crawford_GM12878	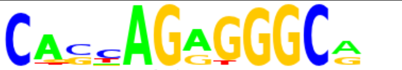 CTCF Crawford GM12878 1, #0,	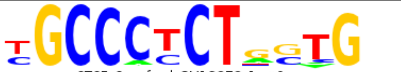 CTCF Crawford GM12878 1, #0, rc
CTCF_Crawford_HeLa-S3	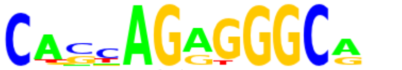 CTCF Crawford HeLa-S3 1, #0,	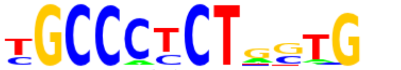 CTCF Crawford HeLa-S3 1, #0, rc
CTCF_Crawford_HepG2	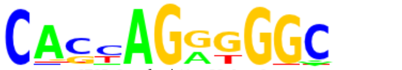 CTCF Crawford HepG2 1, #0,	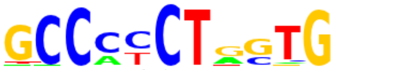 CTCF Crawford HepG2 1, #0, rc
CTCF_Crawford_HUVEC	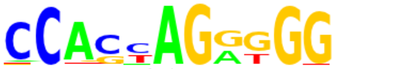 CTCF Crawford HUVEC 1, #0,	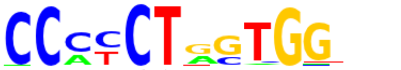 CTCF Crawford HUVEC 1, #0, rc
CTCF_Crawford_K562	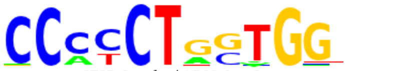 CTCF Crawford K562 1, #0,	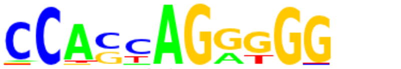 CTCF Crawford K562 1, #0, rc
CTCF_Crawford_MCF-7	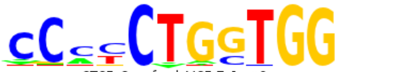 CTCF Crawford MCF-7 1, #0,	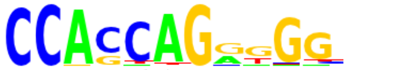 CTCF Crawford MCF-7 1, #0, rc
CTCF_Crawford_MCF-7-estrogen	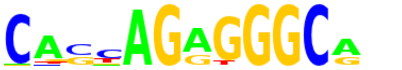 CTCF Crawford MCF-7-estrogen 1, #0,	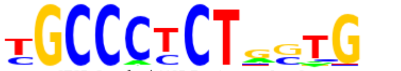 CTCF Crawford MCF-7-estrogen 1, #0, rc
CTCF_Crawford_MCF-7-vehicle	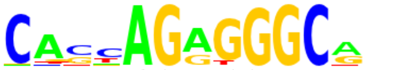 CTCF Crawford MCF-7-vehicle 1, #0,	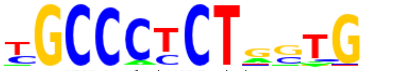 CTCF Crawford MCF-7-vehicle 1, #0, rc
CTCF--SC-5916-PCR1x_Myers_A549-EtOH-0.02pct	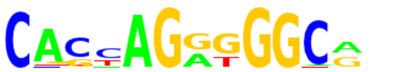 CTCF--SC-5916-PCR1x Myers A549-EtOH-0.02pct 1, #0,	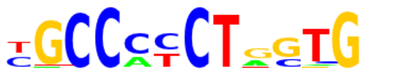 CTCF--SC-5916-PCR1x Myers A549-EtOH-0.02pct 1, #0, r
CTCF--SC-5916-v041610.1_Myers_HepG2	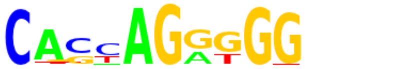 CTCF--SC-5916-v041610.1 Myers HepG2 1, #0,	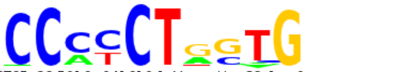 TCF--SC-5916-v041610.1 Myers HepG2 1, #0,
CTCF--SC-5916-v041610.2_Myers_H1-hESC	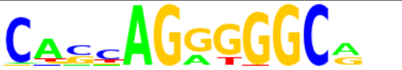 CTCF--SC-5916-v041610.2 Myers H1-hESC 1, #0,	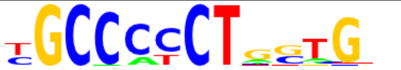 CTCF--SC-5916-v041610.2 Myers H1-hESC 1, #0, rc
CTCF--SC-5916-v041610.2_Myers_T-47D-DMSO-0.02pct	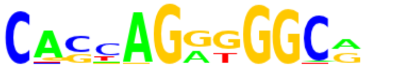 TCF--SC-5916-v041610.2 Myers T-47D-DMSO-0.02pct 1, #	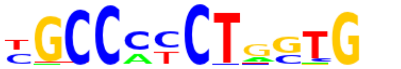 CF--SC-5916-v041610.2 Myers T-47D-DMSO-0.02pct 1, #
CTCF_Stam_AG04449	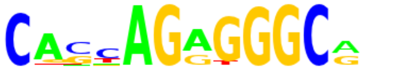 CTCF Stam AG04449 1, #0,	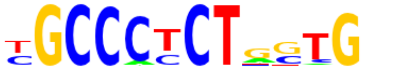 CTCF Stam AG04449 1, #0, rc
CTCF_Stam_AG04450	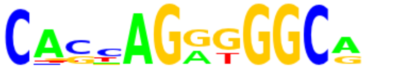 CTCF Stam AG04450 1, #0,	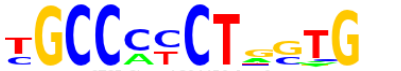 CTCF Stam AG04450 1, #0, rc
CTCF_Stam_AG09309	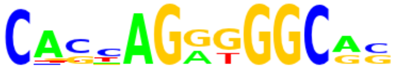 CTCF Stam AG09309 1, #0,	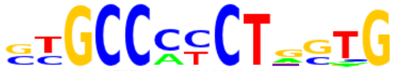 CTCF Stam AG09309 1, #0, rc
CTCF_Stam_AG09319	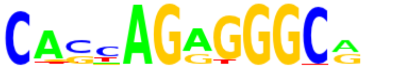 CTCF Stam AG09319 1, #0,	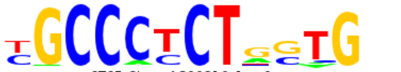 CTCF Stam AG09319 1, #0, rc
CTCF_Stam_AG10803	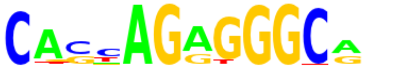 CTCF Stam AG10803 1, #0,	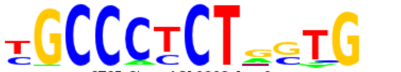 CTCF Stam AG10803 1, #0, rc

CTCF_Stam_AoAF	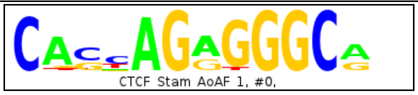	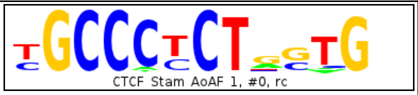
CTCF_Stam_BJ	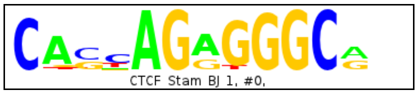	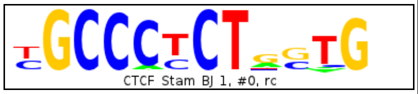
CTCF_Stam_GM12878	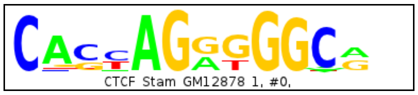	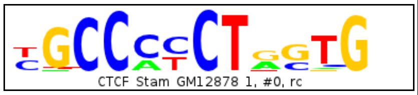
CTCF_Stam_HA-sp	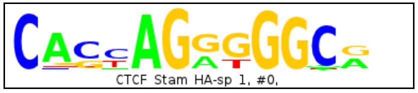	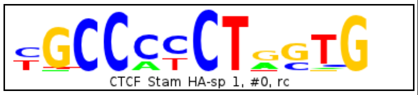
CTCF_Stam_HBMEC	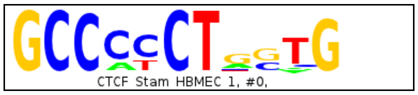	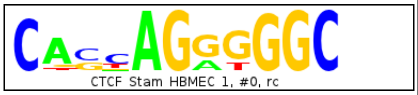
CTCF_Stam_HCFaa	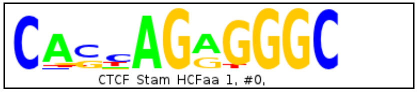	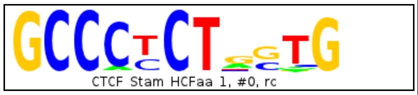
CTCF_Stam_HCPEpiC	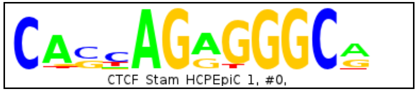	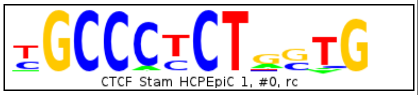
CTCF_Stam_HEEpiC	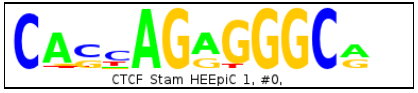	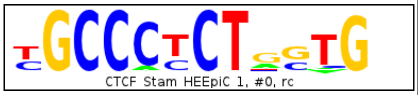
CTCF_Stam_HeLa-S3	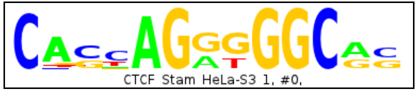	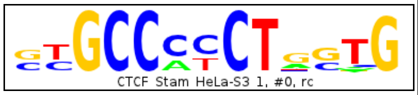
CTCF_Stam_HepG2	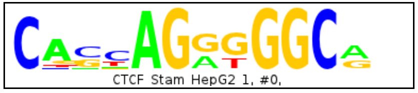	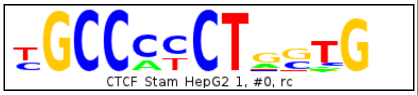
CTCF_Stam_HMEC	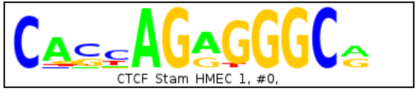	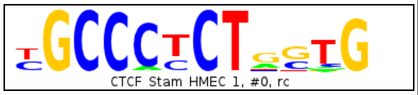
CTCF_Stam_HMF	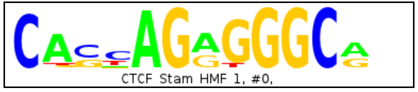	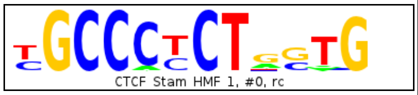
CTCF_Stam_HPAF	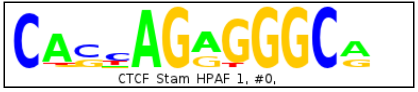	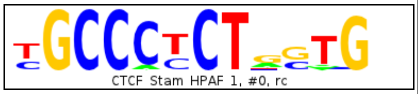
CTCF_Stam_HPF	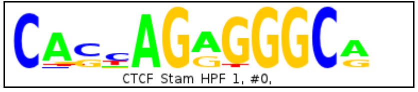	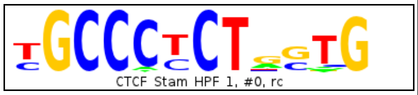
CTCF_Stam_HRPEpiC	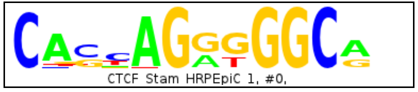	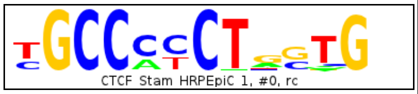
CTCF_Stam_HUVEC	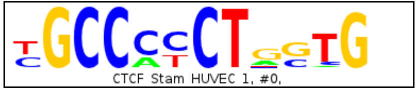	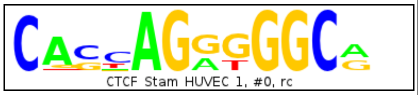
CTCF_Stam_K562	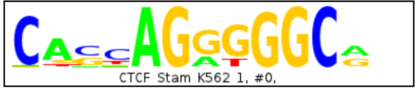	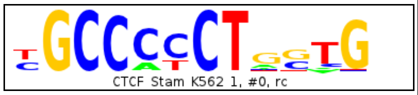
CTCF_Stam_NHEK	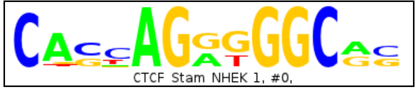	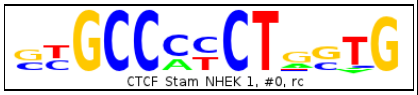
CTCF-v041610.2_Myers_SK-N-SH-	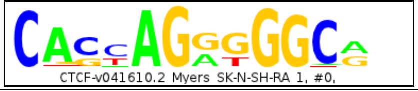	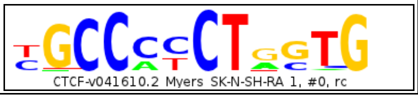

RA		
EBF1--SC-137065-PCR1x_Myers_GM12878	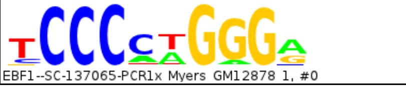	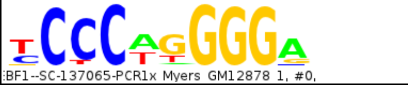
EBF-PCR1x_Myers_GM12878	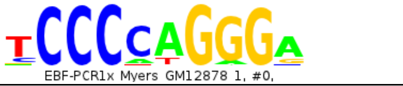	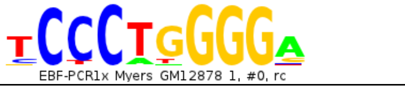
Egr-1-PCR2x_Myers_GM12878	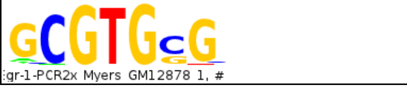	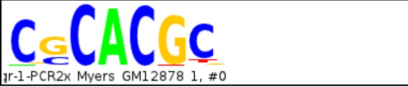
Egr-1-v041610.1_Myers_GM12878	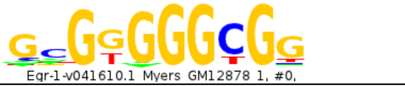	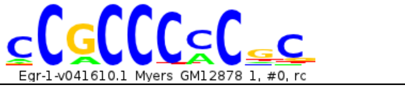
Egr-1-v041610.1_Myers_K562	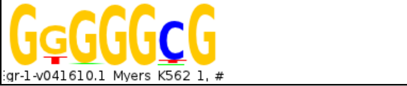	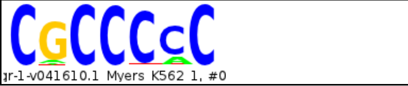
Egr-1-v041610.2_Myers_H1-hESC	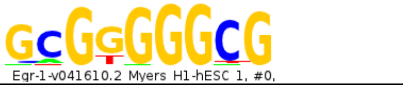	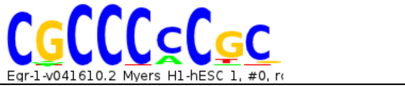
ELF1--SC-631-v041610.1_Myers_GM12878	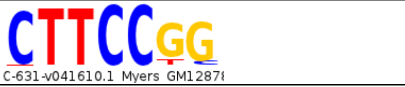	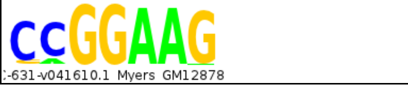
ELF1--SC-631-v041610.1_Myers_HepG2	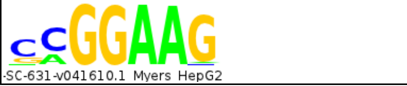	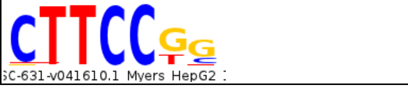
ELF1--SC-631-v041610.2_Myers_K562	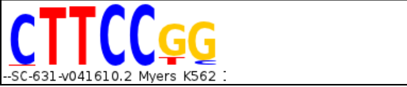	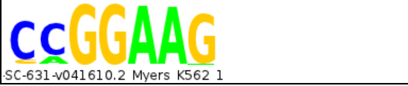
ERalpha-a-v041610.2_Myers_ECC-1-Genistein-100nM	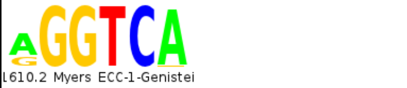	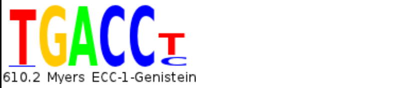
ERalpha-a-v041610.2_Myers_T-47D-Estradiol-10nM	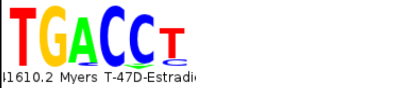	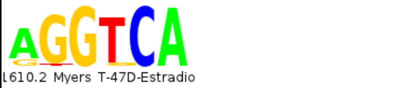
ERalpha-a-v041610.2_Myers_T-47D-Genistein-100nM	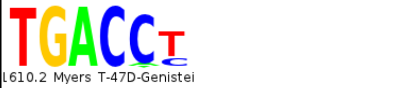	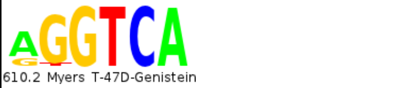
ERRA_Snyder_HepG2-forskolin	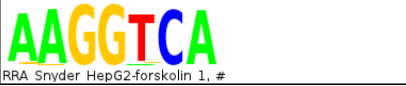	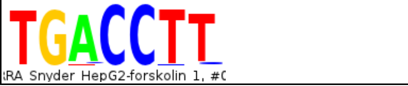
ETS1-PCR1x_Myers_GM12878	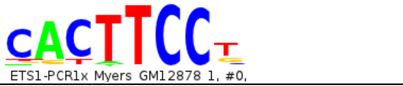	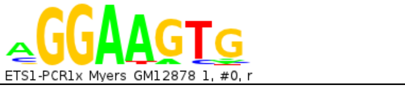
FOS-eGFP_White_K562	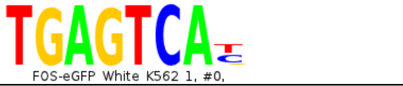	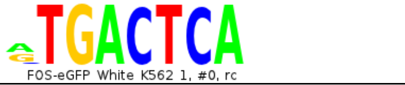
FOSL1--SC-183-v041610.1_Myers_K562	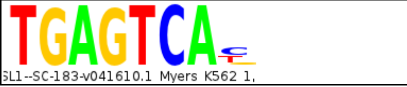	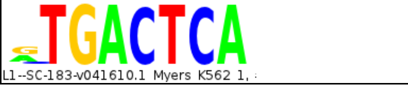
FOSL2-v041610.1_Myers_HepG2	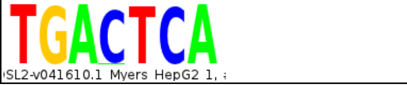	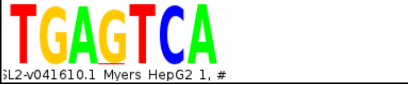

FOXA1--SC-101058-v041610.1_Myers_HepG2	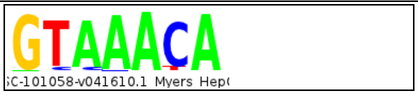	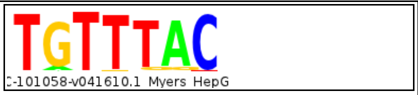
FOXA1--SC-6553-v041610.1_Myers_HepG2	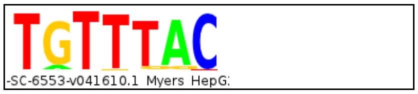	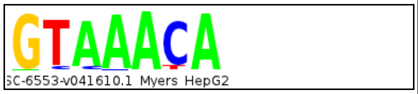
FOXA2--SC-6554-v041610.1_Myers_HepG2	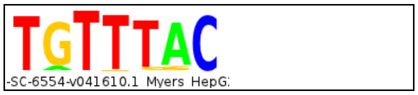	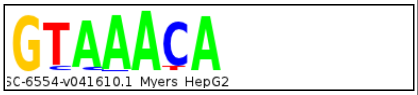
GABP-PCR1x_Myers_HeLa-S3	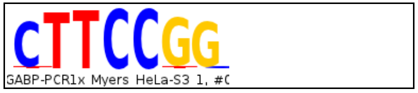	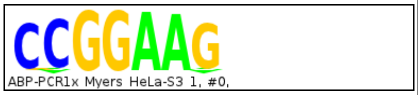
GABP-PCR2x_Myers_GM12878	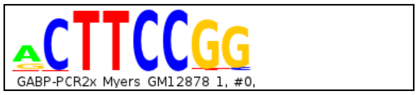	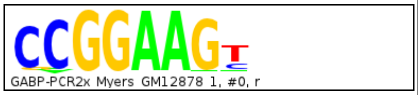
GABP-PCR2x_Myers_HepG2	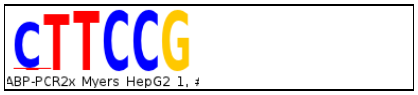	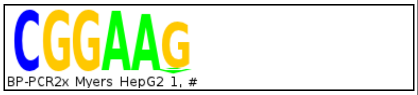
GABP-v041610.1_Myers_K562	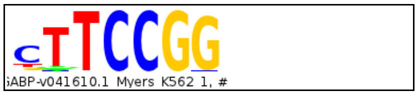	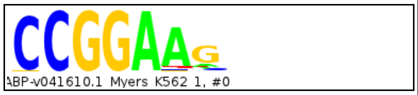
GATA-1_Snyder_K562b	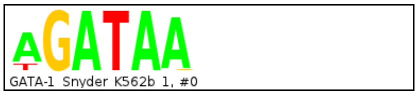	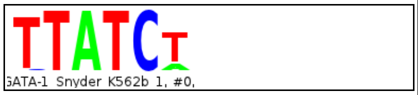
GATA2-eGFP_White_K562	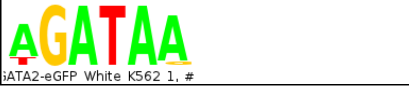	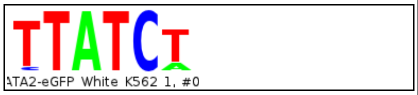
GATA2--SC-267-PCR1x_Myers_K562	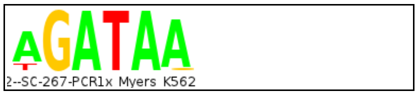	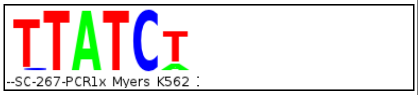
GATA-2_Snyder_K562b	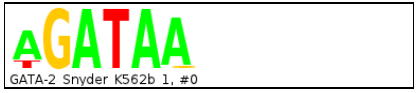	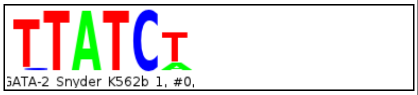
GATA3--SC-268-v041610.2_Myers_T-47D-DMSO-0.02pct	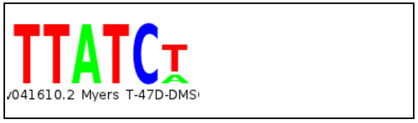	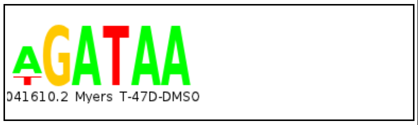
GR-PCR1x_Myers_A549-DEX-50nM	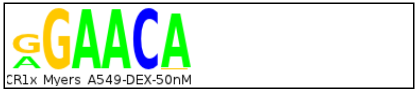	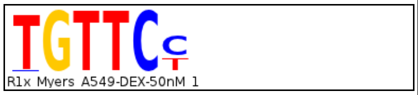
GR-PCR1x_Myers_A549-DEX-5nM	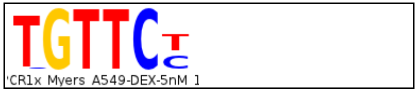	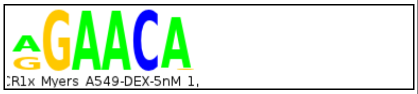
GR-PCR2x_Myers_A549-DEX-100nM	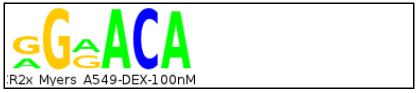	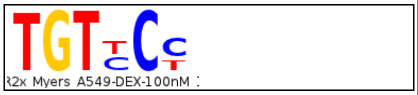
HNF4A--SC-8987-v041610.1_Myers_HepG2	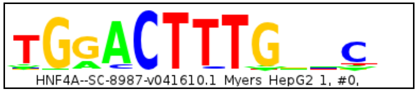	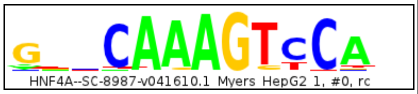
HNF4A_Snyder_HepG2-forskolin	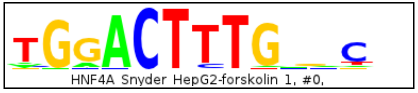	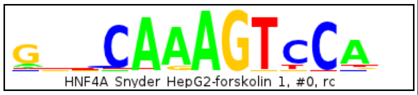
HNF4G--SC-6558-v041610.1_Myers_HepG2	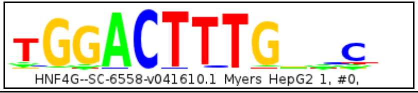	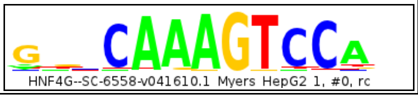

HSF1_Snyder_HepG2-forskolin	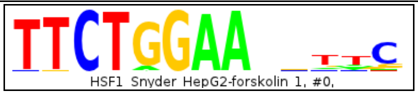	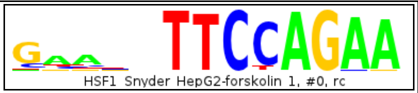
JunB-eGFP_White_K562	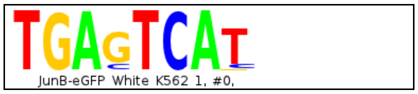	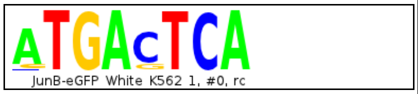
JunD-eGFP_White_K562	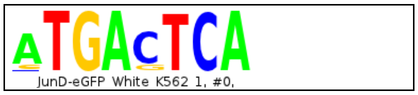	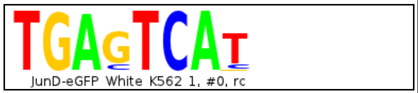
JunD-PCR1x_Myers_HepG2	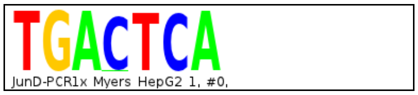	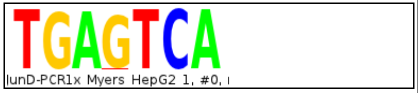
JunD_Snyder_GM12878	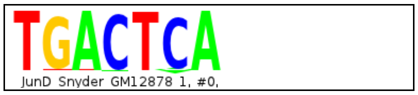	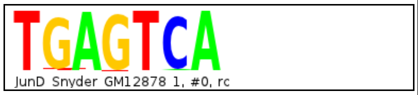
JunD_Snyder_HeLa-S3	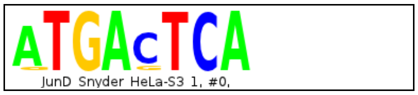	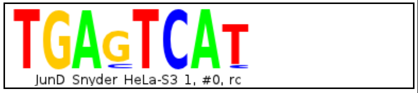
JunD-v041610.2_Myers_H1-hESC	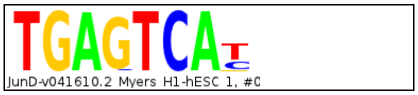	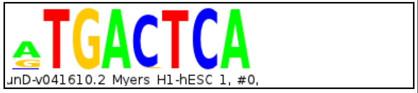
Max_Snyder_GM12878	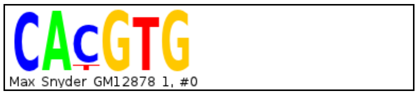	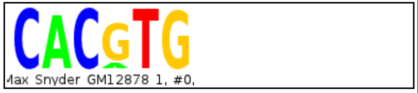
Max_Snyder_HeLa-S3	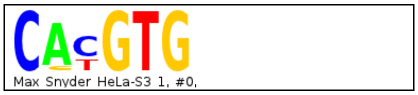	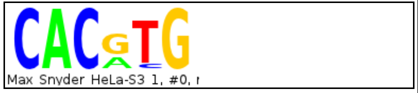
Max_Snyder_HUVEC	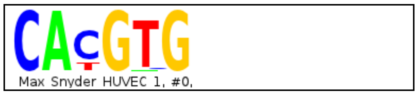	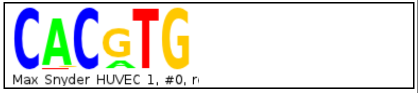
Max_Snyder_K562	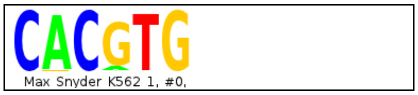	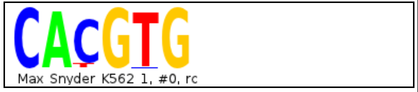
Max-v041610.2_Myers_K562	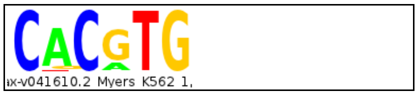	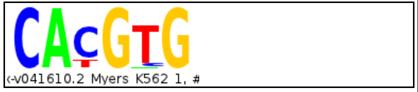
MEF2A-PCR1x_Myers_GM12878	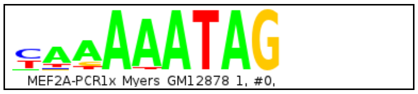	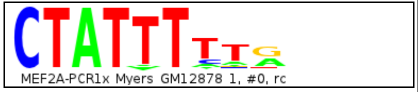
MEF2C--SC-13268-v041610.1_Myers_GM12878	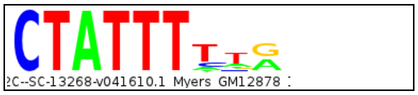	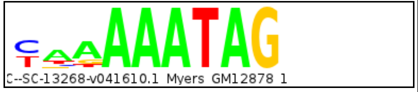
NF-E2_Snyder_K562	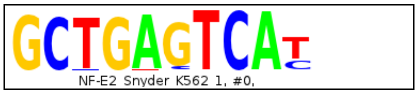	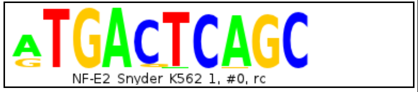
NFKB_Snyder_GM10847	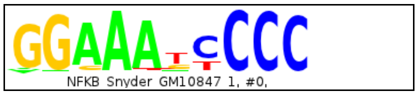	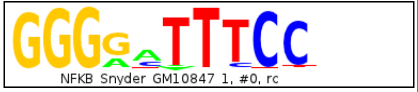
NFKB_Snyder_GM12878	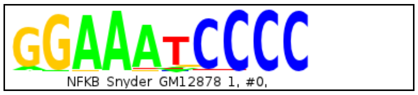	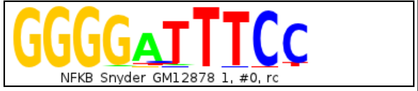
NFKB_Snyder_GM12878-TNFa	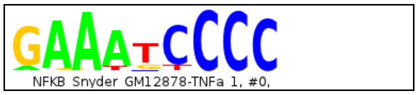	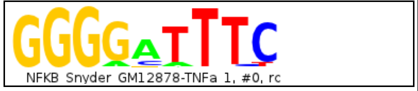
NFKB_Snyder_GM12891	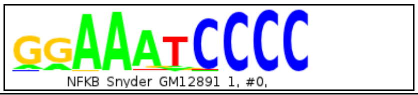	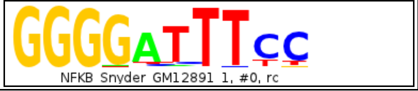

NFKB_Snyder_GM12892	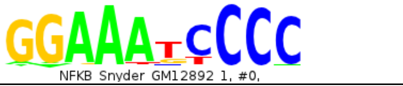	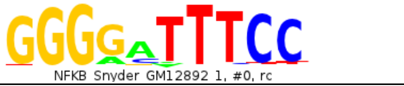
NFKB_Snyder_GM15510	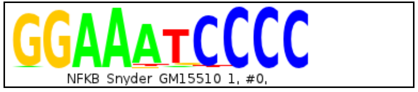	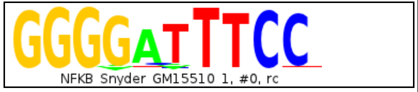
NFKB_Snyder_GM18505	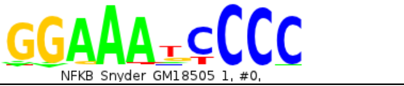	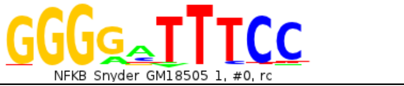
NFKB_Snyder_GM18526	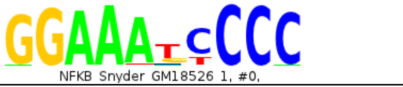	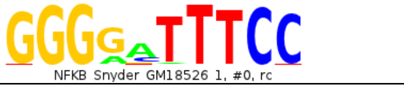
NFKB_Snyder_GM18951	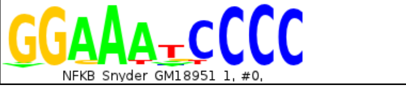	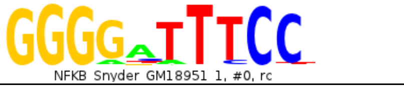
NFKB_Snyder_GM19099	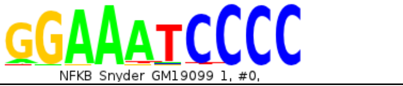	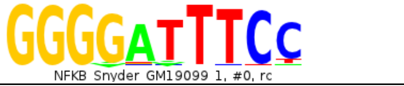
NFKB_Snyder_GM19193	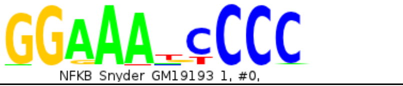	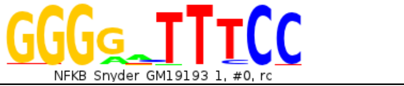
Nrf1_Snyder_HeLa-S3	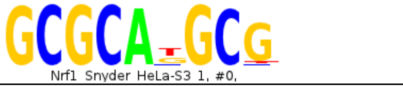	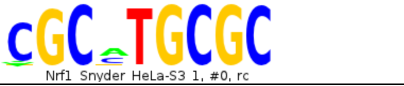
NRSF-PCR1x_Myers_H1-hESC	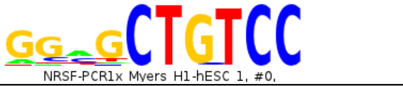	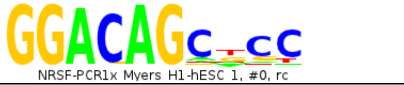
NRSF-PCR1x_Myers_HeLa-S3	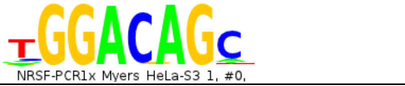	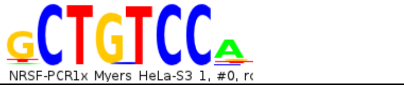
NRSF-PCR2x_Myers_GM12878	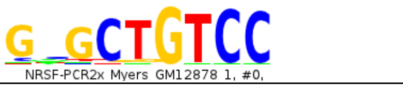	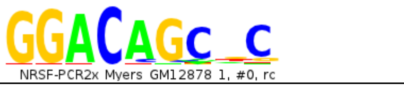
NRSF-PCR2x_Myers_HepG2	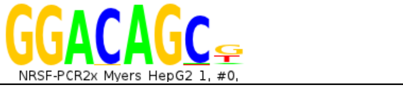	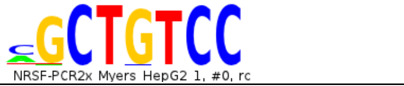
NRSF-PCR2x_Myers_PANC-1	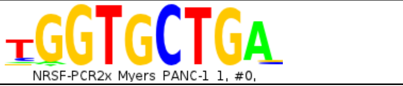	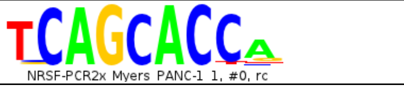
NRSF-PCR2x_Myers_SK-N-SH	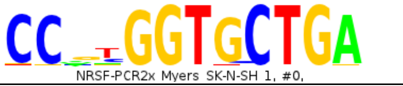	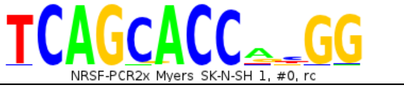
NRSF-PCR2x_Myers_U87	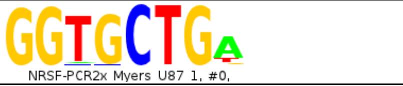	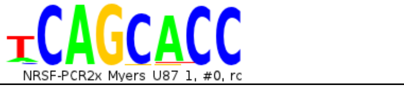
NRSF-v041610.2_Myers_H1-hESC	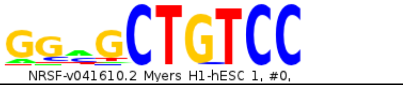	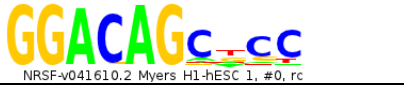
NRSF-v041610.2_Myers_K562	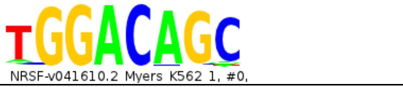	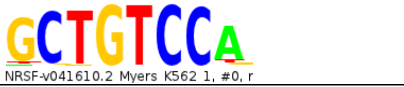
PAX5-C20-PCR1x_Myers_GM12878	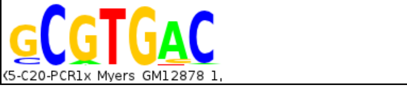	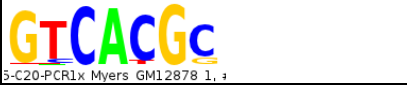
PAX5-C20-v041610.1_Myers_GM12891	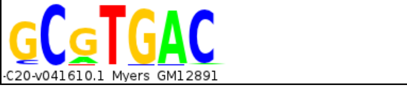	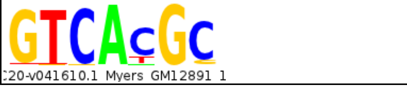

PAX5-C20-v041610.1_Myers_GM12892	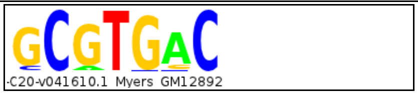	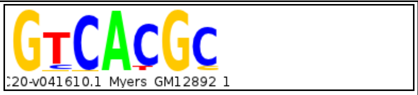
POU2F2-PCR1x_Myers_GM12878	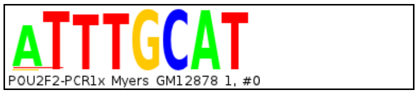	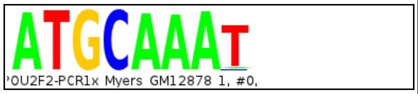
POU2F2-PCR1x_Myers_GM12891	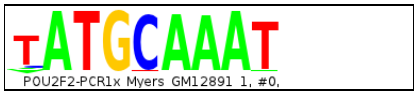	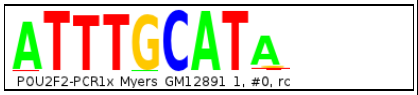
POU5F1--SC-9081-v041610.2_Myers_H1-hESC	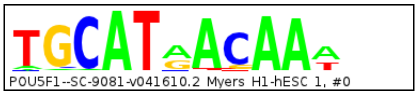	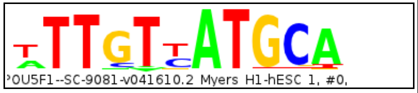
PU.1-PCR1x_Myers_GM12878	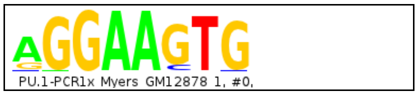	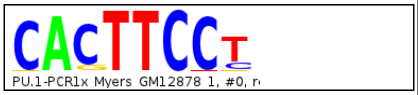
PU.1-PCR1x_Myers_GM12891	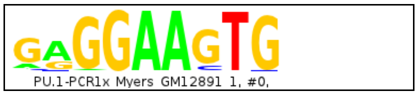	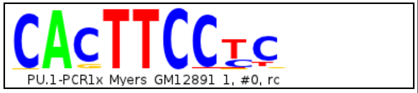
PU.1-PCR1x_Myers_K562	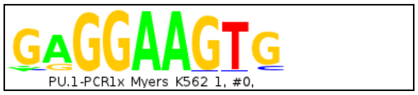	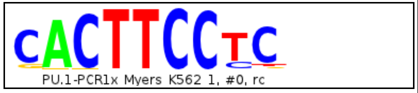
RXRA-PCR1x_Myers_HepG2	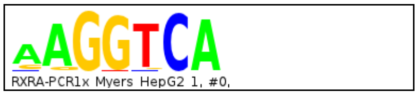	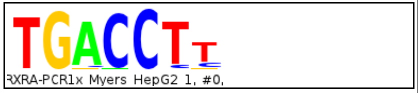
RXRA-v041610.2_Myers_H1-hESC	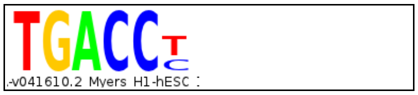	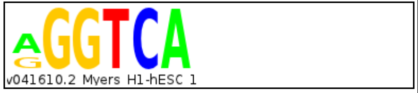
SREBP1_Snyder_HepG2-pravastatin	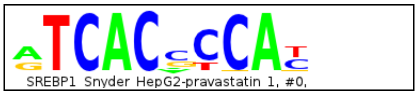	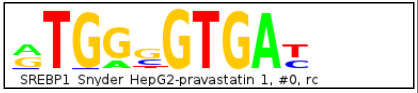
SRF-PCR1x_Myers_H1-hESC	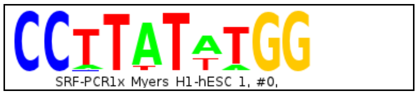	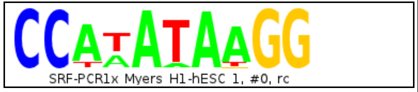
SRF-PCR2x_Myers_GM12878	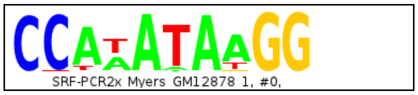	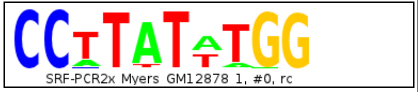
SRF-v041610.1_Myers_GM12878	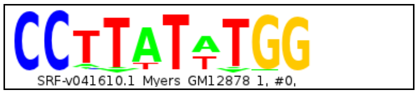	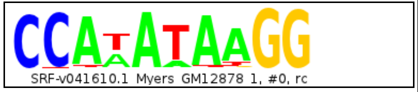
SRF-v041610.1_Myers_HepG2	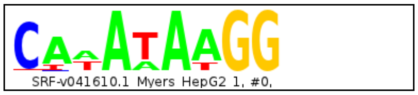	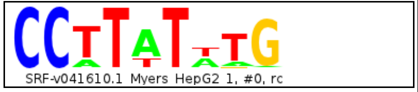
SRF-v041610.1_Myers_K562	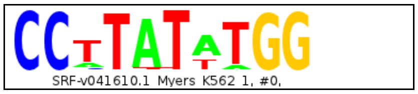	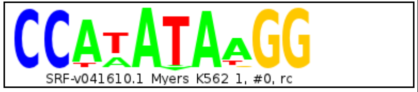
STAT1_Snyder_HeLa-S3-IFNg30	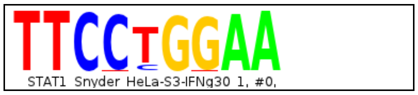	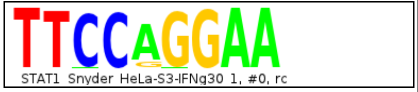
STAT1_Snyder_K562-IFNa30	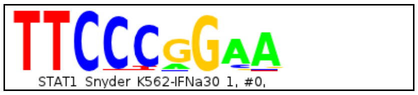	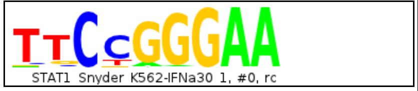
STAT1_Snyder_K562-IFNa6h	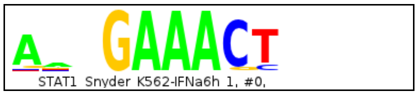	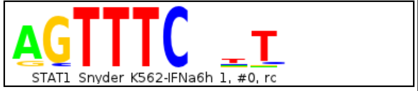
STAT1_Snyder_K562-IFNg30	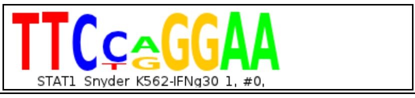	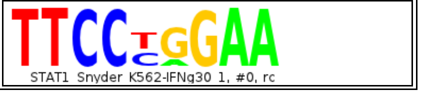

STAT1_Snyder_K562-IFNg6h	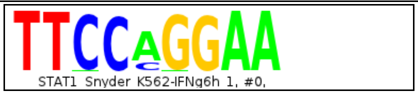	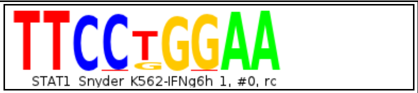
TCF12-PCR1x_Myers_GM12878	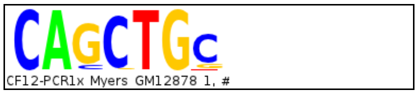	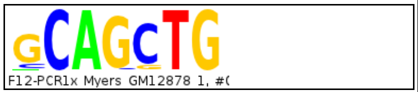
TCF4_Snyder_HCT-116	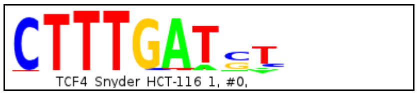	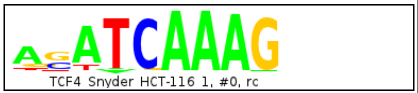
USF-1-PCR1x_Myers_A549-EtOH-0.02pct	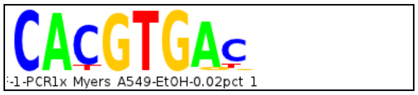	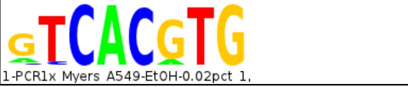
USF-1-PCR1x_Myers_H1-hESC	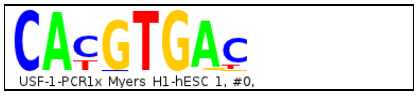	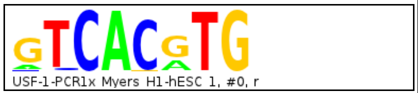
USF-1-PCR1x_Myers_HepG2	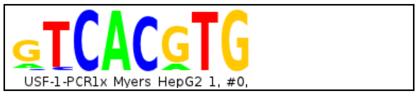	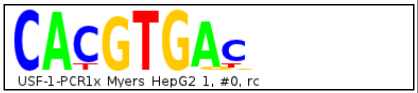
USF-1-PCR2x_Myers_GM12878	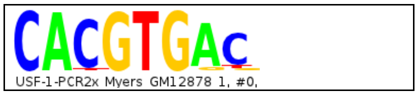	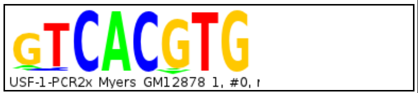
USF1--SC-8983-v041610.2_Myers_SK-N-SH-RA	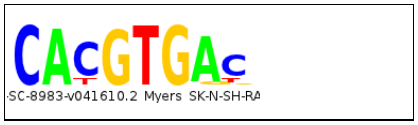	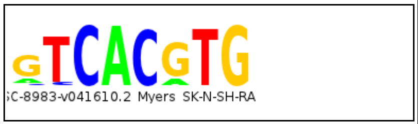
USF-1-v041610.1_Myers_K562	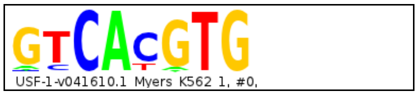	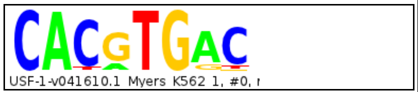
YY1--SC-281-v041610.1_Myers_GM12891	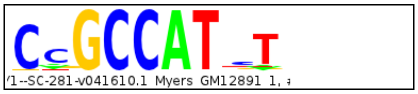	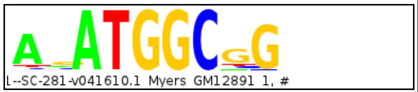
YY1--SC-281-v041610.2_Myers_H1-hESC	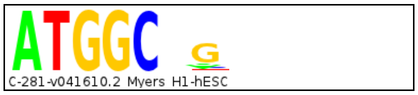	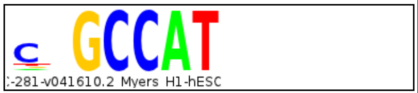
YY1--SC-281-v041610.2_Myers_SK-N-SH-RA	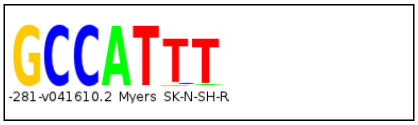	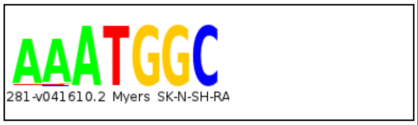
YY1_Snyder_GM12878	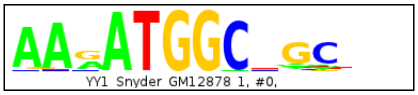	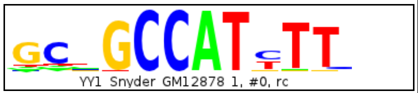
YY1_Snyder_K562b	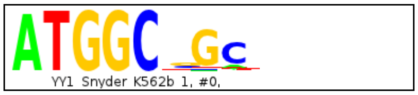	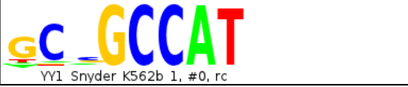
YY1_Snyder_NT2-D1	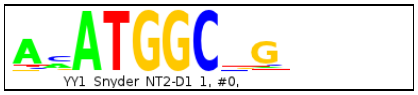	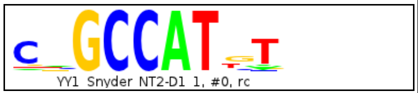
YY1-v041610.1_Myers_GM12892	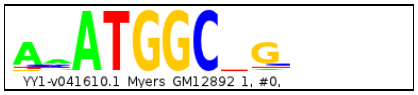	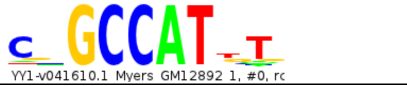
YY1-v041610.1_Myers_K562	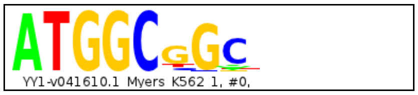	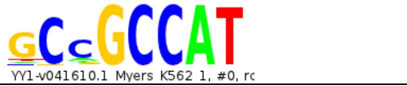
YY1-v041610.2_Myers_K562	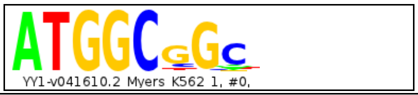	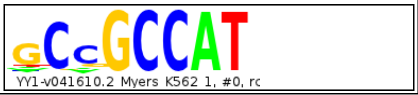

ZEB1--SC-25388-v041610.2_Myers_GM12878	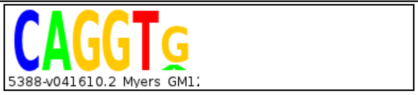	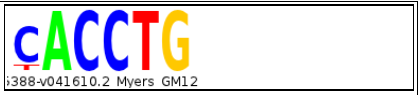
