# Supplementary material for: High Resolution Genome Wide Binding Event Finding and Motif Discovery Reveals Transcription Factor Spatial Binding Constraints
Source: PLoS Comput Biol. 2012 Aug 9;8(8):e1002638. doi: 10.1371/journal.pcbi.1002638 (PMC3415389; doi:10.1371/journal.pcbi.1002638)
Supplement: Table S2 — Performance of motif discovery methods by individual ENCODE ChIP-Seq experiments. (PDF) [file pcbi.1002638.s019.pdf]

Table S1 Performance of motif discovery methods by individual ENCODE ChIP-Seq experiments

The numbers represent the rank of PWM that matches the expected database PWM.

ChIP-Seq Expt	TF	gem	meme	weeder	mdscan	alignace	Posmo	Posmo_5k	ChIP-Munk	ChIP-Munk_5k	hms	hms_5k
AP-2alpha_Snyder_HeLa-S3	AP-2	0	0	0	0	0	0	0	0	0	0	111
AP-2gamma_Snyder_HeLa-S3	AP-2	0	0	0	0	0	0	0	0	0	0	0
ATF3-PCR1x_Myers_GM12878	ATF3	111	3	16	111	25	111	111	111	111	111	111
ATF3_Snyder_K562	ATF3	2	0	12	111	15	111	111	111	111	0	0
ATF3-v041610.1_Myers_HepG2	ATF3	4	2	16	6	18	111	111	111	111	111	111
BHLHE40-v041610.1_Myers_HepG2	BHLHE40	0	0	0	0	0	0	111	0	0	0	0
CEBPB_Snyder_HepG2-forskolin	CEBPB	0	0	5	0	0	0	0	0	0	111	0
c-Fos_Snyder_GM12878	c-Fos	111	111	111	8	111	111	111	111	111	111	111
c-Fos_Snyder_HeLa-S3	c-Fos	0	0	0	111	0	0	0	0	0	0	0
c-Fos_Snyder_K562	c-Fos	1	111	111	7	111	111	111	111	111	111	0
c-Jun_Snyder_GM12878	c-Jun	0	0	1	0	0	0	111	0	0	0	0
c-Jun_Snyder_HeLa-S3	c-Jun	0	0	0	0	0	0	0	0	0	0	0
c-Jun_Snyder_HUVEC	c-Jun	0	0	0	0	0	0	0	0	0	0	0
c-Jun_Snyder_K562	c-Jun	0	0	0	0	0	0	0	0	0	0	0
c-Jun_Snyder_K562-IFNa6h	c-Jun	0	0	0	0	0	0	111	0	0	0	0
c-Jun_Snyder_K562-IFNg30	c-Jun	0	0	0	111	0	0	0	0	0	0	0
c-Jun_Snyder_K562-IFNg6h	c-Jun	0	0	0	0	0	0	0	0	0	0	0
c-Myc_Crawford_GM12878	c-Myc	0	0	0	0	111	0	0	0	111	0	111
c-Myc_Crawford_HeLa-S3	c-Myc	0	0	0	0	5	0	0	0	111	0	0
c-Myc_Crawford_HepG2	c-Myc	0	0	0	4	2	0	0	0	111	0	111
c-Myc_Crawford_HUVEC	c-Myc	0	0	0	0	1	0	111	0	111	0	111
c-Myc_Crawford_K562	c-Myc	0	0	0	1	111	0	111	0	111	0	111
c-Myc_Crawford_MCF-7	c-Myc	0	0	0	0	7	0	0	111	111	0	0
c-Myc_Crawford_MCF-7-estrogen	c-Myc	0	0	0	0	2	0	0	0	111	0	111
c-Myc_Crawford_MCF-7-vehicle	c-Myc	0	0	0	0	4	0	111	0	111	0	111
c-Myc_Snyder_GM12878	c-Myc	0	0	0	111	2	0	0	0	0	0	0
c-Myc_Snyder_HeLa-S3	c-Myc	0	0	0	0	0	0	0	0	111	0	0
c-Myc_Snyder_K562	c-Myc	0	0	0	0	2	0	0	0	111	0	111
c-Myc_Snyder_K562-IFNa30	c-Myc	0	0	0	0	0	0	0	0	0	0	0
c-Myc_Snyder_K562-IFNa6h	c-Myc	0	0	0	0	0	0	0	0	0	0	0

c-Myc_Snyder_ K562-IFNg6h	c-Myc	0	0	111	111	0	0	0	0	0	0	0
CTCF_Bernstein_ GM12878	CTCF	0	0	0	111	0	0	0	0	0	0	0
CTCF_Bernstein_ H1-hESC	CTCF	0	0	0	0	0	0	0	0	0	0	0
CTCF_Bernstein_ HeLa-S3	CTCF	0	0	0	0	0	0	1	0	0	0	0
CTCF_Bernstein_ HepG2	CTCF	0	0	0	0	0	0	0	0	0	0	0
CTCF_Bernstein_ HMEC	CTCF	0	0	1	0	0	0	1	0	0	0	0
CTCF_Bernstein_ HSMM	CTCF	0	0	1	0	0	0	0	0	0	0	0
CTCF_Bernstein_ HUVEC	CTCF	0	0	0	0	0	0	0	0	0	0	0
CTCF_Bernstein_K562	CTCF	0	0	0	0	0	0	0	0	0	0	0
CTCF_Bernstein_NHEK	CTCF	0	0	1	0	0	0	0	0	0	0	0
CTCF_Bernstein_NHLF	CTCF	0	0	0	0	0	0	0	0	0	0	0
CTCF_Crawford_ Gliobla	CTCF	0	0	0	0	0	0	0	0	0	0	0
CTCF_Crawford_ GM12878	CTCF	0	0	0	0	0	0	0	0	0	0	0
CTCF_Crawford_ HeLa-S3	CTCF	0	0	0	0	0	0	0	0	0	0	0
CTCF_Crawford_ HepG2	CTCF	0	0	1	0	0	0	0	0	0	0	0
CTCF_Crawford_ HUVEC	CTCF	0	0	1	0	0	0	0	0	0	0	0
CTCF_Crawford_K562	CTCF	0	0	0	1	0	0	0	0	0	0	0
CTCF_Crawford_ MCF-7	CTCF	0	0	0	0	0	0	1	0	0	0	0
CTCF_Crawford_ MCF-7-estrogen	CTCF	0	0	0	111	0	0	0	0	0	0	0
CTCF_Crawford_ MCF-7-vehicle	CTCF	0	0	0	0	0	0	0	0	0	0	0
CTCF--SC-5916-PCR1x_ Myers_A549-EtOH-0.02pct	CTCF	0	0	0	0	0	111	0	0	0	0	0
CTCF--SC-5916-v041610.1_Myers_ HepG2	CTCF	0	0	0	0	0	111	0	0	0	0	0
CTCF--SC-5916-v041610.2_Myers_ H1-hESC	CTCF	0	0	0	0	0	111	1	0	0	0	0
CTCF--SC-5916-v041610.2_Myers_ T-47D-DMSO-0.02pct	CTCF	0	0	0	0	0	111	0	0	0	0	0
CTCF_Stam_AG04449	CTCF	0	0	0	0	0	0	0	0	0	0	0
CTCF_Stam_AG04450	CTCF	0	0	0	0	0	0	0	0	0	0	0
CTCF_Stam_AG09309	CTCF	0	0	1	0	0	0	0	0	0	0	0
CTCF_Stam_AG09319	CTCF	0	0	0	0	0	0	0	0	0	0	0
CTCF_Stam_AG10803	CTCF	0	0	1	0	0	0	0	0	0	0	0
CTCF_Stam_AoAF	CTCF	0	0	0	0	0	0	0	0	0	0	0
CTCF_Stam_BJ	CTCF	0	0	1	0	0	0	0	0	0	0	0
CTCF_Stam_GM12878	CTCF	0	0	0	111	0	0	0	0	0	0	0
CTCF_Stam_HA-sp	CTCF	0	0	1	0	0	0	0	0	0	0	0
CTCF_Stam_HBMEC	CTCF	0	0	1	0	0	0	0	0	0	0	0
CTCF_Stam_HCFaa	CTCF	0	0	1	0	0	0	0	0	0	0	0

CTCF_Stam_HCPEpiC	CTCF	0	0	0	0	0	0	0	0	0	0	0
CTCF_Stam_HEEpiC	CTCF	0	0	0	0	0	0	0	0	0	0	0
CTCF_Stam_HeLa-S3	CTCF	0	0	0	0	0	0	0	0	0	0	0
CTCF_Stam_HepG2	CTCF	0	0	0	0	0	0	0	0	0	0	0
CTCF_Stam_HMEC	CTCF	0	0	0	0	0	0	1	0	0	0	0
CTCF_Stam_HMF	CTCF	0	0	111	0	0	0	0	0	0	0	0
CTCF_Stam_HPAF	CTCF	0	0	0	0	0	0	0	0	0	0	0
CTCF_Stam_HPF	CTCF	0	0	0	0	0	0	0	0	0	0	0
CTCF_Stam_HRPEpiC	CTCF	0	0	1	0	0	0	0	0	0	0	0
CTCF_Stam_HUVEC	CTCF	0	0	0	0	0	0	0	0	0	0	0
CTCF_Stam_K562	CTCF	0	0	1	0	0	0	0	0	0	0	0
CTCF_Stam_NHEK	CTCF	0	0	1	0	0	0	0	0	0	0	0
CTCF-v041610.2_ Myers _SK-N-SH-RA	CTCF	0	0	0	0	0	0	1	0	0	0	0
EBF1--SC-137065-PCR1x_Myers_GM12878	EBF1	0	0	0	0	0	0	0	0	0	0	0
EBF-PCR1x_Myers_ GM12878	EBF	0	0	1	0	0	0	0	0	0	0	0
Egr-1-PCR2x_Myers_ GM12878	Egr-1	0	0	1	4	0	0	111	0	0	111	0
Egr-1-v041610.1_ Myers_GM12878	Egr-1	0	0	0	0	0	0	0	0	0	0	0
Egr-1-v041610.1_ Myers_K562	Egr-1	0	0	0	0	0	0	0	0	0	0	0
Egr-1-v041610.2_ Myers_H1-hESC	Egr-1	0	0	0	0	0	0	0	0	0	0	0
ELF1--SC-631-v041610.1_ Myers_GM12878	ELF1	0	0	0	0	111	0	111	0	0	0	0
ELF1--SC-631-v041610.1_ Myers_HepG2	ELF1	0	0	0	111	0	0	111	0	0	0	0
ELF1--SC-631-v041610.2_ Myers_K562	ELF1	0	0	0	0	0	0	111	0	0	0	0
ERalpha-a-v041610.2_ Myers_ECC-1-Genistein-100nM	ERalpha-a	0	0	0	111	0	0	0	0	0	0	0
ERalpha-a-v041610.2_ Myers_T-47D-Estradiol-10nM	ERalpha-a	0	0	0	1	0	0	0	0	0	0	0
ERalpha-a-v041610.2_ Myers_T-47D-Genistein-100nM	ERalpha-a	0	0	0	2	0	0	0	0	0	0	0
ERRA_Snyder_ HepG2-forskolin	ERRA	0	0	0	0	0	111	0	0	0	0	0
ETS1-PCR1x_ Myers_GM12878	ETS1	0	2	111	111	16	111	0	111	111	111	0
ETS1-v041610.1_ Myers_K562	ETS1	2	4	111	111	111	111	111	111	111	111	111
FOS-eGFP_ White_K562	FOS	0	0	111	0	0	0	0	0	0	0	0
FOSL1--SC-183-v041610.1_ Myers_K562	FOSL1	0	0	0	111	0	0	0	0	0	0	0
FOSL2-v041610.1_ Myers_HepG2	FOSL2	0	0	0	0	0	0	0	0	0	0	0
FOXA1--SC-101058-	FOXA1	0	0	0	0	0	0	0	0	0	0	0

v041610.1_ Myers_HepG2												
FOXA1--SC-6553-v041610.1_ Myers_HepG2	FOXA1	0	0	0	0	0	0	0	0	0	0	0
FOXA2--SC-6554-v041610.1_ Myers_HepG2	FOXA2	0	0	0	0	0	0	0	0	0	0	0
GABP-PCR1x_ Myers_HeLa-S3	GABP	0	0	0	0	0	0	0	0	0	0	0
GABP-PCR2x_ Myers_GM12878	GABP	0	0	0	0	0	0	0	0	0	0	0
GABP-PCR2x_ Myers_HepG2	GABP	0	0	0	0	0	0	0	0	0	0	0
GABP-v041610.1_ Myers_K562	GABP	0	0	0	0	0	0	0	0	0	0	0
GATA-1_Snyder_K562b	GATA1	0	0	0	111	0	0	0	0	111	0	0
GATA2-eGFP_White_K562	GATA2	0	0	0	0	0	111	0	0	0	0	0
GATA2--SC-267-PCR1x_Myers_K562	GATA2	0	1	0	0	0	111	111	0	0	111	111
GATA-2_Snyder_K562b	GATA2	0	0	0	111	0	111	0	0	0	0	0
GATA3--SC-268-v041610.2_Myers_T-47D-DMSO-0.02pct	GATA3	0	0	0	111	17	111	111	111	111	111	111
GR-PCR1x_Myers_ A549-DEX-500pM	GR	2	0	2	4	2	111	111	0	111	0	0
GR-PCR1x_Myers_ A549-DEX-50nM	GR	0	0	1	0	0	0	0	0	0	0	111
GR-PCR1x_Myers_ A549-DEX-5nM	GR	0	0	0	0	0	111	111	0	0	0	0
GR-PCR2x_Myers_ A549-DEX-100nM	GR	0	0	0	111	0	111	0	0	0	0	0
GR-v041610.2_Myers_ ECC-1-DEX-100nM	GR	1	0	0	0	0	0	111	0	0	0	0
HEY1-PCR1x_ Myers_K562	HEY1	111	111	111	111	111	111	111	111	111	111	111
HEY1-v041610.1_ Myers_HepG2	HEY1	111	111	111	111	111	111	111	111	111	111	111
HNF4A--SC-8987-v041610.1_Myers_ HepG2	HNF4A	0	0	1	0	0	0	0	0	0	0	0
HNF4A_Snyder_ HepG2-forskolin	HNF4A	0	0	1	0	0	0	0	0	0	0	0
HNF4G--SC-6558-v041610.1_ Myers_HepG2	HNF4G	0	0	1	0	0	0	0	0	0	0	0
HSF1_Snyder_ HepG2-forskolin	HSF1	0	0	0	0	5	0	0	0	111	111	111
IRF4-PCR1x_ Myers_GM12878	IRF4	1	0	111	0	0	111	111	0	0	111	111
IRF4--SC-6059-PCR1x _Myers_GM12878	IRF4	1	0	111	6	0	111	111	0	0	111	111
JunB-eGFP_ White_K562	JunB	0	0	0	0	0	0	0	0	0	0	0
JunD-eGFP_ White_K562	JunD	0	0	0	0	0	0	0	0	0	0	0
JunD-PCR1x_ Myers_HepG2	JunD	0	0	0	0	0	0	0	0	0	0	0
JunD_Snyder_	JunD	0	0	1	0	0	0	111	0	0	0	0

GM12878												
JunD_Snyder_HeLa-S3	JunD	0	0	0	0	0	0	0	0	0	0	0
JunD_Snyder_K562	JunD	1	3	111	111	11	111	111	111	111	111	111
JunD-v041610.2_Myers_H1-hESC	JunD	0	1	8	111	12	0	1	111	111	0	111
Max_Snyder_GM12878	Max	0	0	0	0	0	0	0	0	0	0	0
Max_Snyder_HeLa-S3	Max	0	0	0	0	0	0	111	0	0	0	0
Max_Snyder_HUVEC	Max	0	0	0	0	0	0	0	0	0	0	0
Max_Snyder_K562	Max	0	0	0	0	0	0	0	0	0	0	0
Max-v041610.2_Myers_K562	Max	0	0	0	0	0	0	0	0	0	0	0
MEF2A-PCR1x_Myers_GM12878	MEF2A	0	0	0	0	0	0	1	0	0	0	111
MEF2C--SC-13268-v041610.1_Myers_GM12878	MEF2C	0	0	0	0	0	0	1	0	0	0	0
NANOG--SC-33759-v041610.2_Myers_H1-hESC	NANOG	111	111	1	111	111	111	111	111	111	111	111
NF-E2_Snyder_K562	NF-E2	0	0	0	111	0	0	0	0	0	0	0
NFKB_Snyder_GM10847	NFKB	0	0	0	0	0	0	0	0	0	0	0
NFKB_Snyder_GM12878	NFKB	0	0	0	0	0	0	0	0	0	0	111
NFKB_Snyder_GM12878-TNFa	NFKB	0	0	0	111	0	0	0	0	0	0	111
NFKB_Snyder_GM12891	NFKB	0	0	0	0	0	0	0	0	0	0	0
NFKB_Snyder_GM12892	NFKB	0	0	0	0	0	0	0	0	0	0	0
NFKB_Snyder_GM15510	NFKB	0	0	0	0	0	0	111	0	111	0	0
NFKB_Snyder_GM18505	NFKB	0	0	0	0	0	0	0	0	0	0	0
NFKB_Snyder_GM18526	NFKB	0	0	0	0	0	0	0	0	0	0	0
NFKB_Snyder_GM18951	NFKB	0	0	0	0	0	0	0	0	0	0	0
NFKB_Snyder_GM190111	NFKB	0	0	0	0	0	0	0	0	0	0	0
NFKB_Snyder_GM19193	NFKB	0	0	0	0	0	0	0	0	0	0	0
NR4A1-eGFP_White_K562	NR4A1	111	0	1	1	111	111	0	111	111	111	0
Nrf1_Snyder_HeLa-S3	Nrf1	0	0	0	0	0	0	0	0	0	0	0
NRSF-PCR1x_Myers_H1-hESC	NRSF	0	0	0	0	0	0	0	0	0	0	0
NRSF-PCR1x_Myers_HeLa-S3	NRSF	0	0	0	0	0	0	0	0	0	0	0
NRSF-PCR2x_Myers_GM12878	NRSF	0	0	0	0	0	0	0	0	0	0	0
NRSF-PCR2x_Myers_HepG2	NRSF	0	0	0	0	0	0	0	0	0	0	0
NRSF-PCR2x_Myers_PANC-1	NRSF	0	0	0	0	0	0	0	0	0	0	0
NRSF-PCR2x_Myers_SK-N-SH	NRSF	0	0	0	0	0	0	111	0	0	0	0
NRSF-PCR2x_Myers_U87	NRSF	0	0	0	0	0	0	0	0	0	0	0

NRSF-v041610.2_ Myers_H1-hESC	NRSF	0	0	0	0	0	0	0	0	0	0	0
NRSF-v041610.2_ Myers_K562	NRSF	0	0	0	0	0	0	0	0	0	0	0
PAX5-C20-PCR1x_ Myers_GM12878	PAX5-C20	0	0	1	111	16	0	111	0	0	0	111
PAX5-C20-v041610.1_ Myers_GM12891	PAX5-C20	0	0	1	0	3	0	111	0	111	0	111
PAX5-C20-v041610.1_ Myers_GM12892	PAX5-C20	0	0	1	111	111	0	111	0	111	0	111
PAX5-N19-PCR1x_ Myers_GM12878	PAX5-N19	111	0	111	2	24	0	111	0	111	0	111
Pbx3-PCR1x_ Myers_GM12878	Pbx3	111	111	10	111	111	111	111	111	111	111	111
POU2F2-PCR1x_ Myers_GM12878	POU2F2	0	0	0	0	0	0	1	111	111	0	0
POU2F2-PCR1x_ Myers_GM12891	POU2F2	0	0	0	0	0	0	0	0	111	0	0
POU5F1--SC-9081-v041610.2_ Myers_H1-hESC	POU5F1	0	0	0	0	0	0	0	0	0	0	0
PU.1-PCR1x_ Myers_GM12878	PU.1	0	0	0	0	0	0	0	0	0	0	0
PU.1-PCR1x_ Myers_GM12891	PU.1	0	0	111	0	0	0	0	0	0	0	0
PU.1-PCR1x_ Myers_K562	PU.1	0	0	0	0	0	0	0	0	0	0	0
RXRA-PCR1x_ Myers_HepG2	RXRA	0	0	0	0	0	111	0	0	0	0	0
RXRA-v041610.2_ Myers_H1-hESC	RXRA	0	0	0	0	15	111	111	0	111	0	111
SIX5-PCR1x_ Myers_K562	SIX5	111	111	111	111	111	111	111	111	111	111	111
SP1-PCR1x_ Myers_GM12878	SP1	111	3	111	111	12	111	111	111	111	111	111
SP1-PCR1x_ Myers_HepG2	SP1	111	3	111	111	0	111	111	111	0	111	111
SP1-PCR1x_ Myers_K562	SP1	1	3	111	111	3	111	111	0	0	111	111
SREBP1_Snyder_ HepG2-insulin	SREBP1	111	2	111	3	111	111	111	111	111	111	111
SREBP1_Snyder_ HepG2-pravastatin	SREBP1	0	1	3	0	18	111	1	111	111	111	111
SREBP2_Snyder_ HepG2-pravastatin	SREBP2	1	1	111	7	11	111	1	111	111	111	111
SRF-PCR1x_Myers_ H1-hESC	SRF	0	0	0	0	0	0	1	0	111	0	0
SRF-PCR2x_ Myers_GM12878	SRF	0	0	2	0	15	0	1	0	111	0	0
SRF-v041610.1_ Myers_GM12878	SRF	0	0	1	0	14	0	2	0	111	0	0
SRF-v041610.1_ Myers_HepG2	SRF	0	0	0	0	0	0	2	0	0	0	0
SRF-v041610.1_ Myers_K562	SRF	0	0	2	0	11	0	1	0	111	0	0
STAT1_Snyder_HeLa-S3-IFNg30	STAT1	0	0	0	0	0	0	0	0	0	0	0
STAT1_Snyder_K562-IFNa30	STAT1	0	0	0	0	0	0	9	0	0	0	0
STAT1_Snyder_K562-IFNa6h	STAT1	0	0	1	0	0	0	0	0	0	0	0

STAT1_Snyder_K562-IFNg30	STAT1	0	0	0	0	0	0	0	0	0	0	0
STAT1_Snyder_K562-IFNg6h	STAT1	0	0	0	0	0	0	0	0	0	0	111
STAT2_Snyder_K562-IFNa30	STAT2	1	1	2	111	4	0	5	111	111	111	111
STAT2_Snyder_K562-IFNa6h	STAT2	111	5	111	111	19	111	111	111	111	111	111
TCF12-PCR1x_ Myers_GM12878	TCF12	0	0	0	0	0	0	0	0	0	0	0
TCF12-PCR1x_ Myers_HepG2	TCF12	111	8	111	111	111	111	111	111	111	111	111
TCF4_Snyder_ HCT-116	TCF4	0	0	0	111	1	0	0	0	111	0	111
USF-1-PCR1x_Myers_ A549-EtOH-0.02pct	USF-1	0	0	0	0	0	0	0	0	0	0	0
USF-1-PCR1x_Myers_ H1-hESC	USF-1	0	0	0	0	0	0	0	0	0	0	0
USF-1-PCR1x_Myers_HepG2	USF-1	0	0	0	0	0	0	0	0	0	0	0
USF-1-PCR2x_Myers_ GM12878	USF-1	0	0	0	0	0	0	0	0	0	0	0
USF1--SC-8983-v041610.2_ Myers_ SK-N-SH-RA	USF-1	0	0	0	0	0	0	0	0	0	0	0
USF-1-v041610.1_ Myers_K562	USF-1	0	0	0	0	0	0	0	0	0	0	0
YY1--SC-281-v041610.1_ Myers_GM12891	YY1	0	111	0	0	111	111	111	111	111	111	111
YY1--SC-281-v041610.2_ Myers_ H1-hESC	YY1	0	111	0	0	111	111	0	111	111	111	111
YY1--SC-281-v041610.2_ Myers_ SK-N-SH-RA	YY1	0	4	0	0	0	111	111	111	111	111	111
YY1_Snyder_GM12878	YY1	0	111	0	0	111	111	0	111	111	111	111
YY1_Snyder_K562b	YY1	0	111	0	0	111	111	0	111	111	111	111
YY1_Snyder_NT2-D1	YY1	0	111	0	0	111	111	0	111	111	111	111
YY1-v041610.1_ Myers_GM12892	YY1	0	111	111	0	111	111	0	111	111	111	111
YY1-v041610.1_ Myers_K562	YY1	0	111	0	0	111	111	0	111	111	111	111
YY1-v041610.2_ Myers_K562	YY1	0	111	0	0	111	111	0	111	111	111	111
ZBTB33-PCR1x_ Myers_GM12878	ZBTB33	111	111	111	111	111	111	111	111	111	111	111
ZBTB33-PCR1x_Myers_HepG2	ZBTB33	111	111	111	111	111	111	111	111	111	111	111
ZBTB33-v041610.1_ Myers_HepG2	ZBTB33	111	111	111	111	111	111	111	111	111	111	111
ZBTB7A--SC-34508-v041610.1_ Myers_K562	ZBTB7A	111	0	0	111	111	111	111	111	111	111	111
ZEB1--SC-25388-v041610.2_ Myers_GM12878	ZEB1	0	0	0	0	0	0	0	0	111	0	111

Note: 111 indicates none of the motif discovered by that method matches expected PWM.
