# Supplementary material for: High Resolution Genome Wide Binding Event Finding and Motif Discovery Reveals Transcription Factor Spatial Binding Constraints
Source: PLoS Comput Biol. 2012 Aug 9;8(8):e1002638. doi: 10.1371/journal.pcbi.1002638 (PMC3415389; doi:10.1371/journal.pcbi.1002638)
Supplement: Table S3 — Overall performance of motif discovery methods for ENCODE data. (PDF) [file pcbi.1002638.s020.pdf]

Table S1 Overall performance of motif discovery methods for ENCODE data
The overall performance of each method is computed by counting the number of success experiments that are equal to or higher than the rank. These are the same data as in Figure 2.

Rank	gem	meme	weeder	mdscan	alignace	posmo	Posmo _5k	ChIP-Munk	ChIP-Munk_5k	hms	hms_5k
**Top1**	184	179	148	159	156	163	145	175	152	172	156
**Top2**	192	184	177	163	158	163	160				
**Top3**	195	187	181	165	163	163	162				
**Top4**	195	192	182	166	165	163	162				
**Top5**	196	194	182	169	167	163	162				
**Top6**	196	195	183	169	169	163	163				
