# Supplementary material for: High Resolution Genome Wide Binding Event Finding and Motif Discovery Reveals Transcription Factor Spatial Binding Constraints
Source: PLoS Comput Biol. 2012 Aug 9;8(8):e1002638. doi: 10.1371/journal.pcbi.1002638 (PMC3415389; doi:10.1371/journal.pcbi.1002638)
Supplement: Table S4 — Novel motifs discovered by GEM in ENCODE data. (PDF) [file pcbi.1002638.s021.pdf]

Table S4 Novel motifs discovered by GEM in ENCODE data
GEM was applied on 9 ENCODE ChIP-Seq data with no publically described binding motifs. Six out of 9 GEM discovered primary motifs are consistent with expected binding sequences reported in literature.

**Name**	**GEM Motif**	**Rank of GEM Motif**	**Expected Binding Pattern**	**Why expected?**	**Citations**
BATF	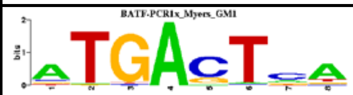	0	AP-1 /TRE element<br><br>TGACTCA	BATF is a member of the AP-1 family of transcription factors. These factors are dimers of proteins from many families including Jun, Fos, Maf, and ATF and bind to the AP-1/TRE element.	T. Hai and T. Curran. Cross-family dimerization of transcription factors Fos/Jun and ATF/CREB alters DNA binding specificity. *Proc. Natl. Acad. Sci. USA*, 88:3720-3724, 11111.<br><br>W. Ise, et al. The transcription factor BATF controls the global regulators of class-switch recombination in both B cells and T cells. *Nature*. 12:536-542, 2011.
CTCFL	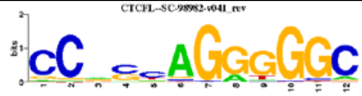	0	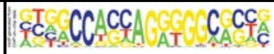	CTCFL/BORIS arose from a duplication of CTCF during vertebrate evolution and the protein structure remains very similar to CTCF and binds similar DNA sequences.	T. Hore, J. Deakin, and J. Graves. The evolution of Epigenetic Regulators CTCF and BORIS/CTCFL in Amniotes. *PLOS Genetics*, 4(8): e1000169, 2008. doi: 10.1371/journal.pgen.1000169<br><br>T. Kim, et al. Analysis of the Vertebrate Insulator Protein CTCF-Binding Sites in the Human Genome. *Cell*, 128(6): 1231-1245, 2007.
Sp2	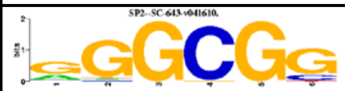	2	(5′-GGGCGGGAC-3′)	This consensus was determined using SELEX for the Sp2 protein (not a related protein).	K. Moorefield, S. Fry, and J. Horowitz. Sp2 DNA Binding Activity and trans-Activation Are Negatively Regulated in Mammalian Cells. *J. Bioc. Chem.,* 279:13911-13924, 2004.
TR4	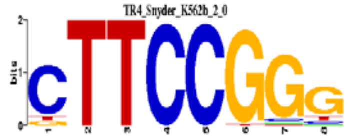	0, 1	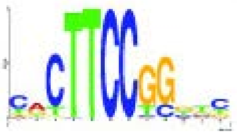<br>ETS motif	ETS and DR1 motifs have been shown to be enriched at TR4 binding sites. GEM recovers both of these motifs that resulted from previous CHIP-seq motif analysis.	H. O'Geen, et al. Genome-wide binding of the orphan nuclear receptor TR4 suggests its general role in fundamental biological processes. *BMC Genomics,* 11:689, 2010. doi:10.1186/1471-2164-

	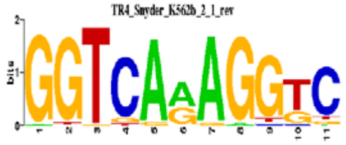		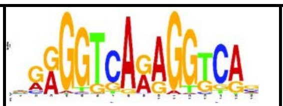 DR1 motif		11-689.<br><br>Loss of TR4 Orphan Nuclear Receptor Reduces Phosphoenolpyruvate Carboxykinase-Mediated Gluconeogenesis
FoxP2	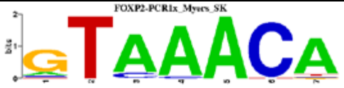	0	FOXP2 binding site CAAATT<br><br>(AAAT core)	FOXP2 has been shown to bind to a AAAT core.	E. Spiteri et al. Identification of the Transcriptional Targets of FOXP2, a Gene Linked to Speech and Language, in Developing Human Brain. *Am. Jour. Hum. Genetics,* 81:1144-1157, 2007.
THAP1	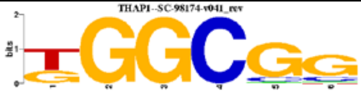 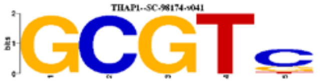	1	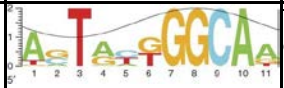	The expected consensus was found by sequence analysis of several binding sites determined by SELEX for several THAPs including THAP1. GEM identifies the major groove portion. Some of the other logos may be the minor groove.	A. Sabogal et al. THAP proteins target specific DNA sites through bipartite recognition of adjacent major and minor grooves. *Nat. Struct. Mol. Biol.*, 17:117-123, 2010. doi: 10.1038/nsmb. 1742.
ZNF263	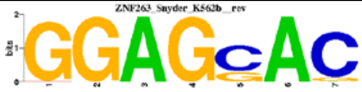	0	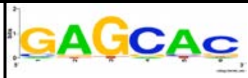 (claimed secondary motif at ZNF263 sites)	The paper proposed a 24-nt motif from CHIP-seq data as the primary site, but also found this secondary motif in a significant portion of the top binding sites.	S. Frietze, et al. Genomic Targets of the KRAB and SCAN Domain-containing Zinc Finger Protein 263. *Am. Soc. Bioc. Mol. Bio.*, 285(2):1393-1403, 2010.
ZNF274	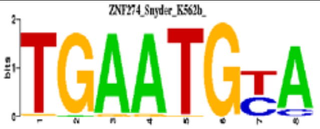	3	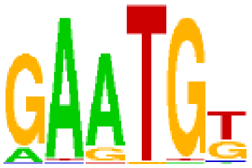	ZNF274 CHIP-seq motif analysis proposed 28 significant motifs at ZNF274 binding sites, most of which have an ATG core. The logo here is for the top motif.	S. Frietze, et al. ZNF274 Recruits the Histone Methyltransferase ZETDB1 to the 3' Ends of ZNF Genes. *PLOS One,* 5(12):e15082, 2010. doi: 10.1371/journal.pone.0015082.
ZZZ3	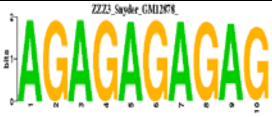	0	GA-repeat	ZZZ3 shares considerable identity with members of the ATAC family of proteins, which are similar to GA-repeat binding proteins.	T. Suganuma, et al. ATAC is a double histone acetyltransferase complex that stimulates nucleosome sliding. *Nat. Struct. Mol. Biol.*, 15:364-372, 2008.
