# Supplementary material for: High Resolution Genome Wide Binding Event Finding and Motif Discovery Reveals Transcription Factor Spatial Binding Constraints
Source: PLoS Comput Biol. 2012 Aug 9;8(8):e1002638. doi: 10.1371/journal.pcbi.1002638 (PMC3415389; doi:10.1371/journal.pcbi.1002638)
Supplement: Table S5 — Motifs of mouse ES cell factors discovered by GEM. (PDF) [file pcbi.1002638.s022.pdf]

Table S5 Motifs of mouse ES cell factors discovered by GEM
The PFM of these primary motifs and secondary motifs are in Dataset S3.

TF	GEM motif PWM	GEM motif reverse compliment
ES_c-Myc	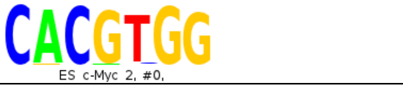	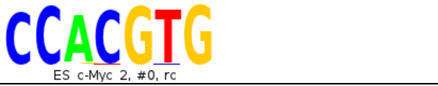
ES_Ctcf	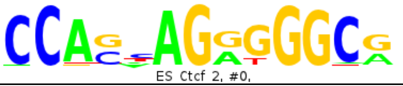	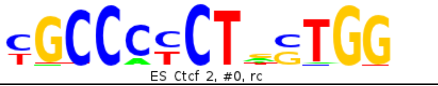
ES_E2f1	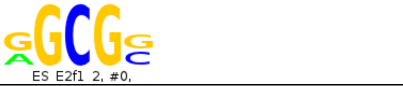	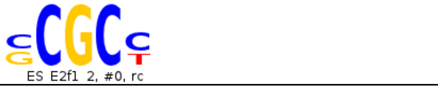
ES_Esrrb	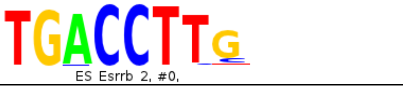	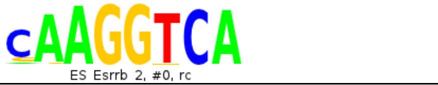
ES_Klf4	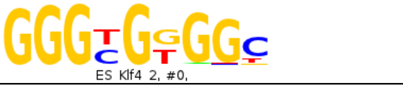	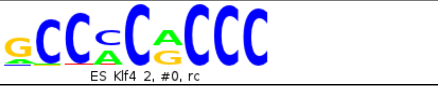
ES_Nanog	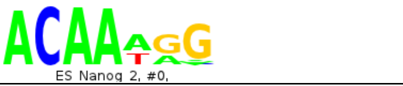	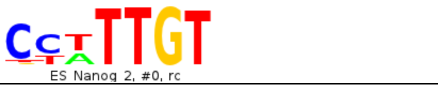
ES_n-Myc	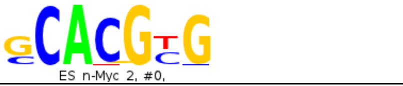	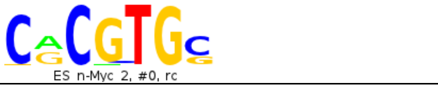
ES_Nr5a2	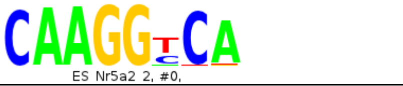	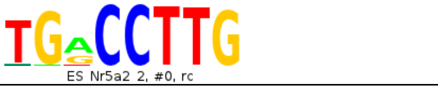
ES_Oct4	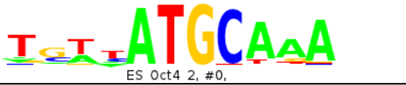	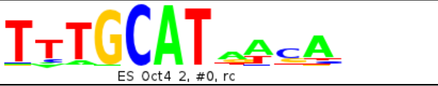
ES_P300	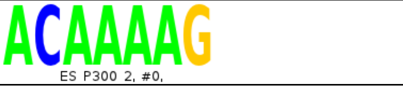	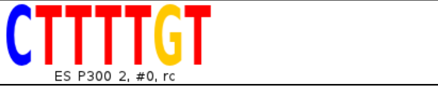
ES_Smad1	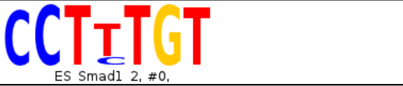	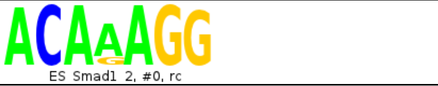
ES_Sox2	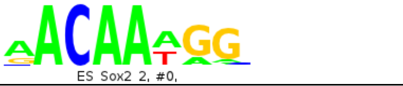	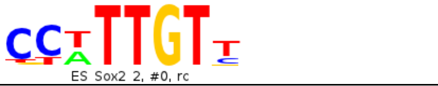
ES_STAT3	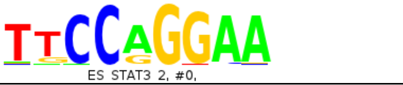	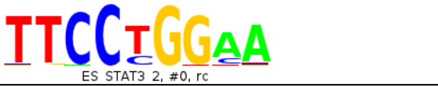
ES_Suz12	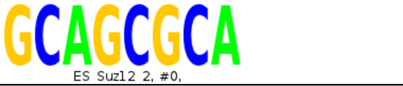	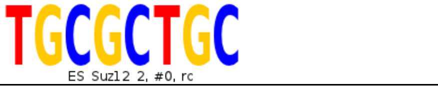
ES_Tcfcp2l1	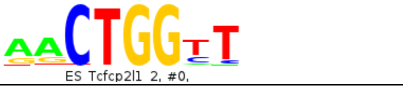	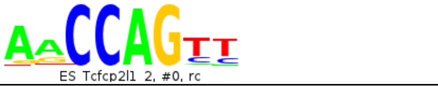
ES_Zfx	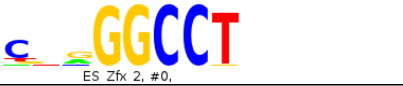	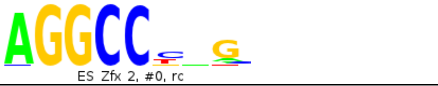
