# Supplementary material for: High Resolution Genome Wide Binding Event Finding and Motif Discovery Reveals Transcription Factor Spatial Binding Constraints
Source: PLoS Comput Biol. 2012 Aug 9;8(8):e1002638. doi: 10.1371/journal.pcbi.1002638 (PMC3415389; doi:10.1371/journal.pcbi.1002638)
Supplement: Table S6 — All significant pairwise spatial binding constraints detected from mouse ES cell ChIP-Seq data. (PDF) [file pcbi.1002638.s023.pdf]

Table S6 All significant pairwise spatial binding constraints detected from mouse ES cell ChIP-Seq data

TF1	TF2	Count at strongest spacing	Distance of strongest spacing	Max -log10(p-value)	# Significant Positions
c-Myc	E2f1	49	3	14.9	2
c-Myc	Klf4	33	1	15.7	2
c-Myc	n-Myc	1246	0	300.0	4
Ctcf	E2f1	79	6	67.5	3
Ctcf	Esrrb	34	57	10.0	3
Ctcf	Tcfcp2l1	22	2	8.4	1
E2f1	Ctcf	62	6	46.4	3
E2f1	Klf4	248	3	195.8	2
E2f1	n-Myc	157	1	93.9	7
E2f1	Zfx	148	6	61.4	2
Esrrb	Klf4	164	31	115.5	43
Esrrb	Nanog	79	58	74.6	3
Esrrb	n-Myc	42	23	9.6	1
Esrrb	Nr5a2	2352	1	300.0	4
Esrrb	Oct4	30	24	10.8	10
Esrrb	Sox2	76	56	62.4	25
Esrrb	Tcfcp2l1	316	10	259.4	85
Klf4	Ctcf	24	9	15.0	2
Klf4	Esrrb	166	31	117.7	40
Klf4	Nanog	93	24	141.0	1
Klf4	n-Myc	72	1	45.5	4
Klf4	Nr5a2	33	30	31.7	3
Klf4	Oct4	20	27	9.0	1
Klf4	Sox2	119	25	186.2	6
Klf4	Tcfcp2l1	119	41	94.6	4
Klf4	Zfx	66	5	26.2	1
Nanog	Klf4	95	24	144.4	1
Nanog	Nr5a2	35	57	41.7	5
Nanog	Oct4	276	7	300.0	12
Nanog	P300	48	2	102.0	1
Nanog	Smad1	43	1	97.2	1
Nanog	Sox2	1595	1	300.0	3
Nanog	Tcfcp2l1	41	65	34.8	4
Nr5a2	Sox2	30	58	32.7	4
Nr5a2	Tcfcp2l1	57	11	58.5	2
Nr5a2	Zfx	29	0	26.8	1
Oct4	Sox2	482	6	300.0	50
P300	Sox2	51	1	99.6	1
Smad1	Sox2	49	0	108.8	1
Sox2	Smad1	49	0	111.3	1
Sox2	Tcfcp2l1	61	66	58.2	8
