# Supplementary material for: High Resolution Genome Wide Binding Event Finding and Motif Discovery Reveals Transcription Factor Spatial Binding Constraints
Source: PLoS Comput Biol. 2012 Aug 9;8(8):e1002638. doi: 10.1371/journal.pcbi.1002638 (PMC3415389; doi:10.1371/journal.pcbi.1002638)
Supplement: Table S7 — Non-redundant significant pairwise spatial binding constraints detected from mouse ES cell ChIP-Seq data, consolidated to non-redundant factor pairs. (PDF) [file pcbi.1002638.s024.pdf]

Table S7 Non-redundant significant pairwise spatial binding constraints detected from mouse ES cell ChIP-Seq data, consolidated to non-redundant factor pairs

TF1	TF2	Most significant spacing
Ctcf	E2f1	6
Ctcf	Esrrb	57
Ctcf	Klf4	9
Ctcf	Tcfcp2l1	2
E2f1	Klf4	3
E2f1	Zfx	6
E2f1	c-Myc	3
E2f1	n-Myc	1
Esrrb	Klf4	31
Esrrb	Nanog	58
Esrrb	Nr5a2	1
Esrrb	Oct4	24
Esrrb	Sox2	56
Esrrb	Tcfcp2l1	10
Esrrb	n-Myc	23
Klf4	Nanog	24
Klf4	Nr5a2	30
Klf4	Oct4	27
Klf4	Sox2	25
Klf4	Tcfcp2l1	41
Klf4	Zfx	5
Klf4	c-Myc	1
Klf4	n-Myc	1
Nanog	Nr5a2	57
Nanog	Oct4	7
Nanog	P300	2
Nanog	Smad1	1
Nanog	Sox2	1
Nanog	Tcfcp2l1	65
Nr5a2	Sox2	58
Nr5a2	Tcfcp2l1	11
Nr5a2	Zfx	0
Oct4	Sox2	6
P300	Sox2	1
Smad1	Sox2	0
Sox2	Tcfcp2l1	66
c-Myc	n-Myc	0

Total count: 37
